# Supplementary figures and images for: TRPML1 suppresses pulmonary fibrosis by limiting collagen and elastin deposition
Source: EMBO J. 2026 Feb 19;45(7):2182–209. doi: 10.1038/s44318-026-00712-4 (PMC13043727; doi:10.1038/s44318-026-00712-4)

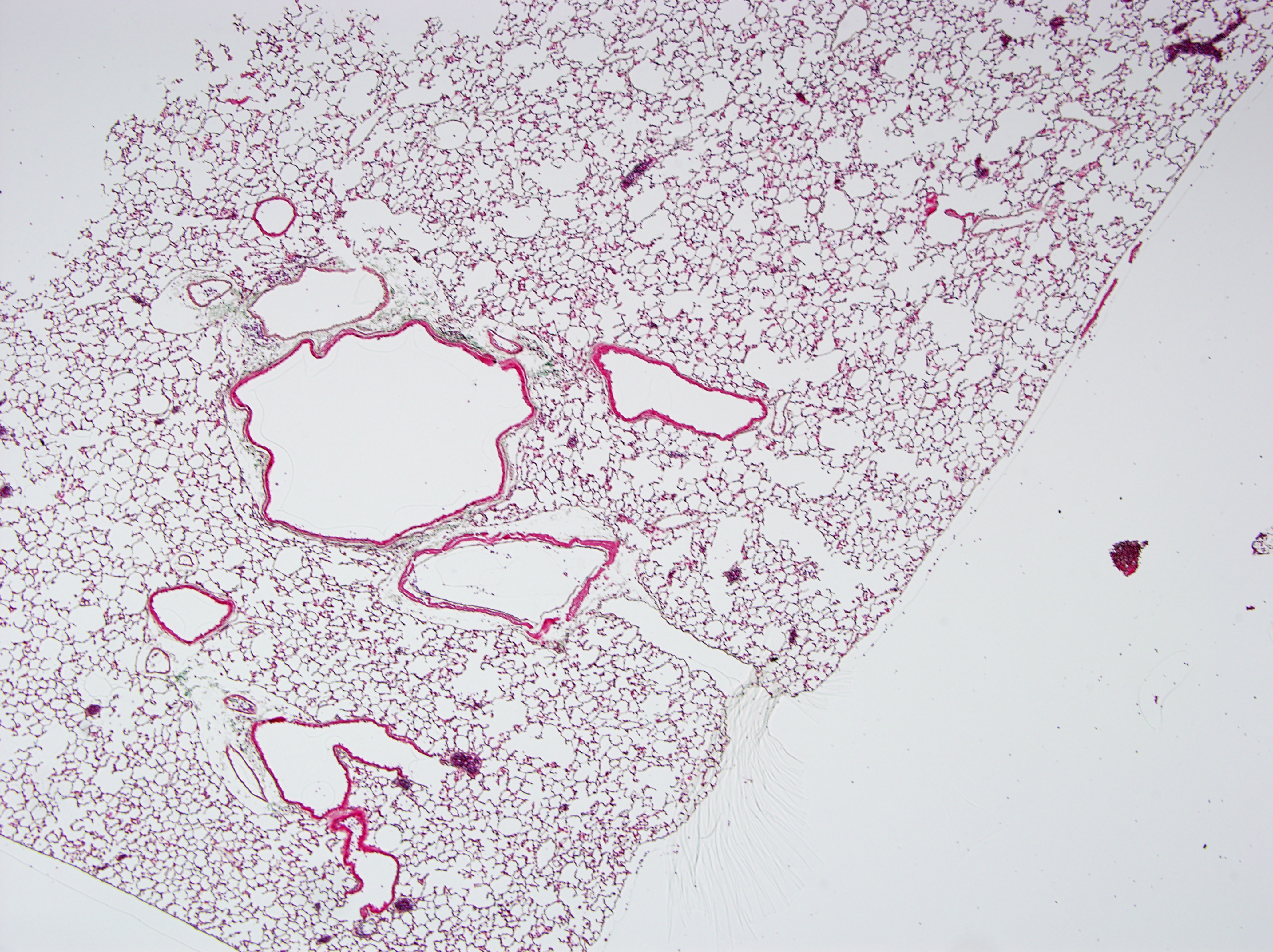

Supplement: Supplementary file 2 — Source data Fig. 1 [file 44318_2026_712_MOESM2_ESM.zip › Figure 1/1C/HZ 4x.tif]

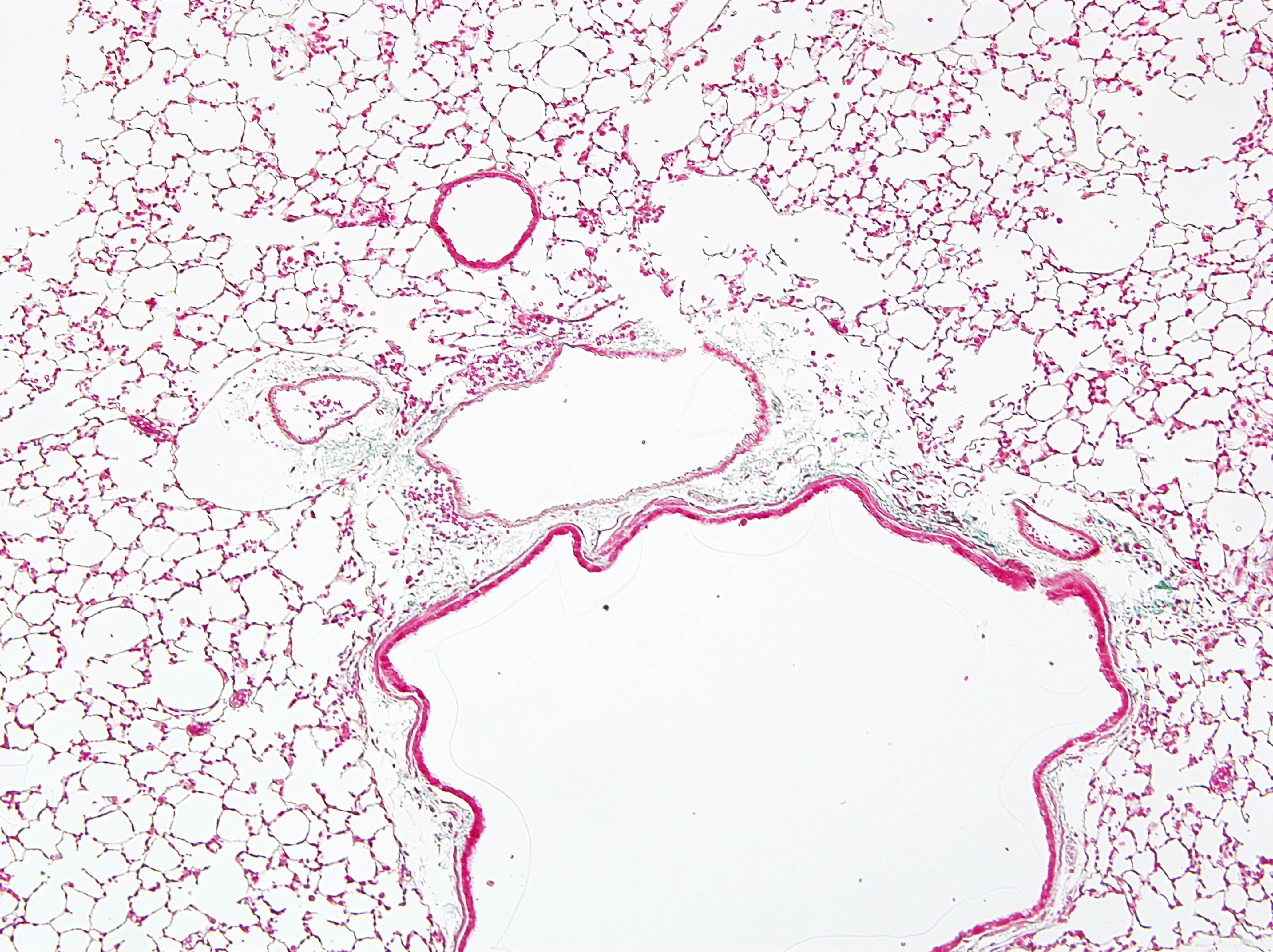

Supplement: Supplementary file 2 — Source data Fig. 1 [file 44318_2026_712_MOESM2_ESM.zip › Figure 1/1C/HZ_10x.tif]

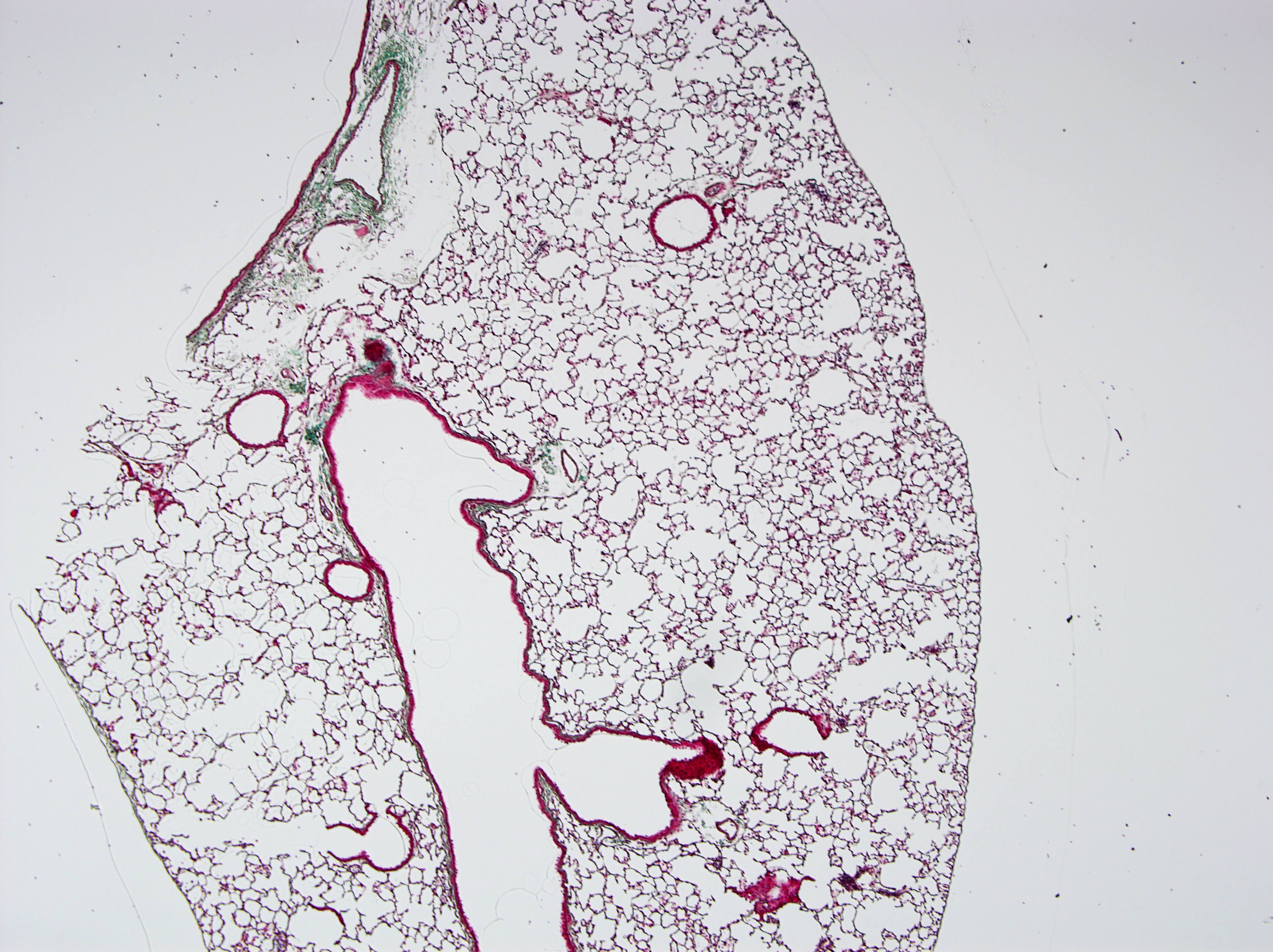

Supplement: Supplementary file 2 — Source data Fig. 1 [file 44318_2026_712_MOESM2_ESM.zip › Figure 1/1C/KO 4x.tif]

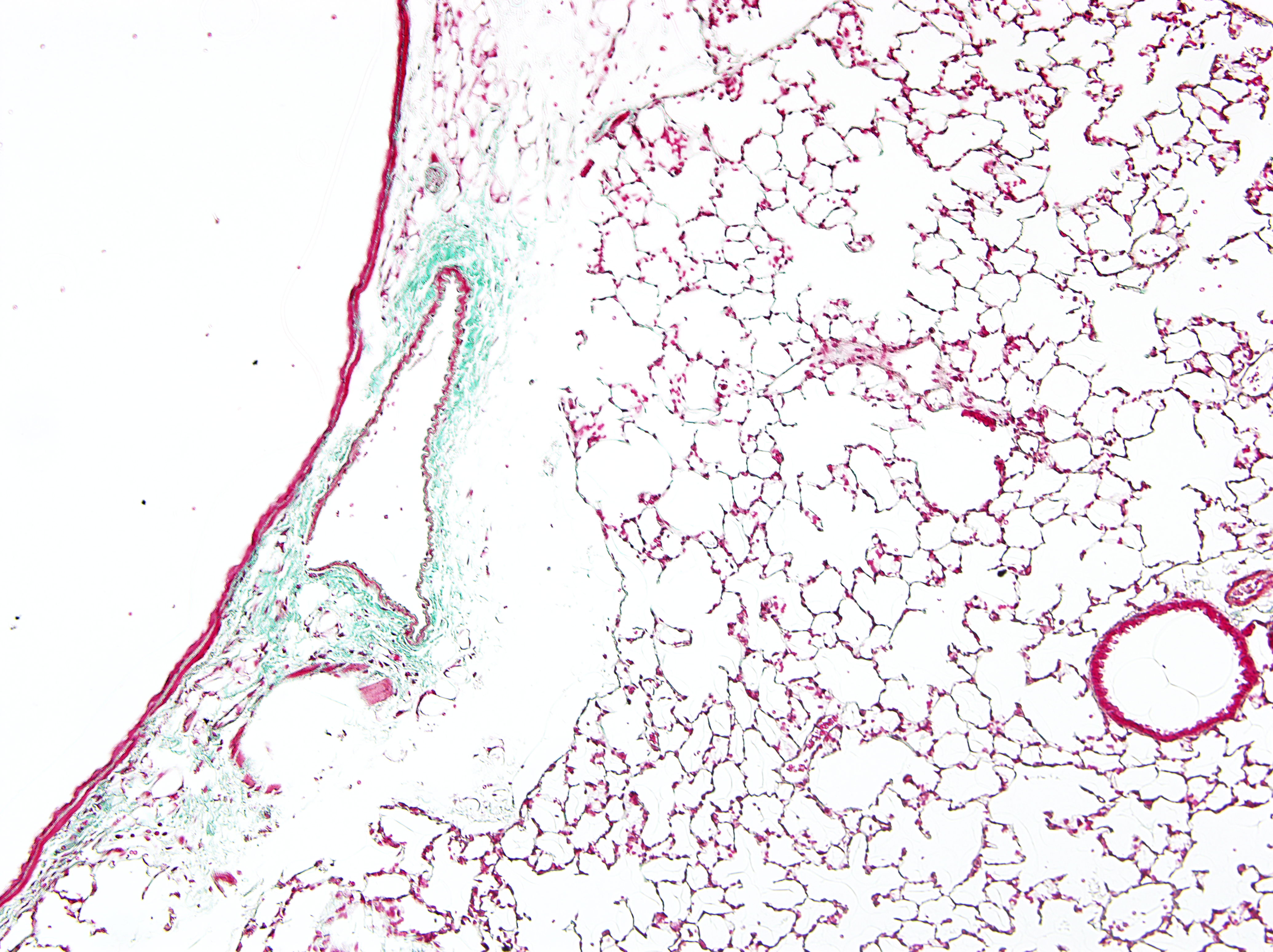

Supplement: Supplementary file 2 — Source data Fig. 1 [file 44318_2026_712_MOESM2_ESM.zip › Figure 1/1C/KO_10x.tif]

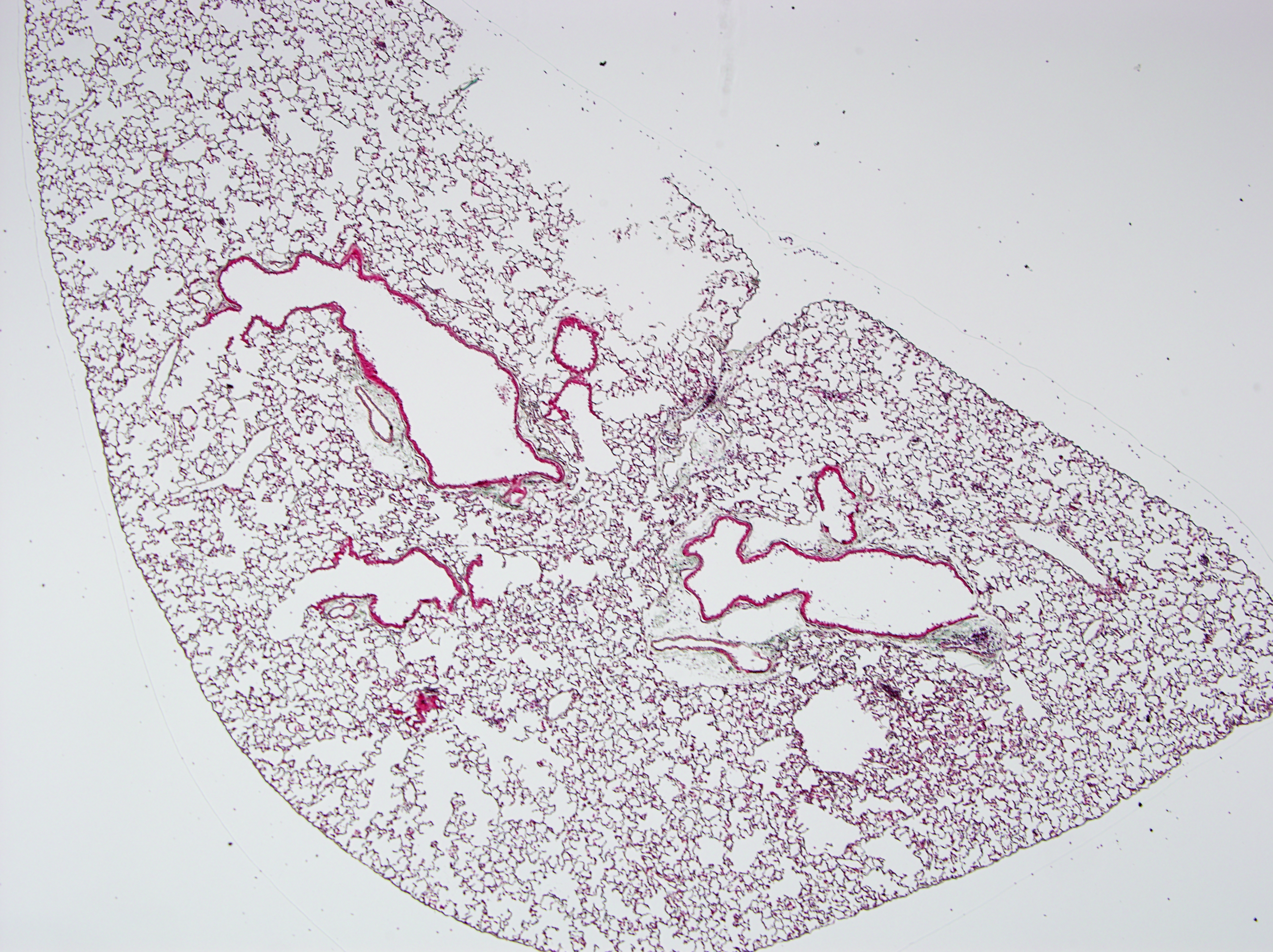

Supplement: Supplementary file 2 — Source data Fig. 1 [file 44318_2026_712_MOESM2_ESM.zip › Figure 1/1C/WT 4x.tif]

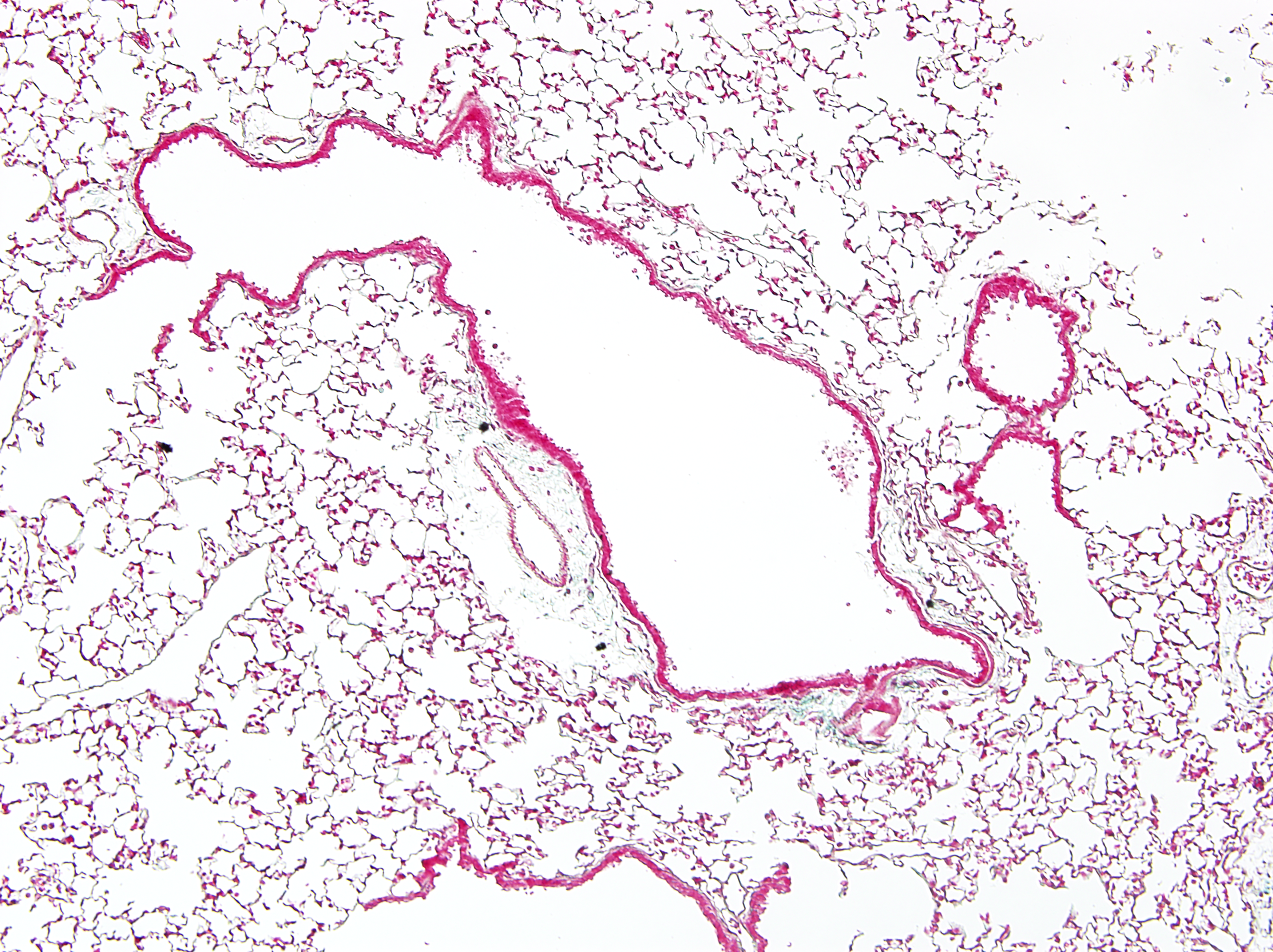

Supplement: Supplementary file 2 — Source data Fig. 1 [file 44318_2026_712_MOESM2_ESM.zip › Figure 1/1C/WT_10x.tif]

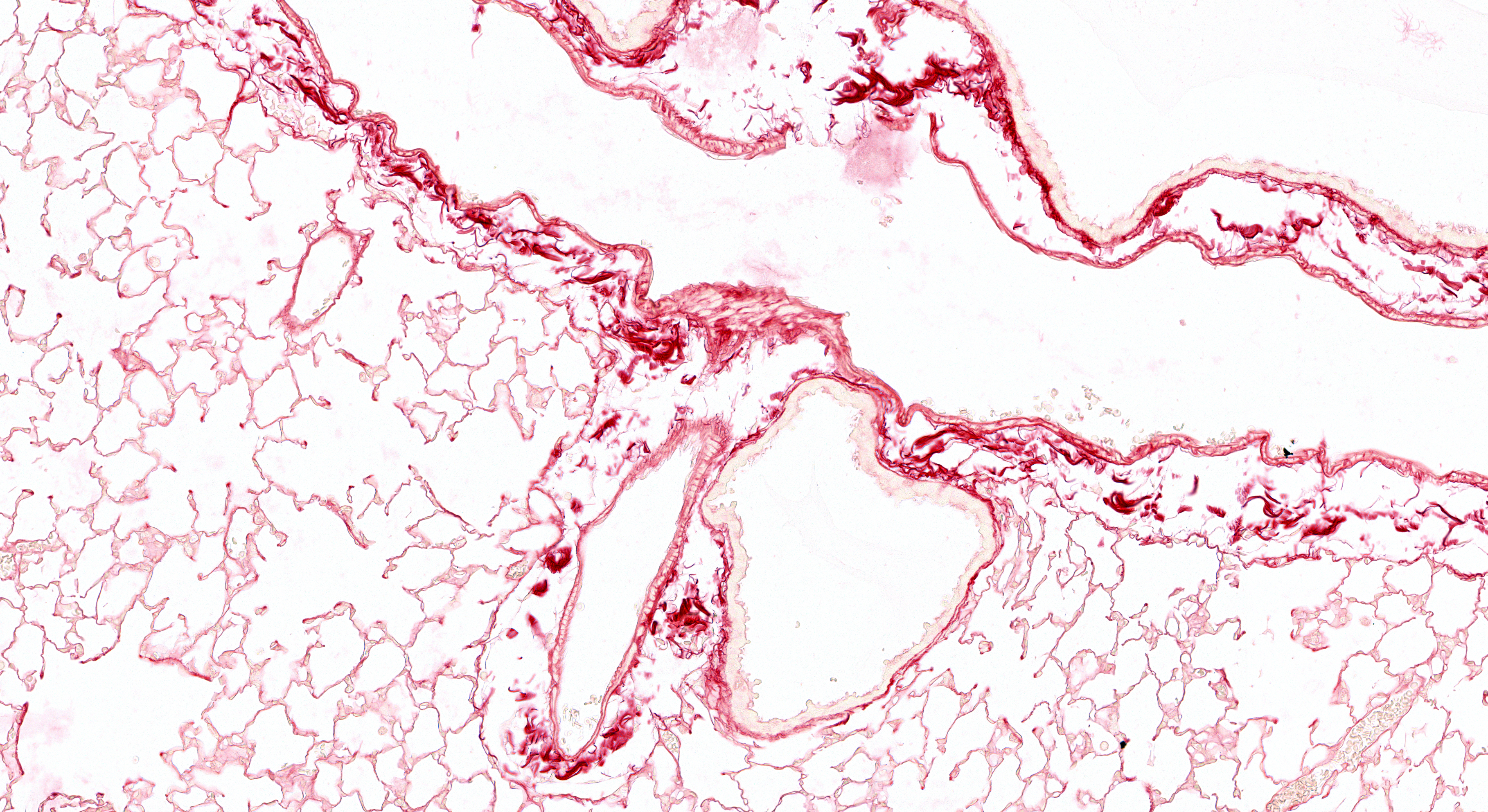

Supplement: Supplementary file 2 — Source data Fig. 1 [file 44318_2026_712_MOESM2_ESM.zip › Figure 1/1E/HZ_20x_area3.tif]

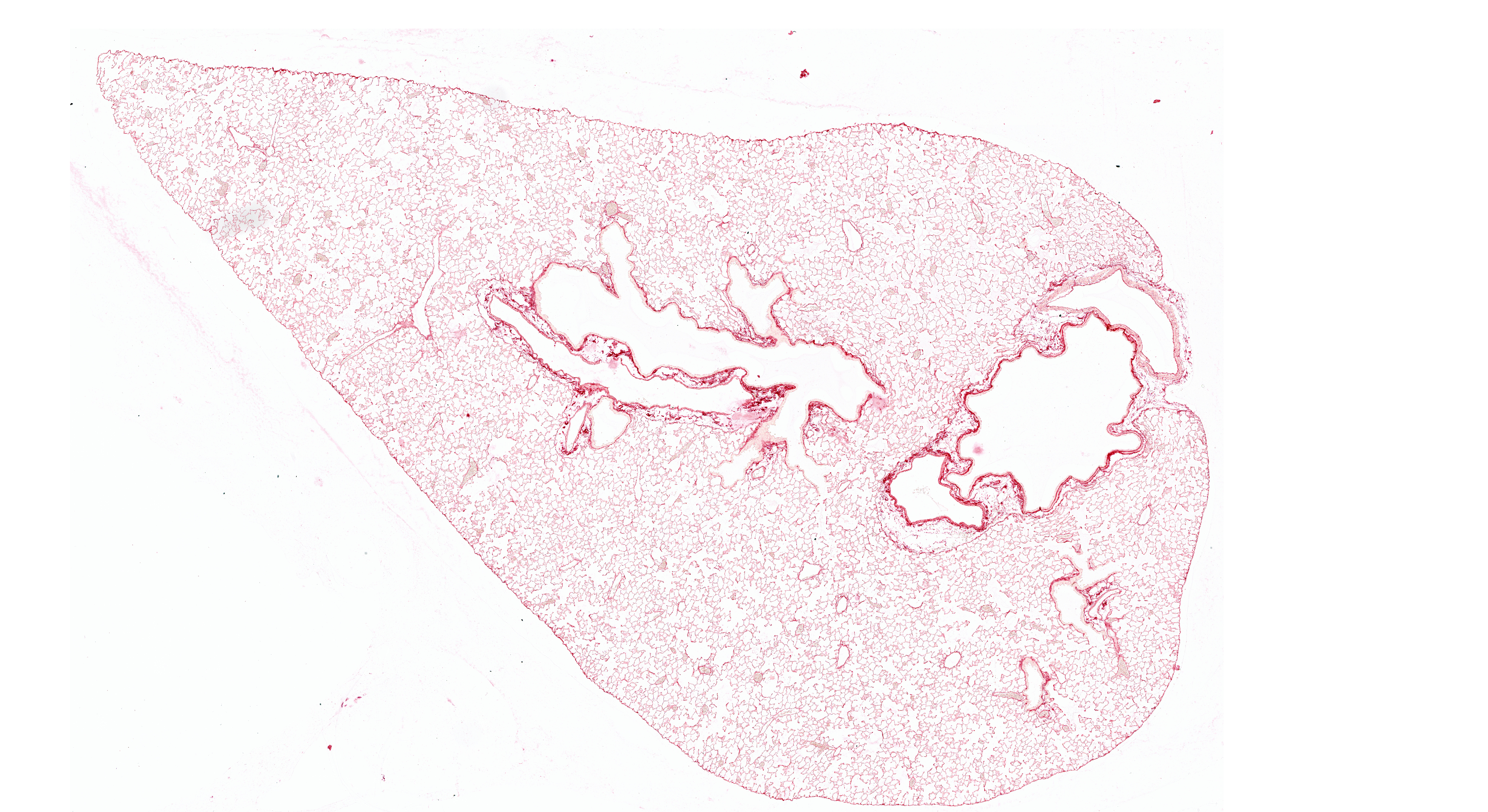

Supplement: Supplementary file 2 — Source data Fig. 1 [file 44318_2026_712_MOESM2_ESM.zip › Figure 1/1E/HZ_2x_area3.tif]

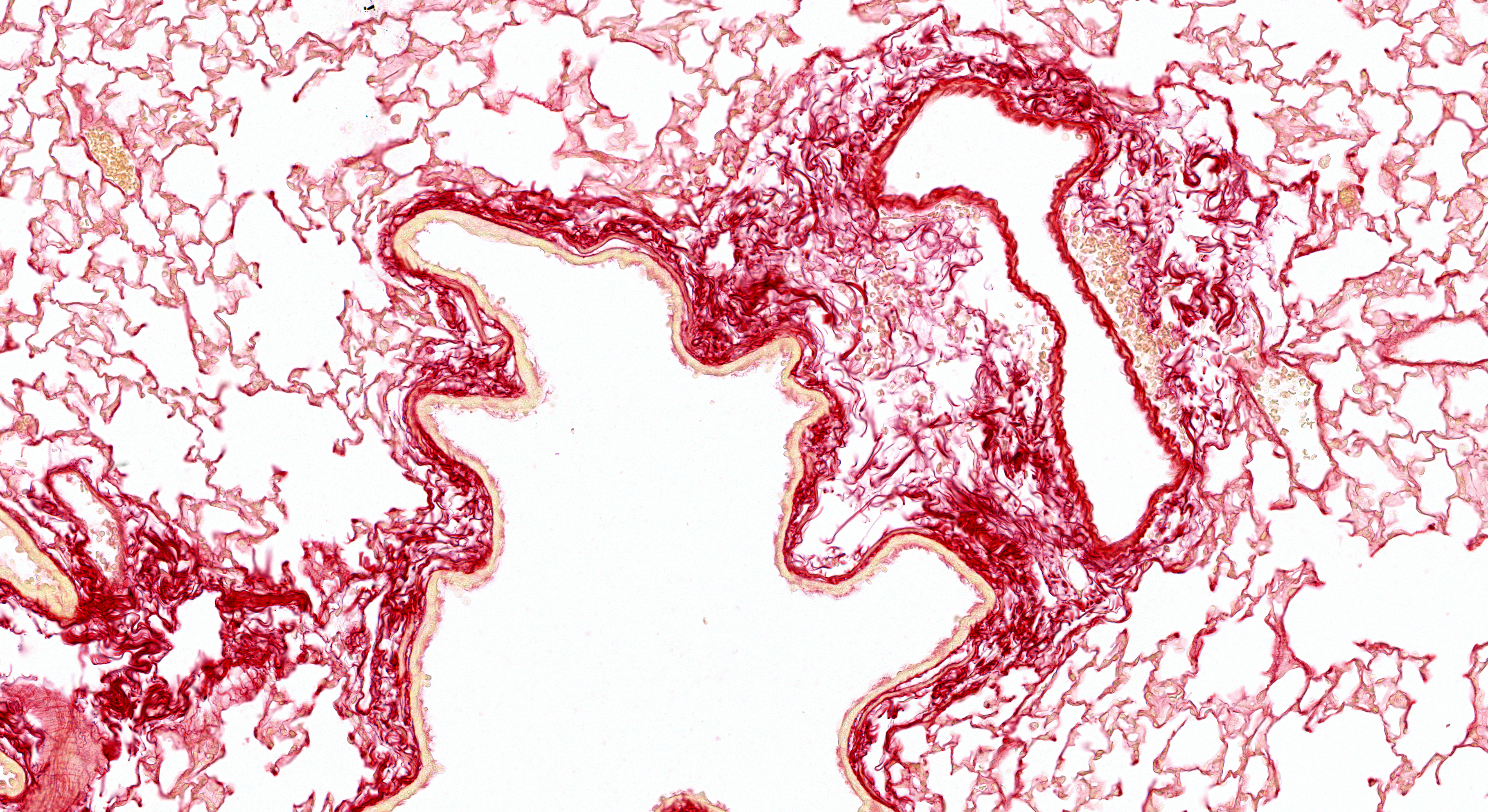

Supplement: Supplementary file 2 — Source data Fig. 1 [file 44318_2026_712_MOESM2_ESM.zip › Figure 1/1E/KO_20x_area1.tif]

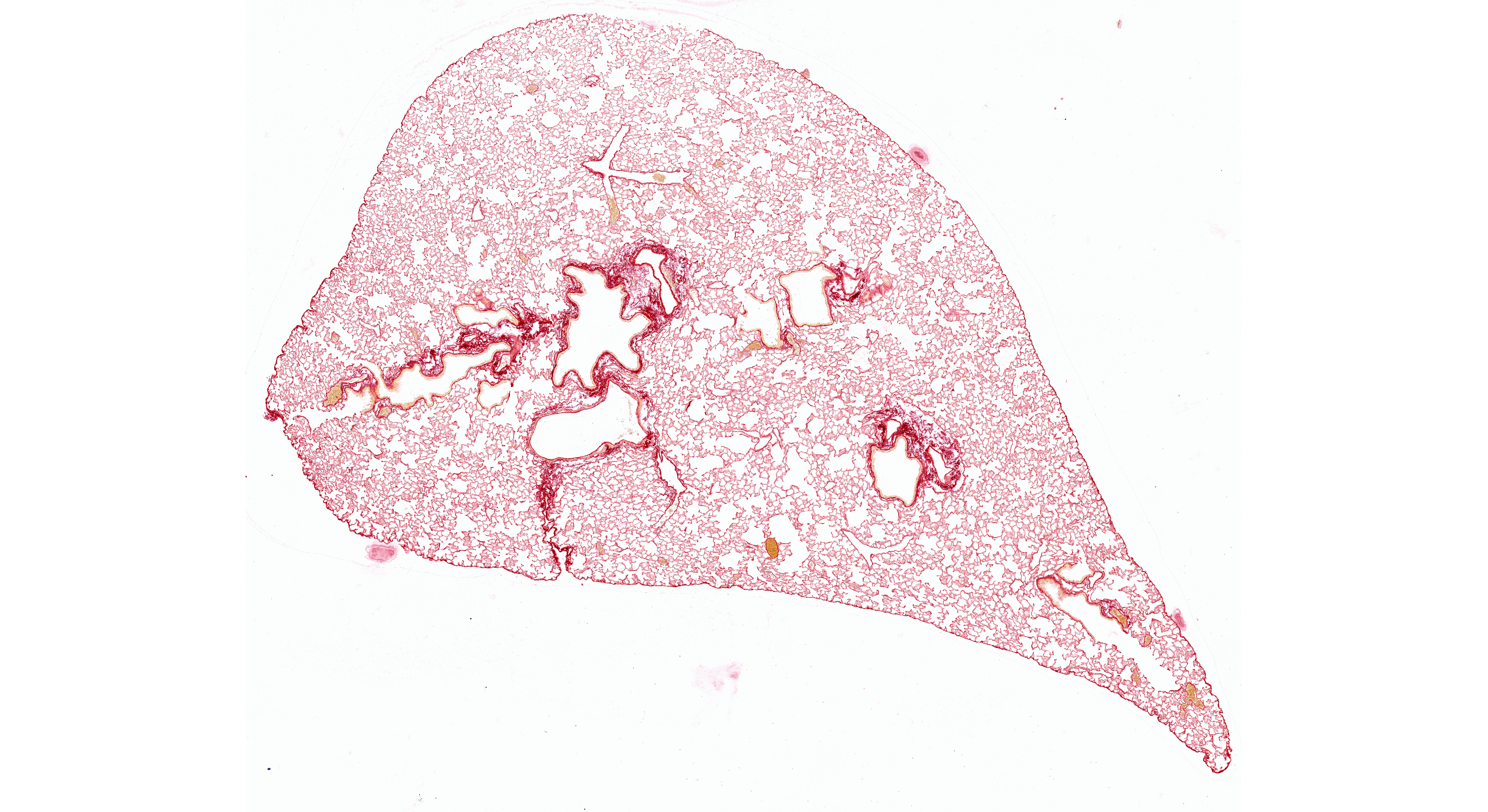

Supplement: Supplementary file 2 — Source data Fig. 1 [file 44318_2026_712_MOESM2_ESM.zip › Figure 1/1E/KO_2x_area1.tif]

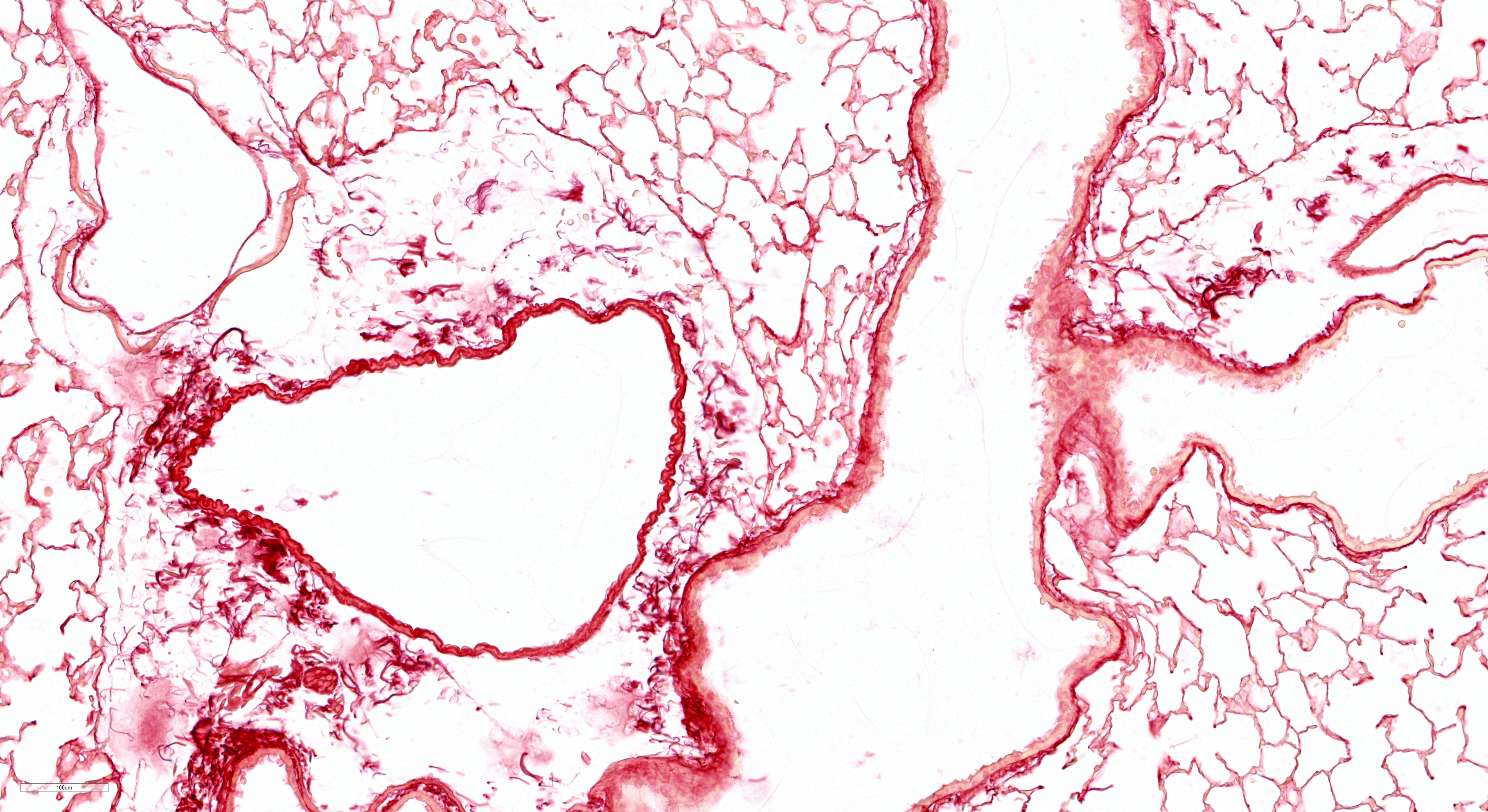

Supplement: Supplementary file 2 — Source data Fig. 1 [file 44318_2026_712_MOESM2_ESM.zip › Figure 1/1E/WT_20x_area2.tif]

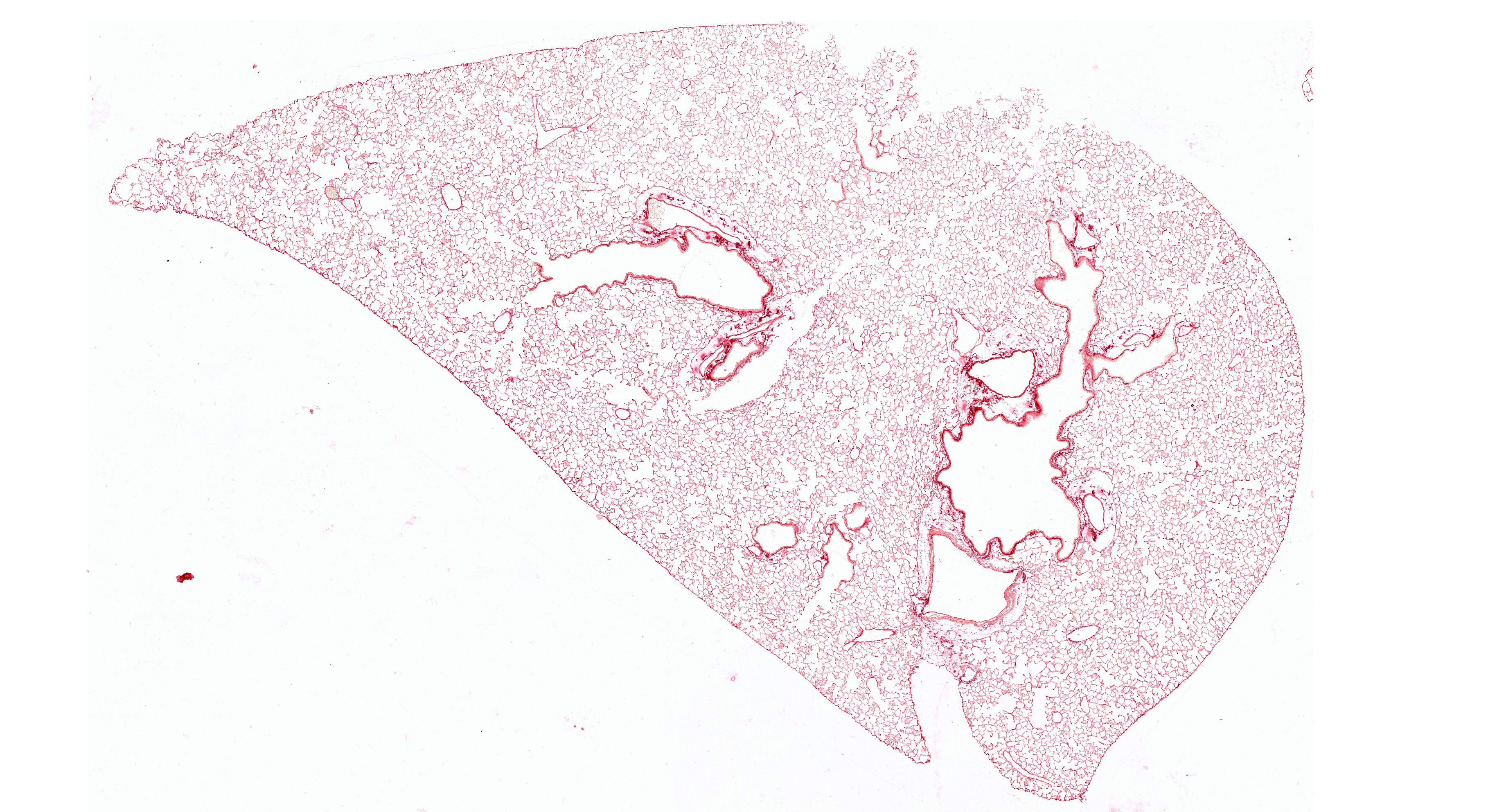

Supplement: Supplementary file 2 — Source data Fig. 1 [file 44318_2026_712_MOESM2_ESM.zip › Figure 1/1E/WT_2x_area2.tif]

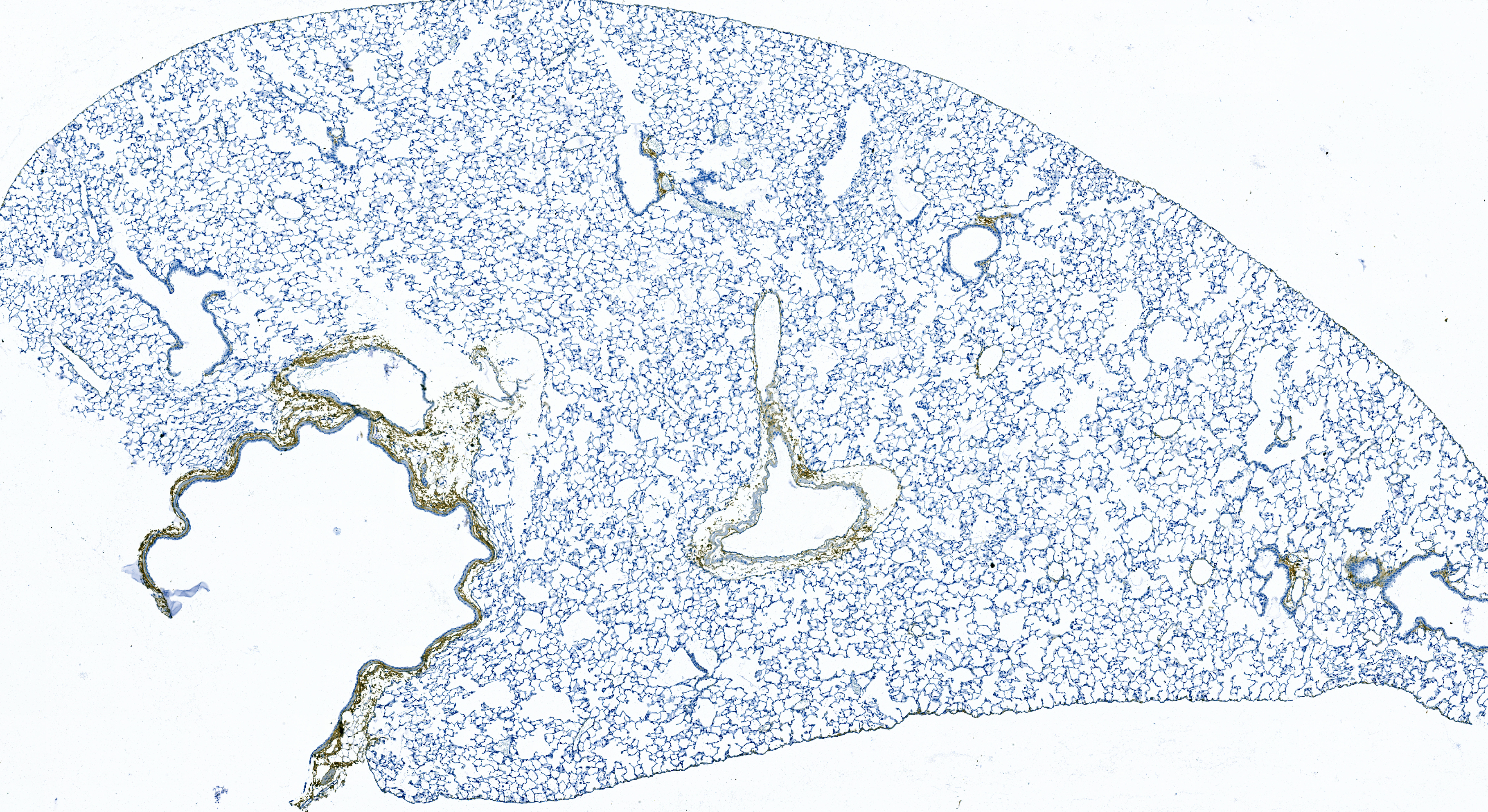

Supplement: Supplementary file 2 — Source data Fig. 1 [file 44318_2026_712_MOESM2_ESM.zip › Figure 1/1G/F49_WT_Col1a1_Übersicht.tif]

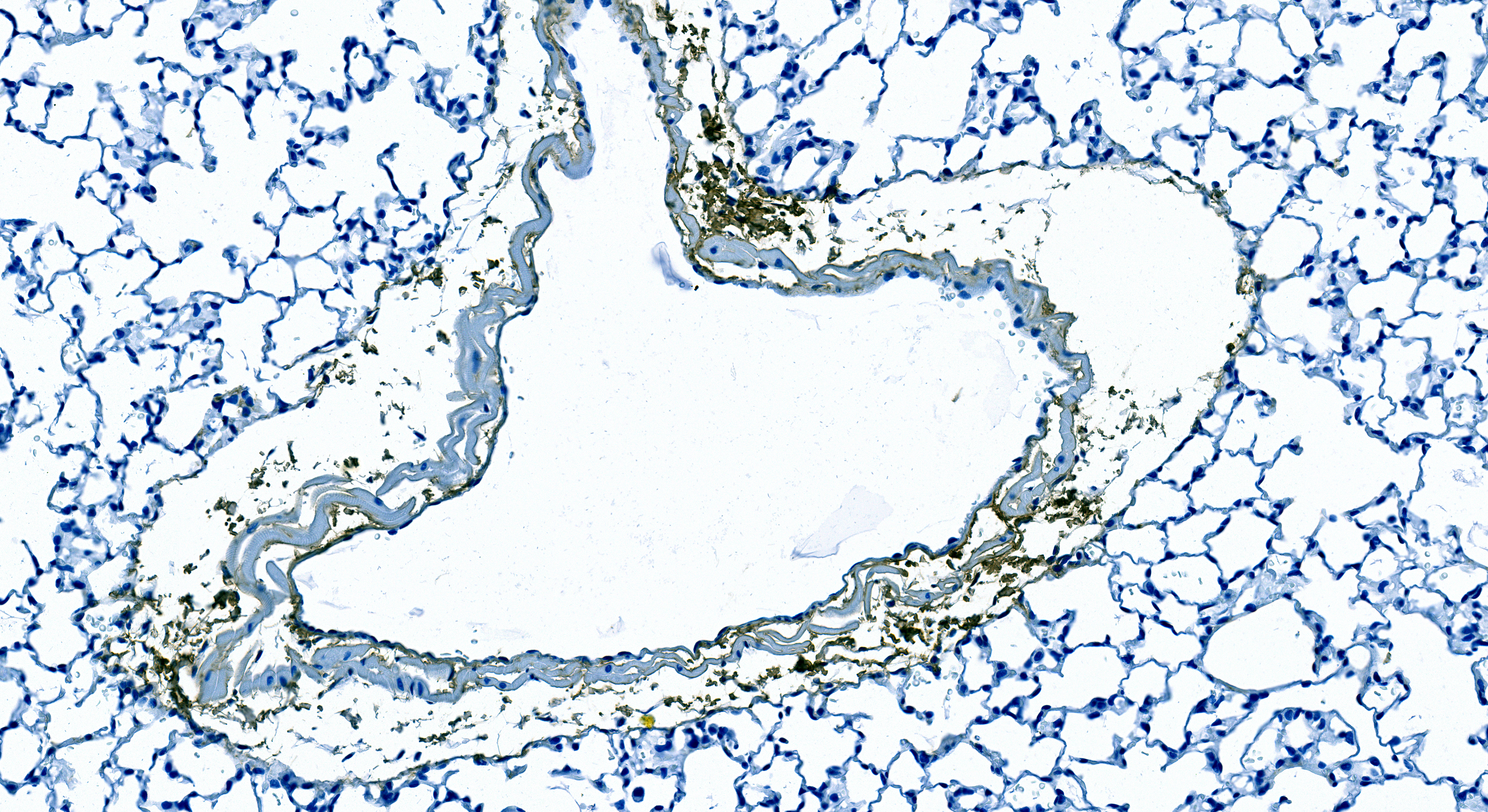

Supplement: Supplementary file 2 — Source data Fig. 1 [file 44318_2026_712_MOESM2_ESM.zip › Figure 1/1G/F49WT_Col1a1_20x1.tif]

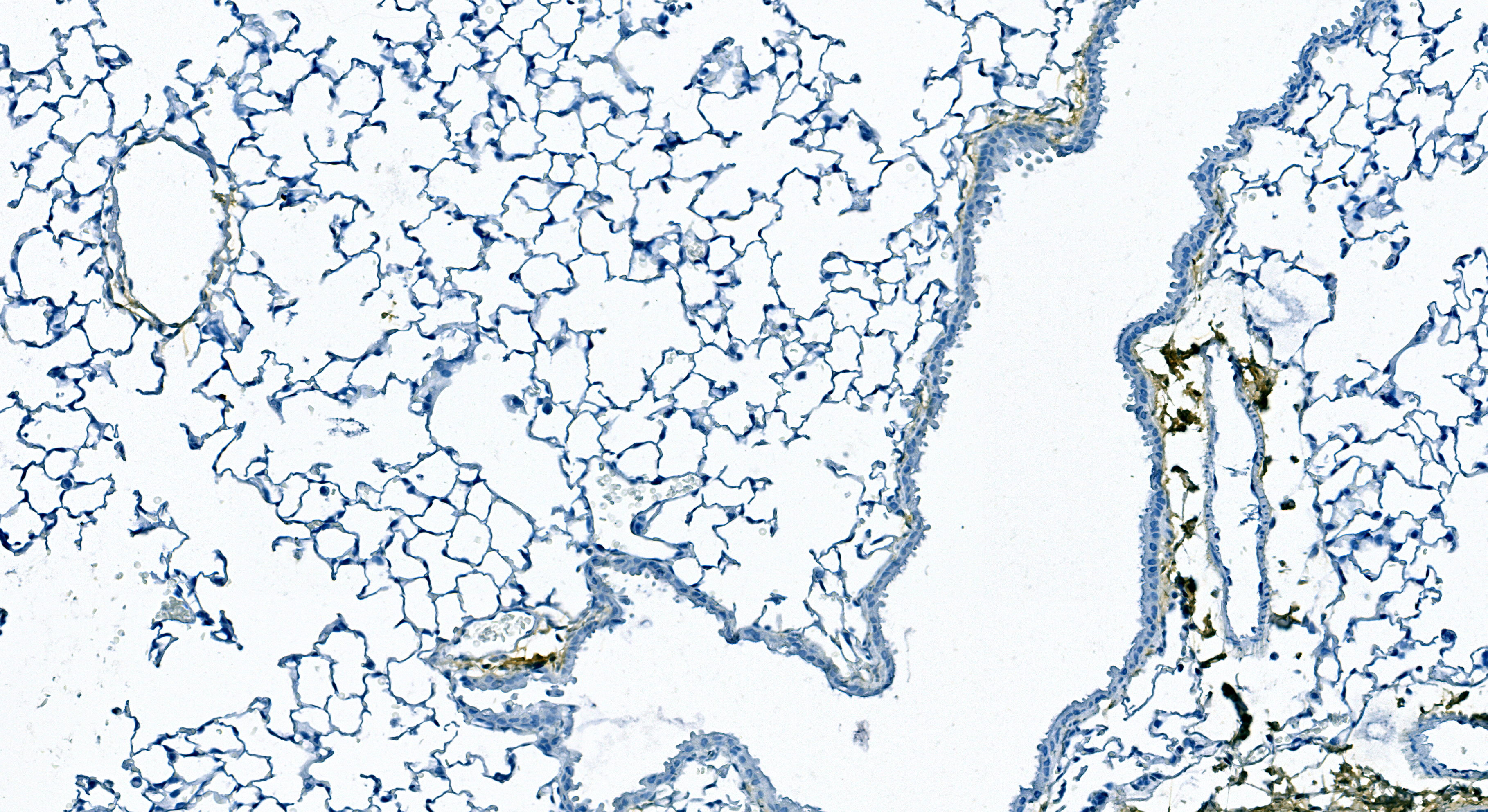

Supplement: Supplementary file 2 — Source data Fig. 1 [file 44318_2026_712_MOESM2_ESM.zip › Figure 1/1G/F72_HZ_Col1a1_20x2.tif]

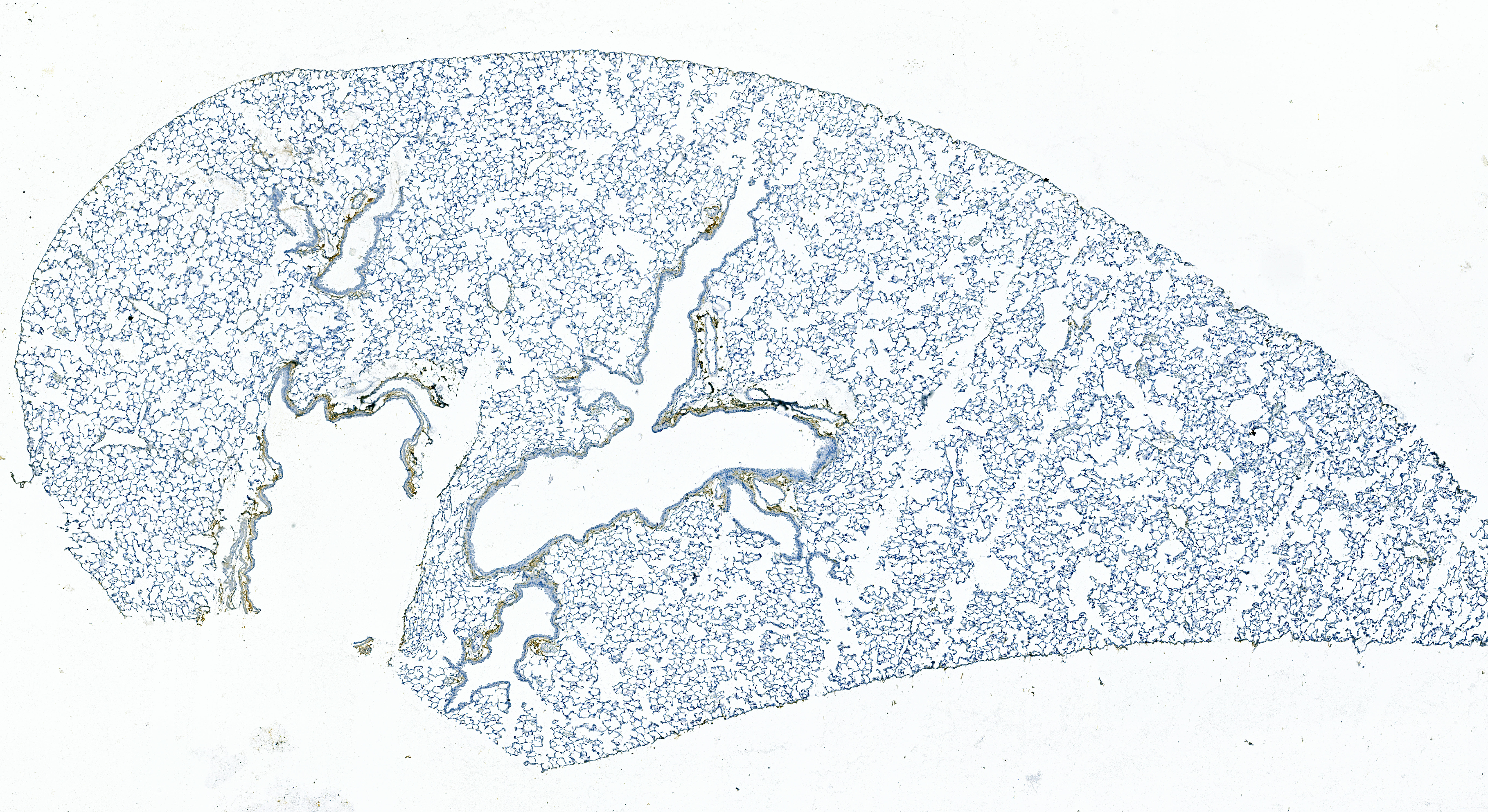

Supplement: Supplementary file 2 — Source data Fig. 1 [file 44318_2026_712_MOESM2_ESM.zip › Figure 1/1G/F72_HZ_Col1a1_Übersicht.tif]

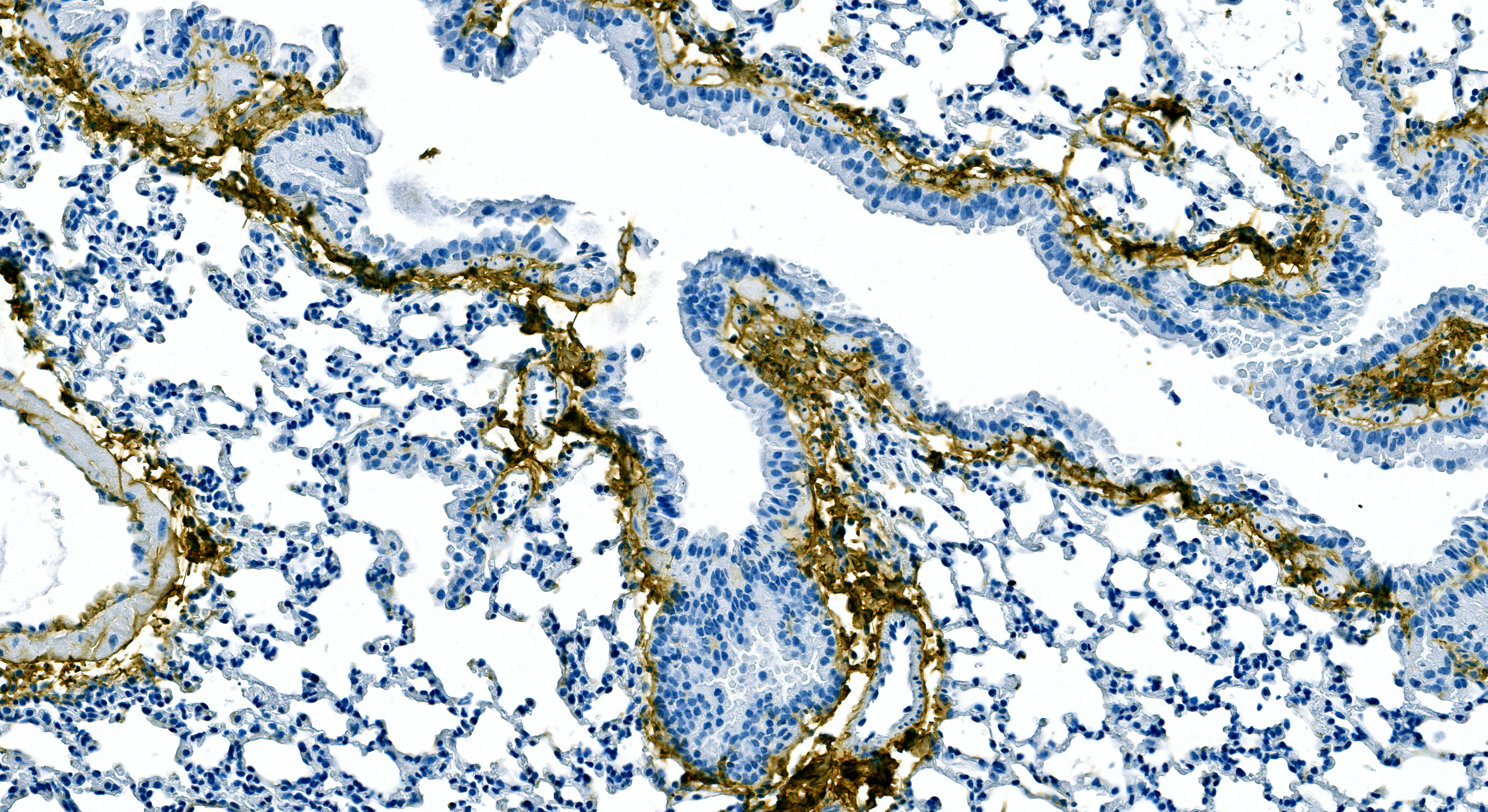

Supplement: Supplementary file 2 — Source data Fig. 1 [file 44318_2026_712_MOESM2_ESM.zip › Figure 1/1G/F86_KO_Col1a1_20x2.tif]

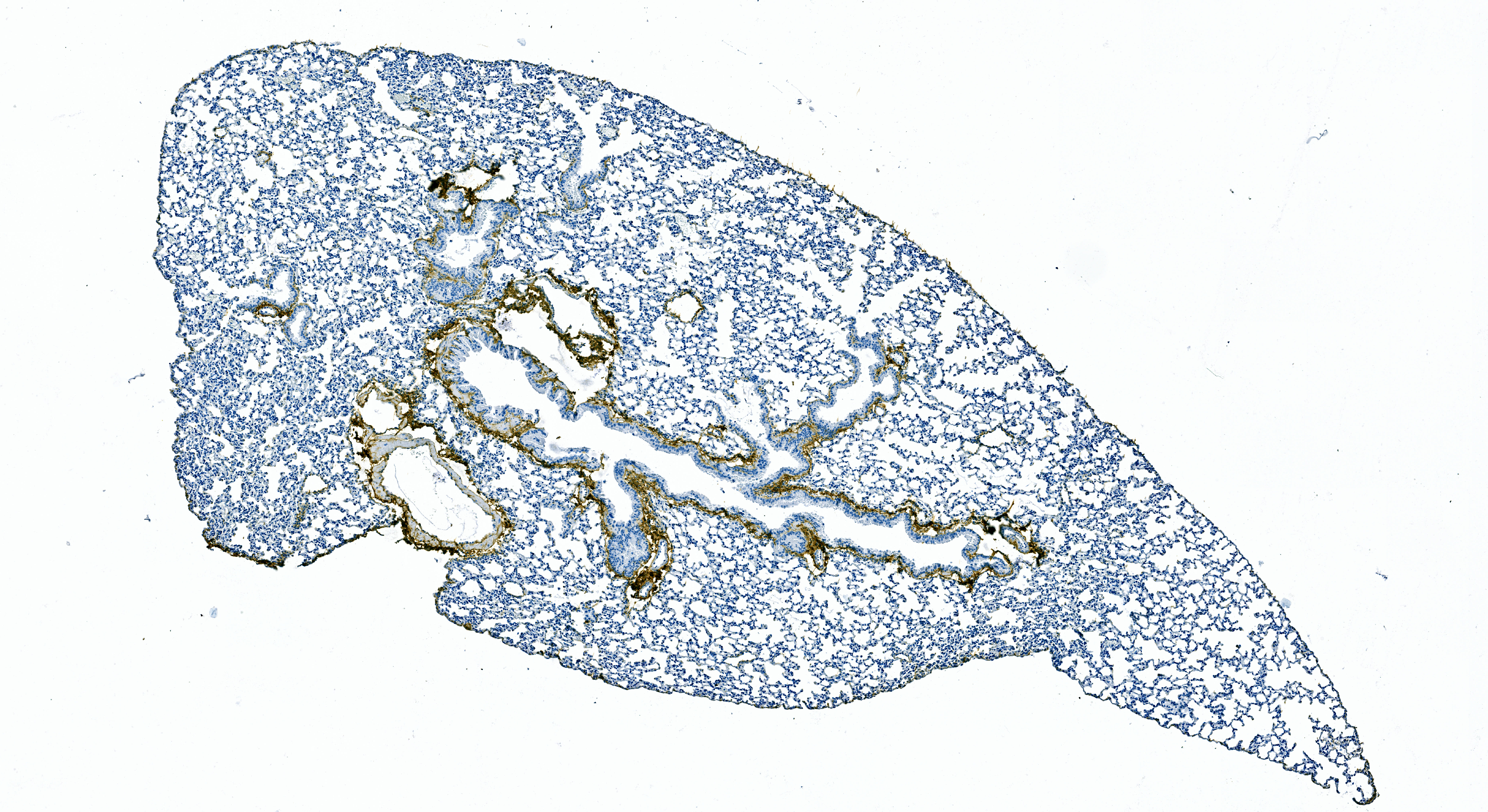

Supplement: Supplementary file 2 — Source data Fig. 1 [file 44318_2026_712_MOESM2_ESM.zip › Figure 1/1G/F86_KO_Col1a1_Übersicht.tif]

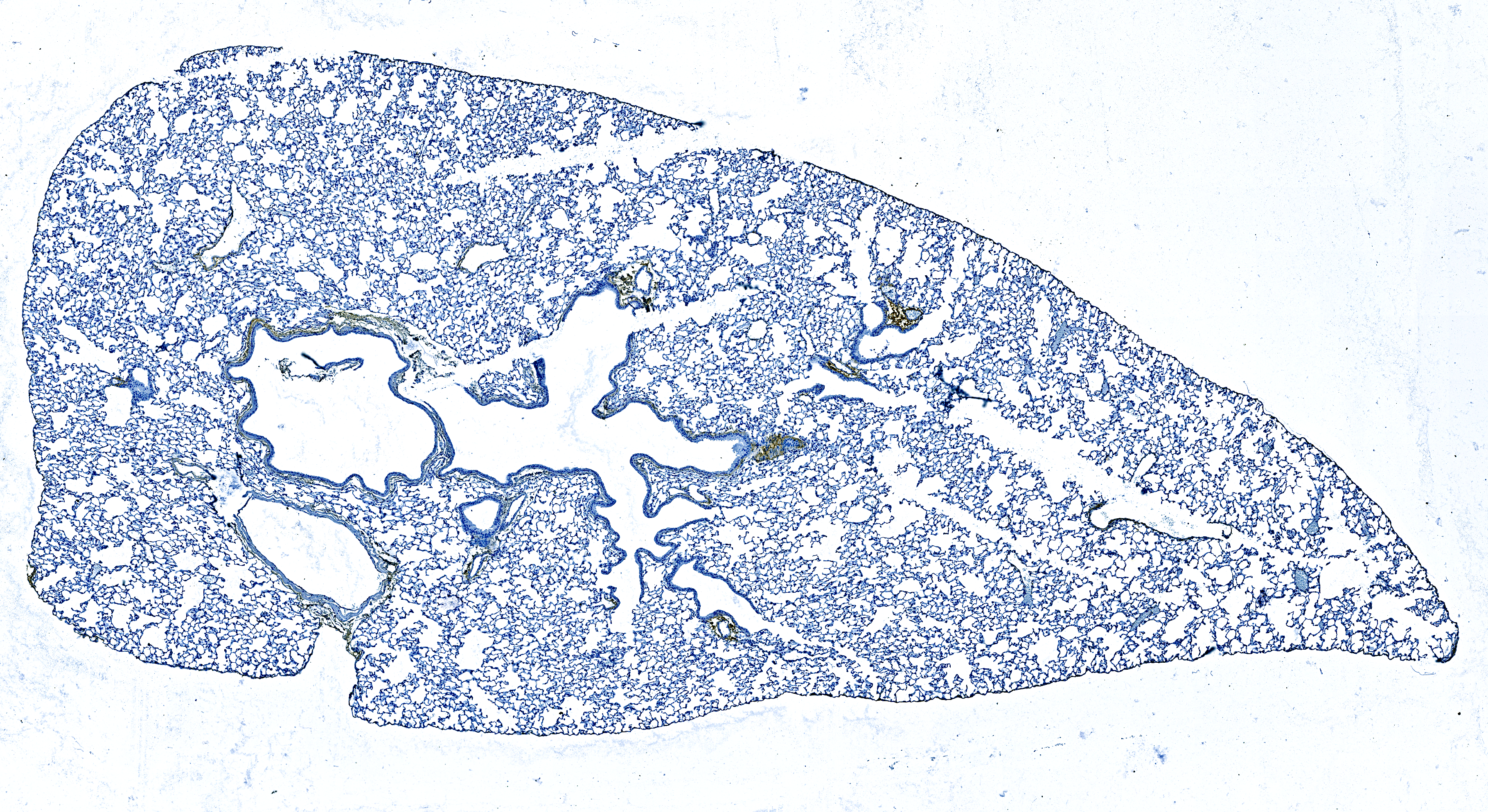

Supplement: Supplementary file 3 — Source data Fig. 2 [file 44318_2026_712_MOESM3_ESM.zip › Figure 2/2G/1a7_WT_PBS.tif]

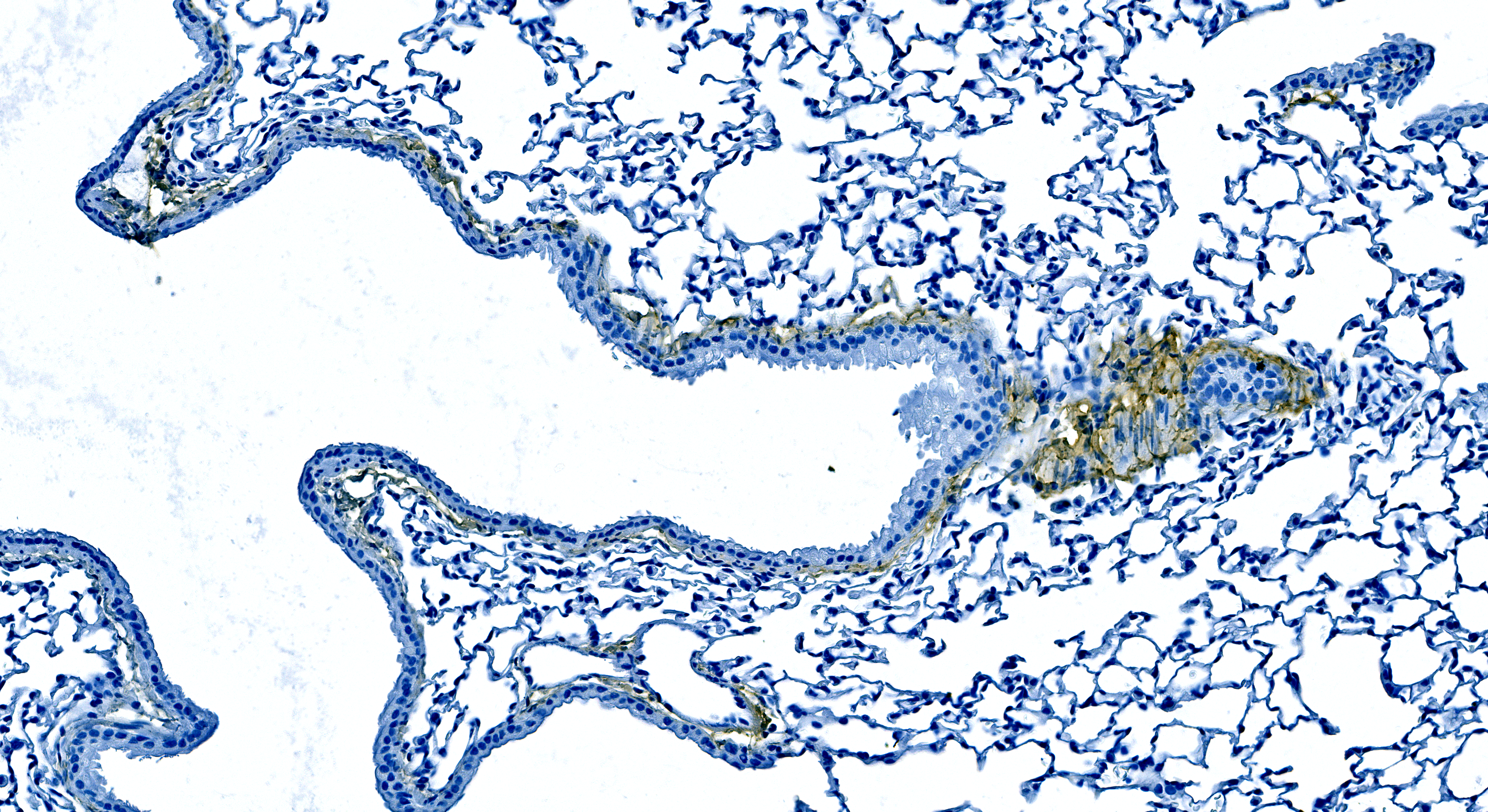

Supplement: Supplementary file 3 — Source data Fig. 2 [file 44318_2026_712_MOESM3_ESM.zip › Figure 2/2G/1a7_WT_PBS_20x.tif]

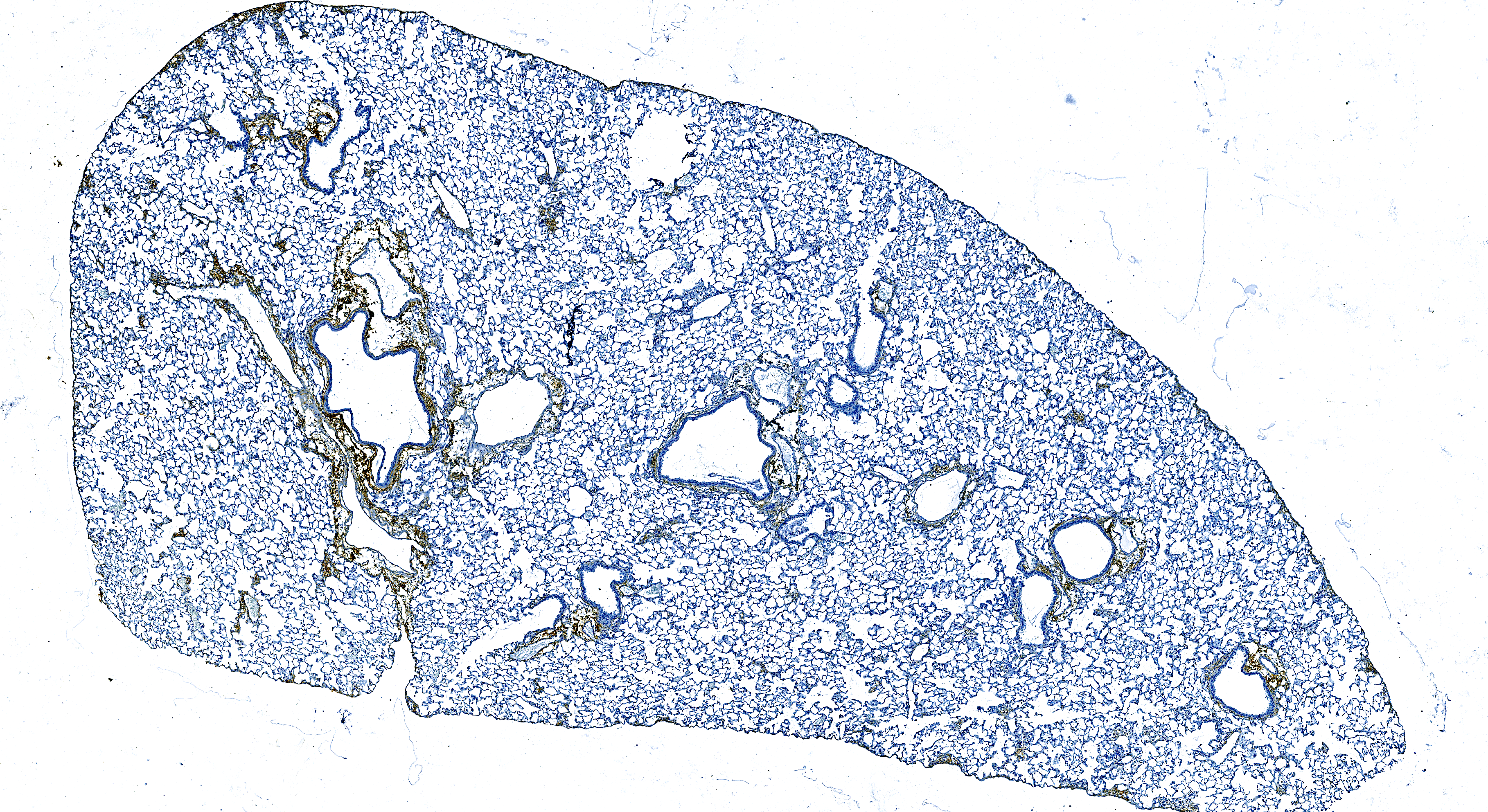

Supplement: Supplementary file 3 — Source data Fig. 2 [file 44318_2026_712_MOESM3_ESM.zip › Figure 2/2G/1b4_WT_Bleo.tif]

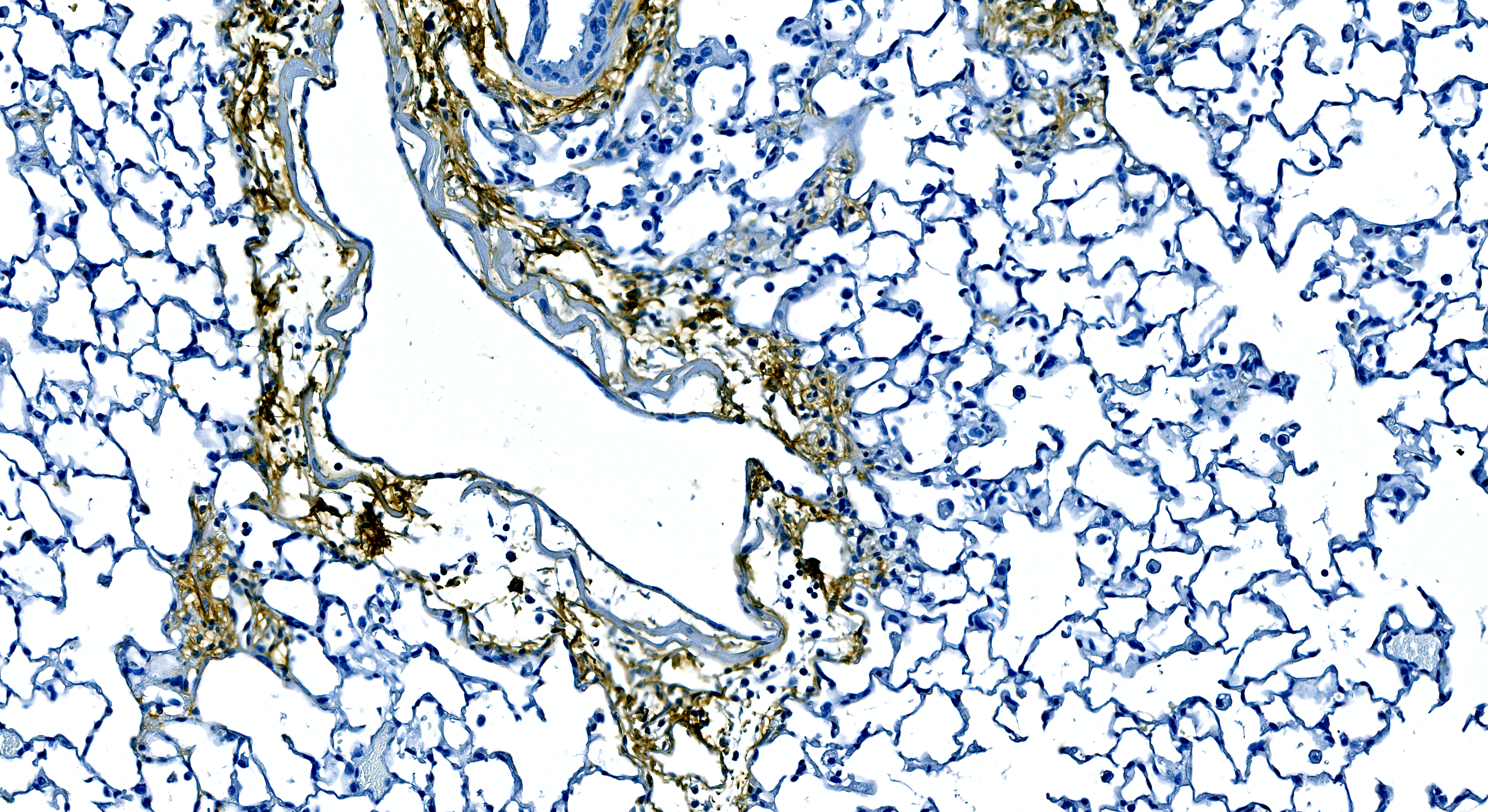

Supplement: Supplementary file 3 — Source data Fig. 2 [file 44318_2026_712_MOESM3_ESM.zip › Figure 2/2G/1b4_WT_Bleo_20x.tif]

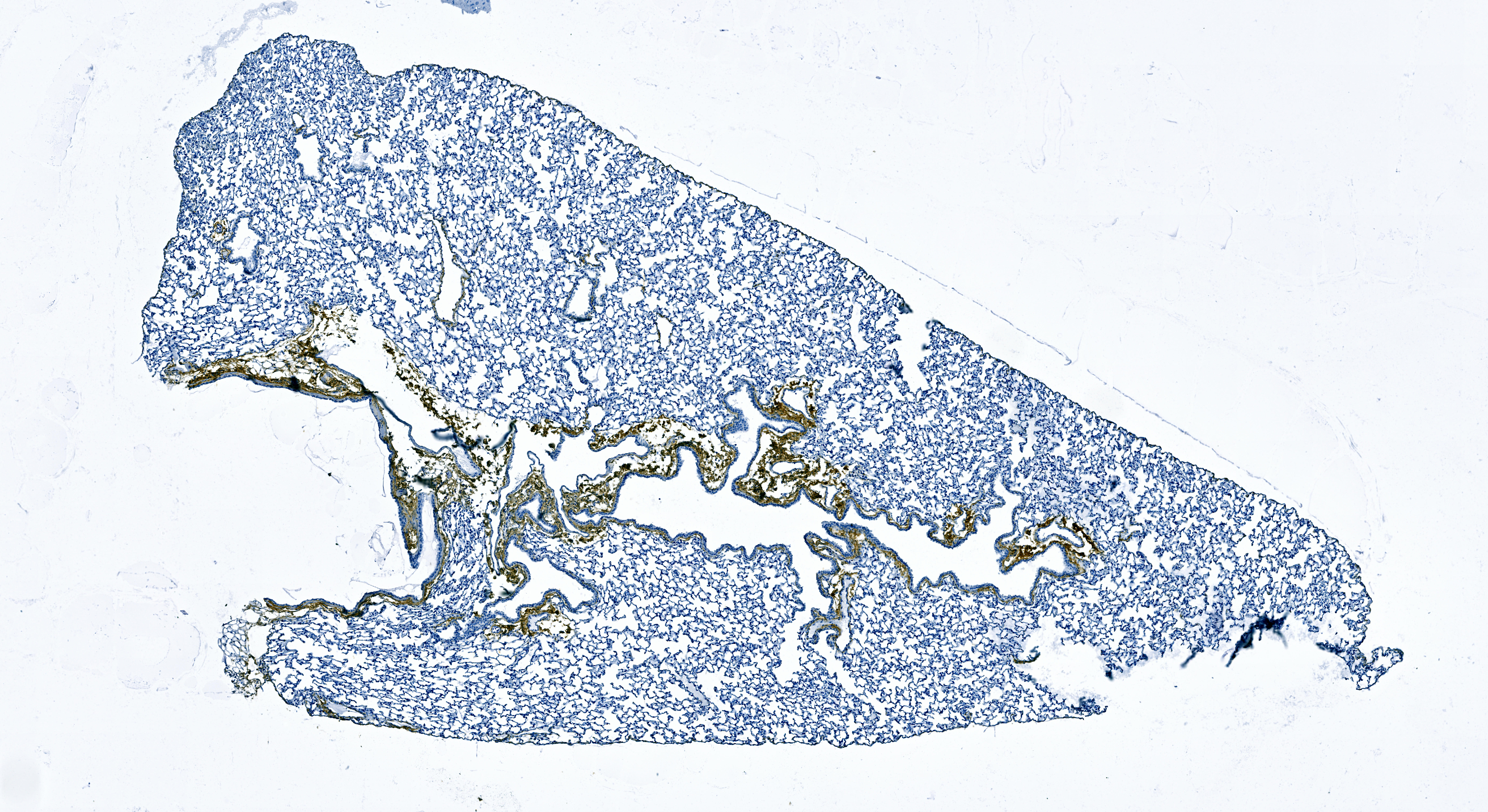

Supplement: Supplementary file 3 — Source data Fig. 2 [file 44318_2026_712_MOESM3_ESM.zip › Figure 2/2G/2a7_KO_PBS.tif]

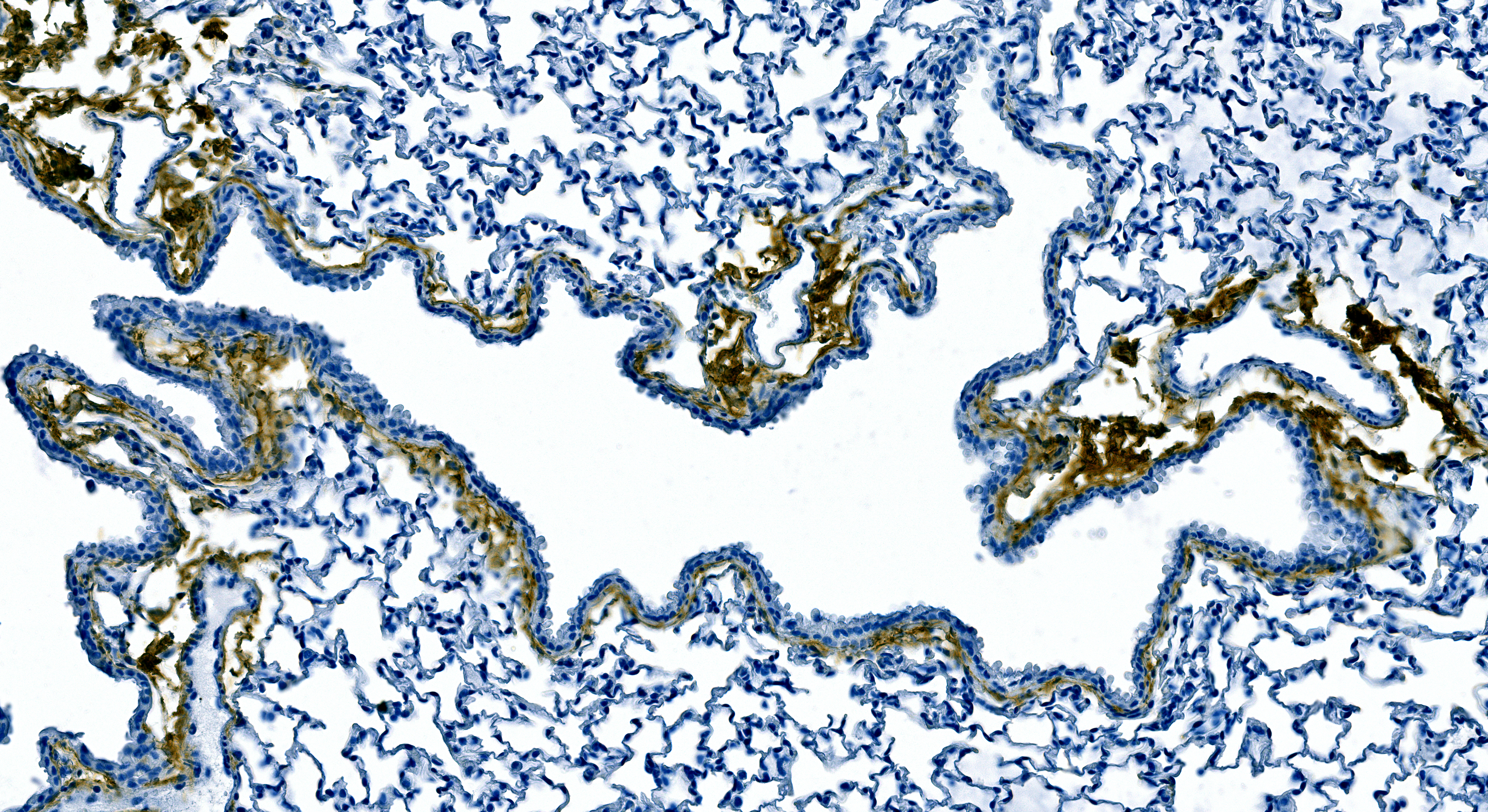

Supplement: Supplementary file 3 — Source data Fig. 2 [file 44318_2026_712_MOESM3_ESM.zip › Figure 2/2G/2a7_KO_PBS_20x.tif]

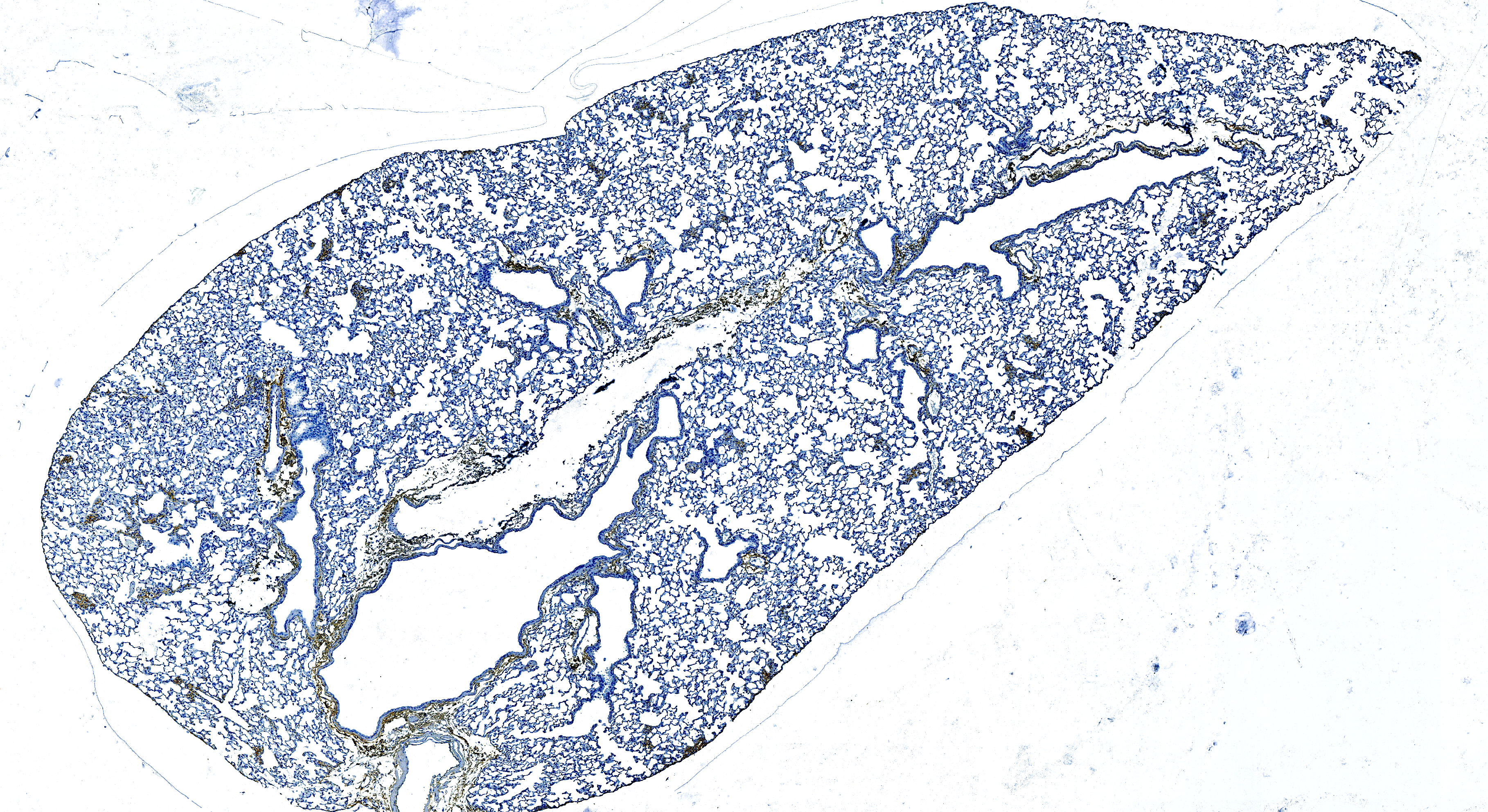

Supplement: Supplementary file 3 — Source data Fig. 2 [file 44318_2026_712_MOESM3_ESM.zip › Figure 2/2G/2b4_KO_Bleo.tif]

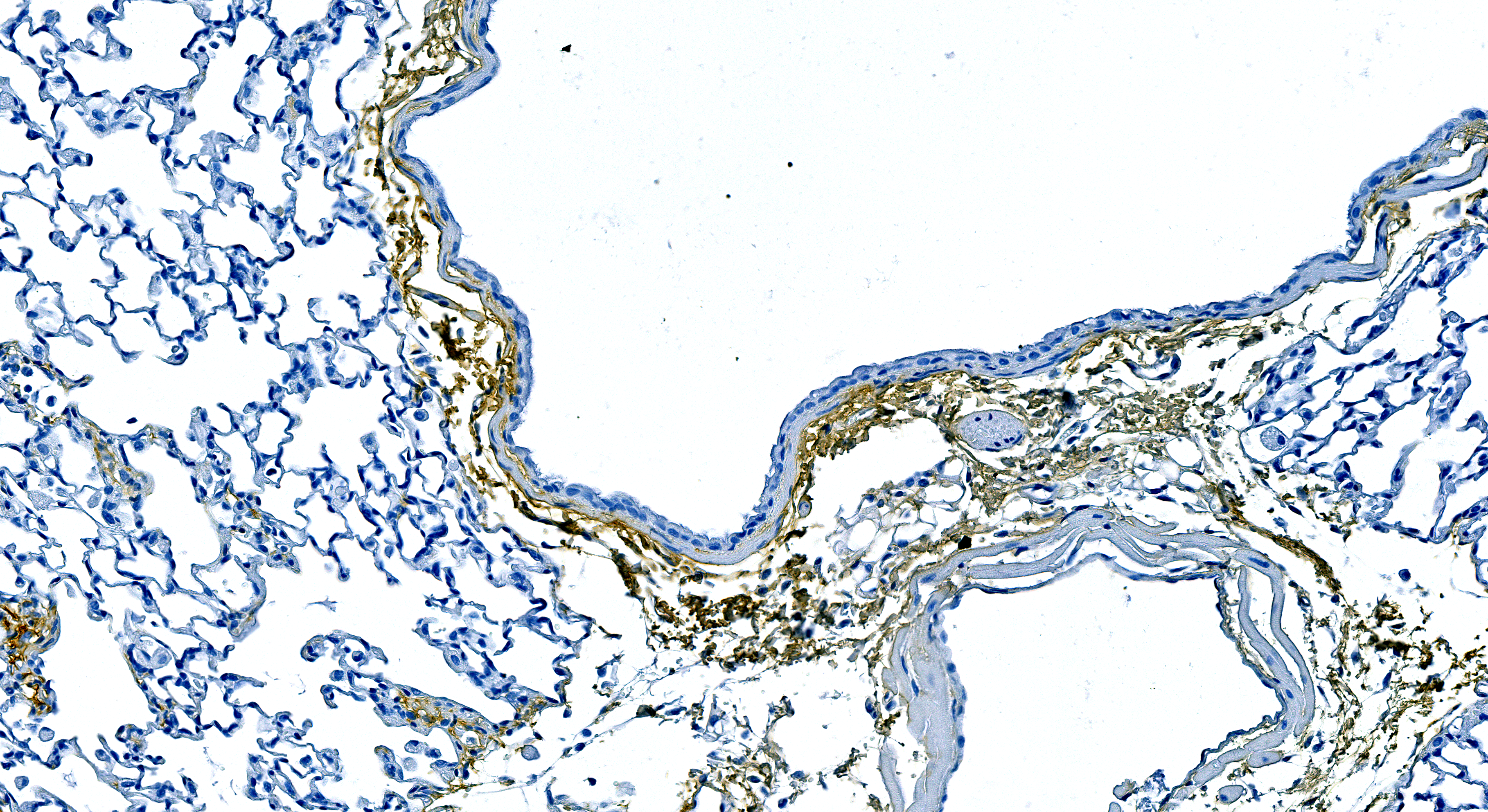

Supplement: Supplementary file 3 — Source data Fig. 2 [file 44318_2026_712_MOESM3_ESM.zip › Figure 2/2G/2b4_KO_Bleo_20x.tif]

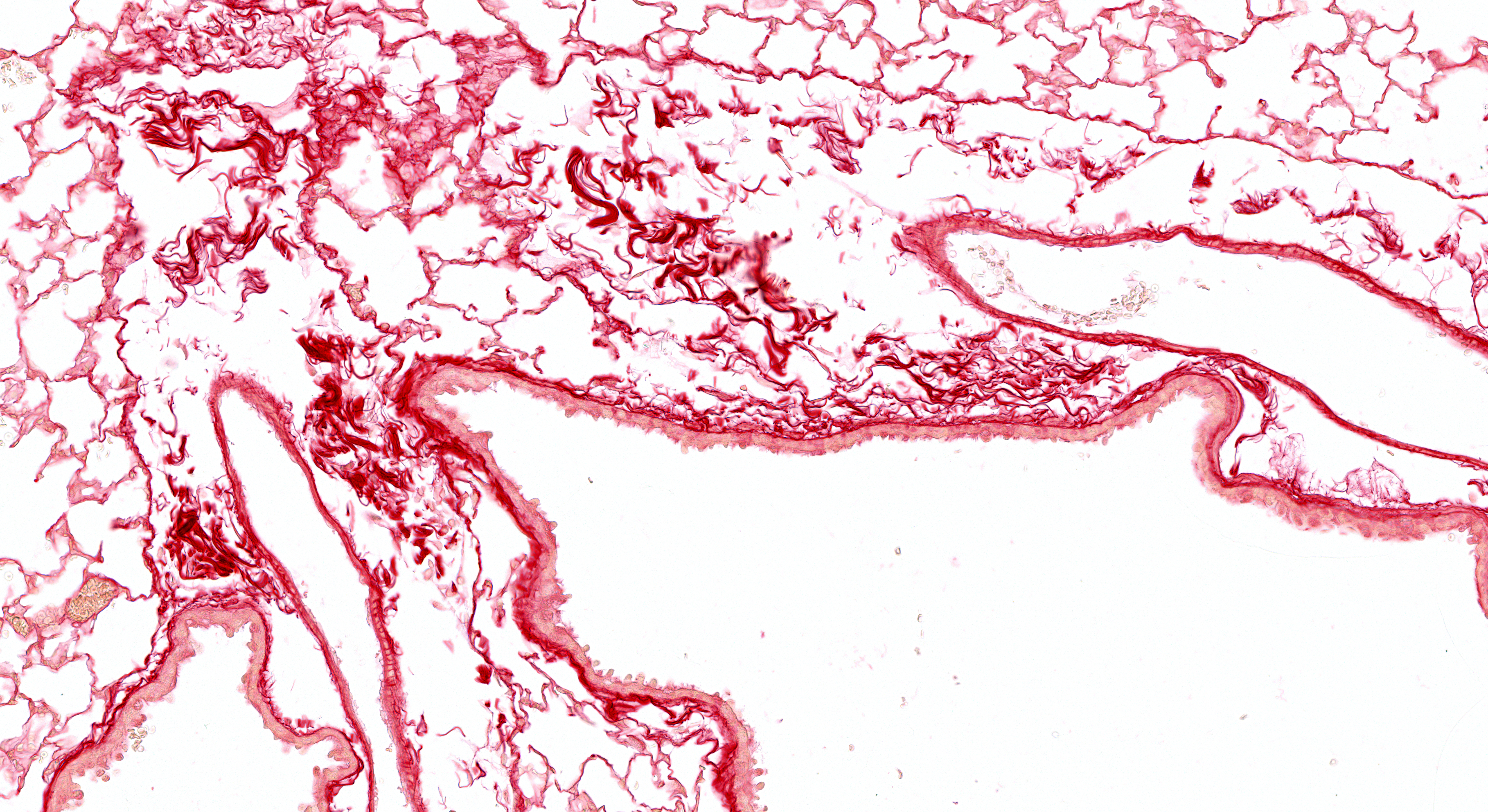

Supplement: Supplementary file 3 — Source data Fig. 2 [file 44318_2026_712_MOESM3_ESM.zip › Figure 2/2C/1a6_WT_PBS_20x4.tif]

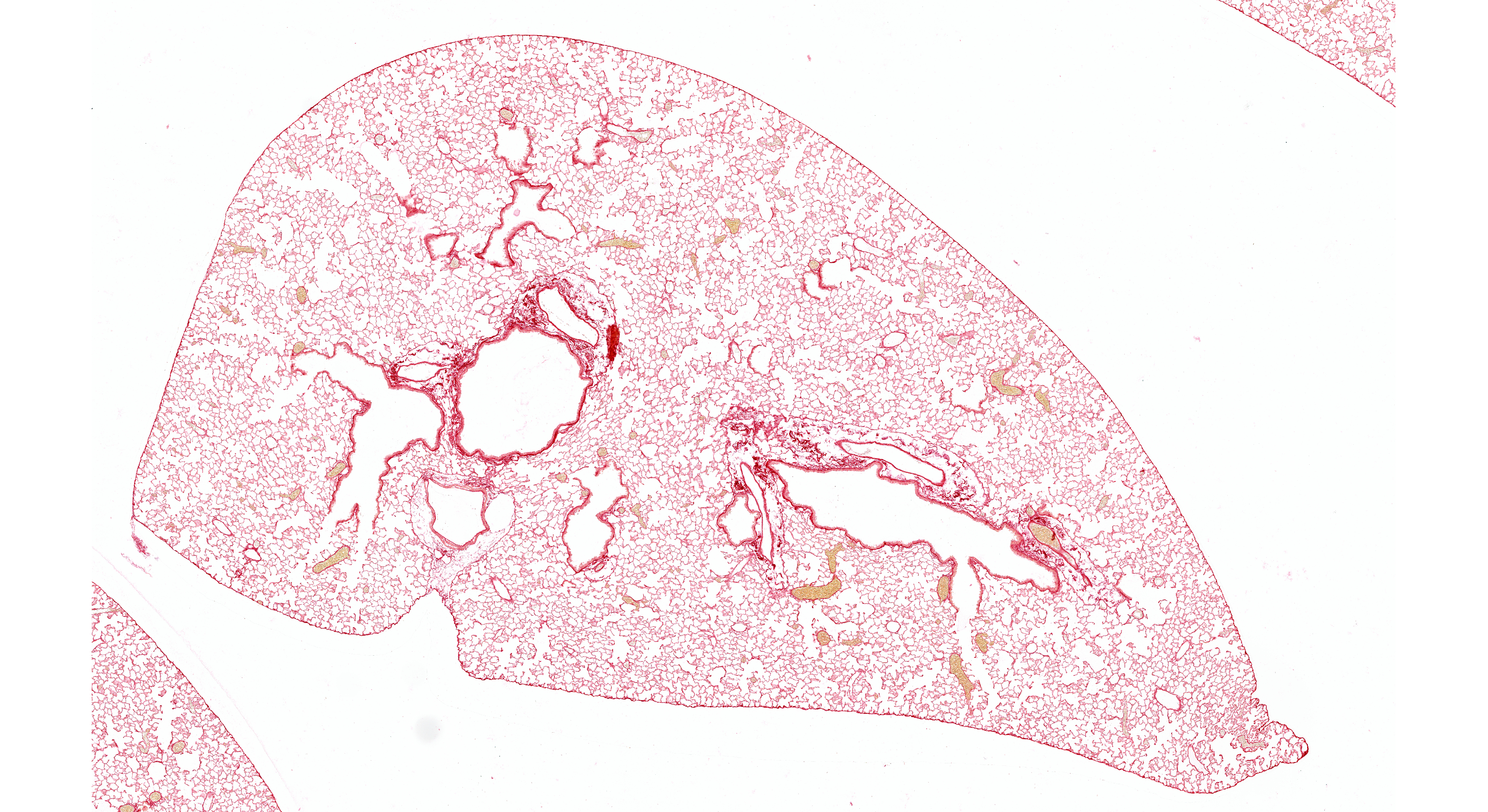

Supplement: Supplementary file 3 — Source data Fig. 2 [file 44318_2026_712_MOESM3_ESM.zip › Figure 2/2C/1a6_WT_PBS_Übersicht.tif]

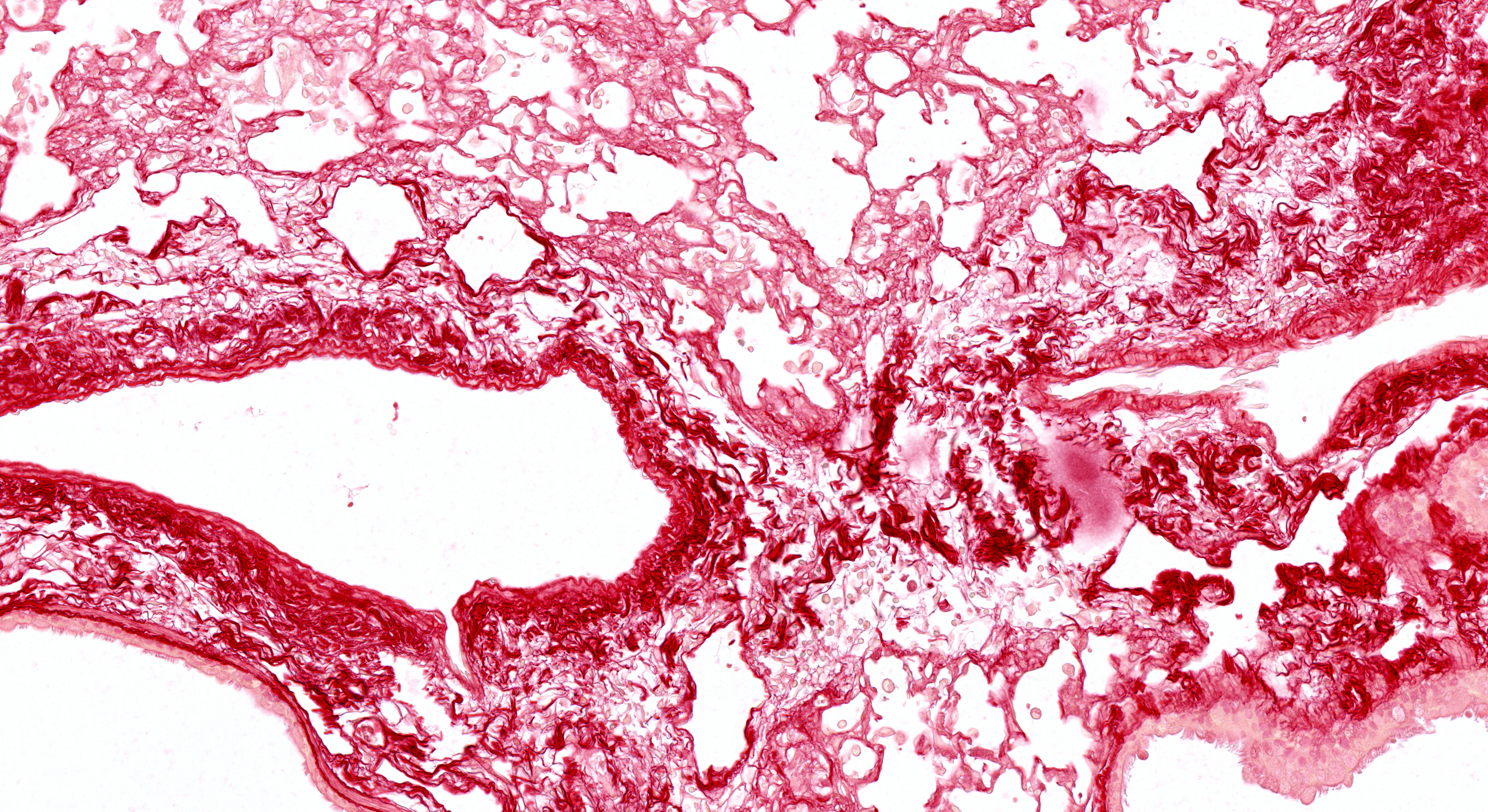

Supplement: Supplementary file 3 — Source data Fig. 2 [file 44318_2026_712_MOESM3_ESM.zip › Figure 2/2C/1b11_KO_Bleo_20x1.tif]

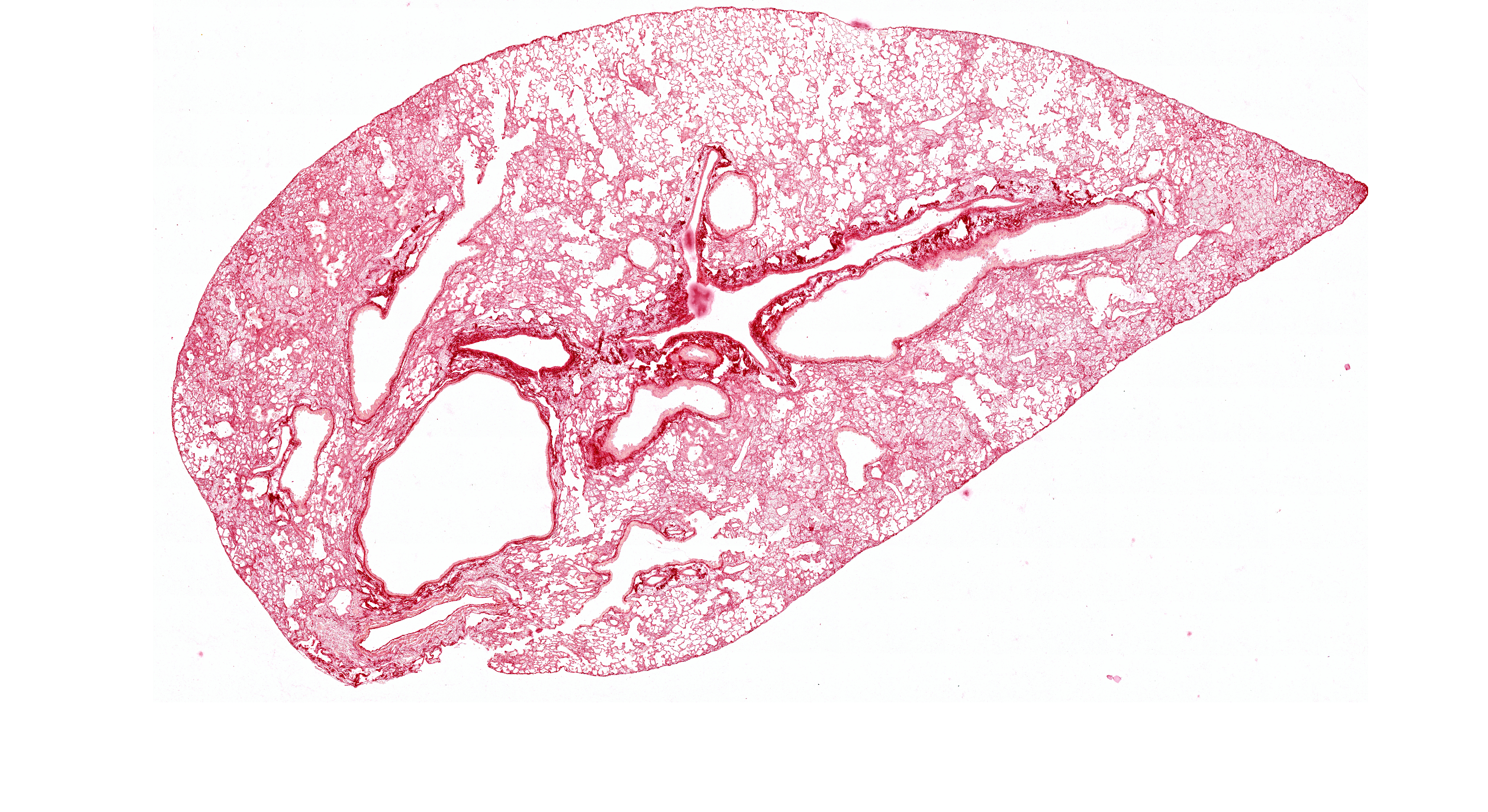

Supplement: Supplementary file 3 — Source data Fig. 2 [file 44318_2026_712_MOESM3_ESM.zip › Figure 2/2C/1b11_KO_Bleo_Übersicht.tif]

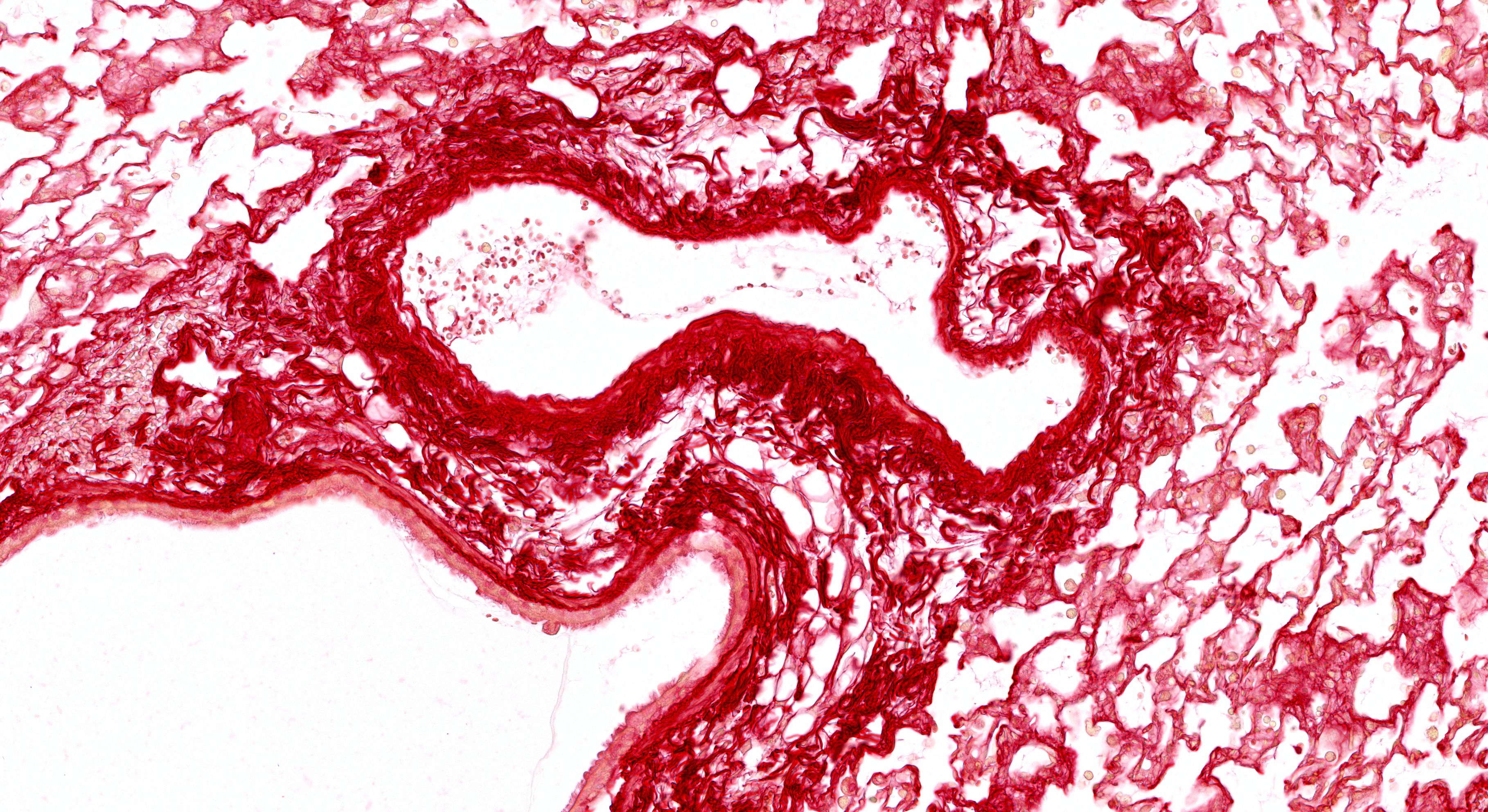

Supplement: Supplementary file 3 — Source data Fig. 2 [file 44318_2026_712_MOESM3_ESM.zip › Figure 2/2C/1b4_WT_Bleo_20x1.tif]

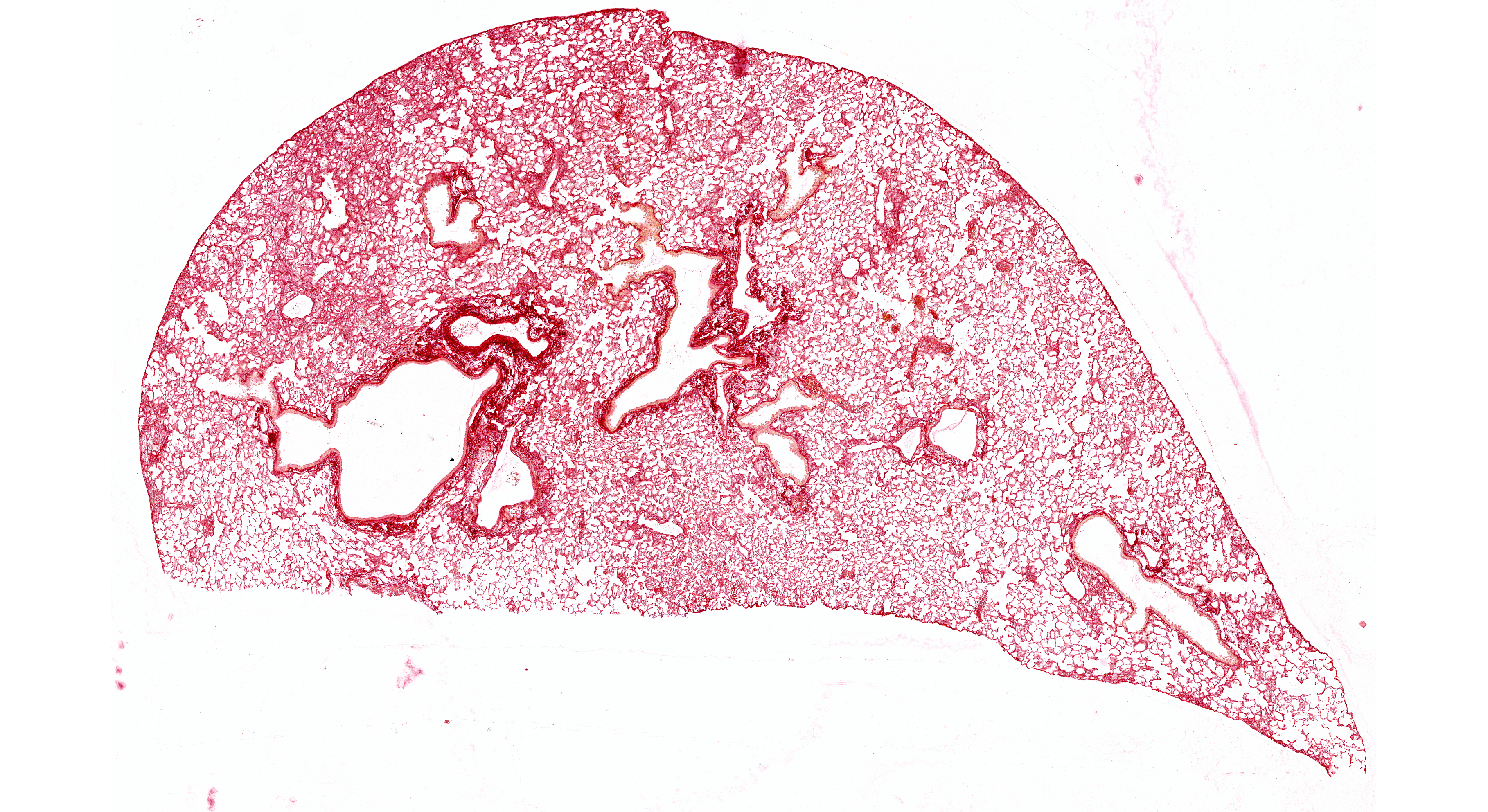

Supplement: Supplementary file 3 — Source data Fig. 2 [file 44318_2026_712_MOESM3_ESM.zip › Figure 2/2C/1b4_WT_Bleo_Übersicht.tif]

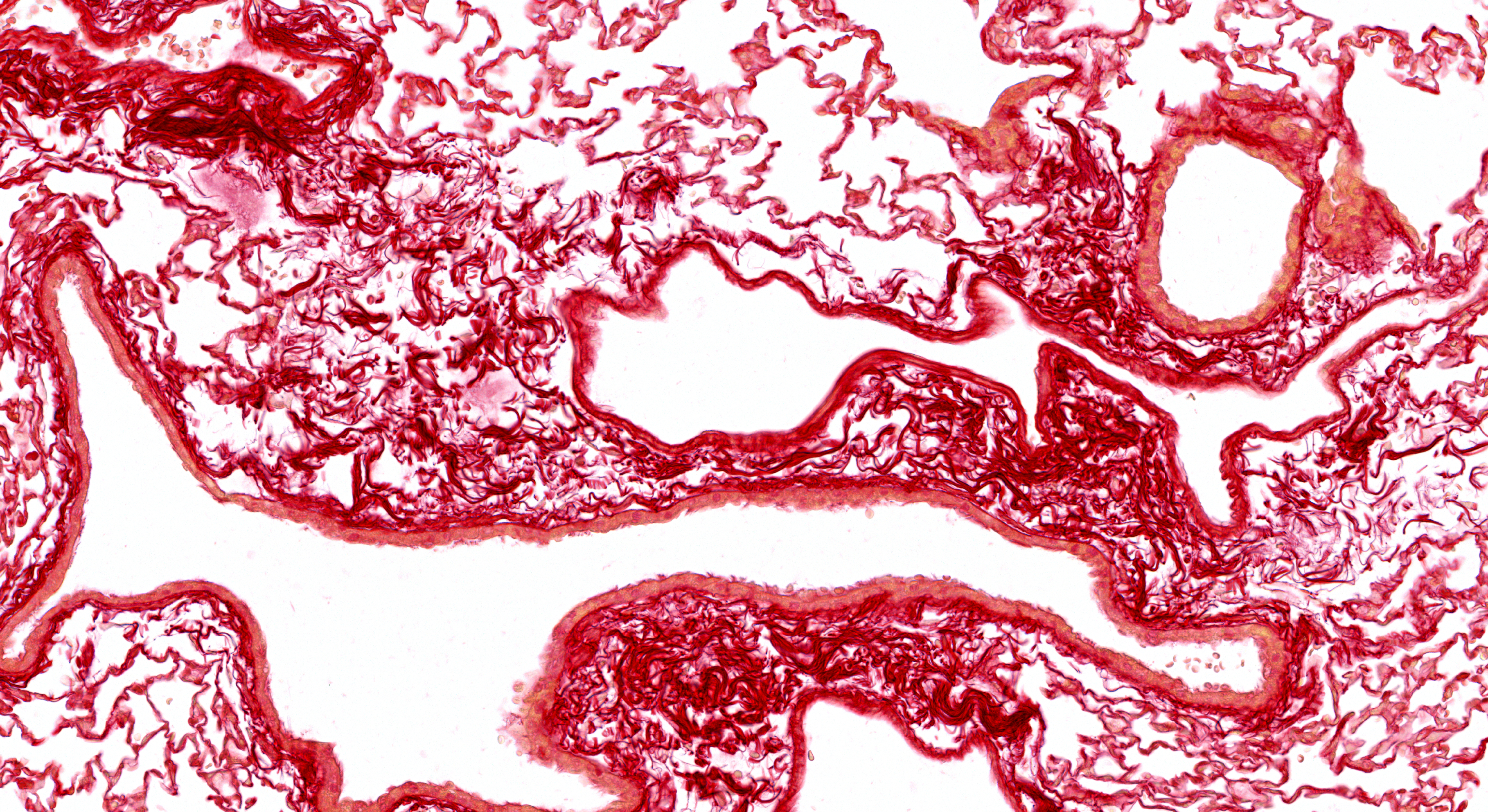

Supplement: Supplementary file 3 — Source data Fig. 2 [file 44318_2026_712_MOESM3_ESM.zip › Figure 2/2C/2a1_KO_PBS_20x2.tif]

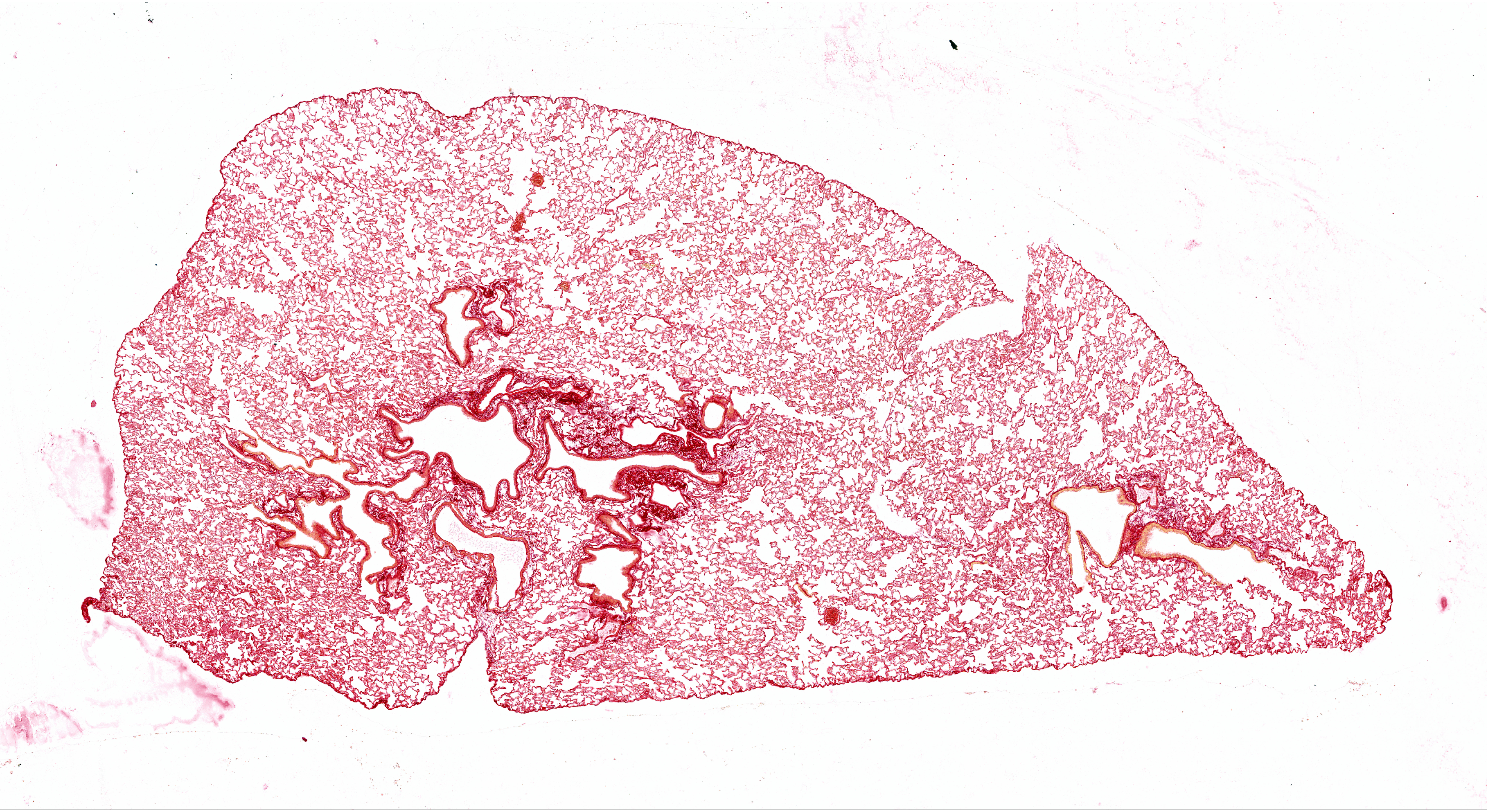

Supplement: Supplementary file 3 — Source data Fig. 2 [file 44318_2026_712_MOESM3_ESM.zip › Figure 2/2C/2a1_KO_PBS_Übersicht.tif]

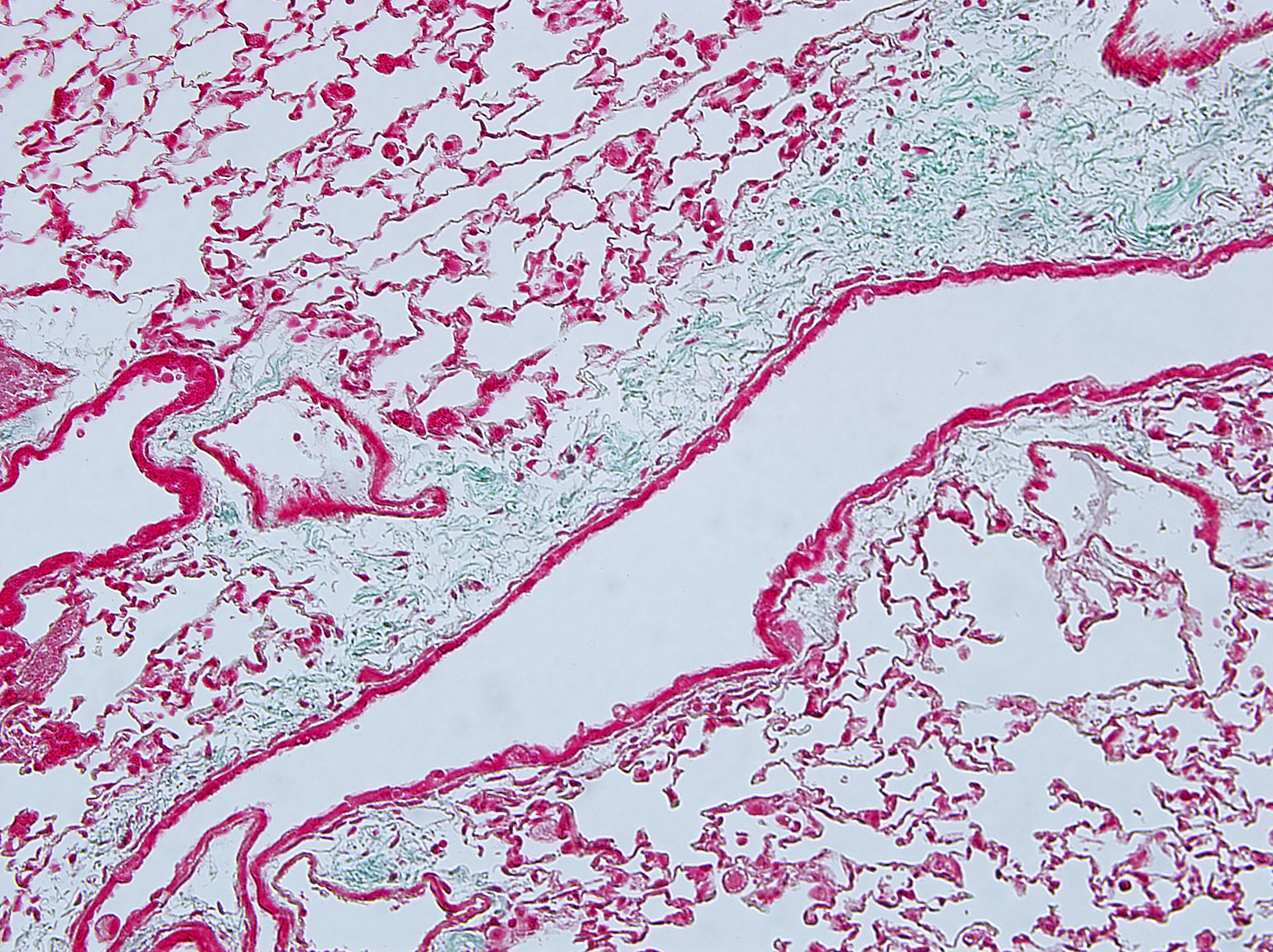

Supplement: Supplementary file 3 — Source data Fig. 2 [file 44318_2026_712_MOESM3_ESM.zip › Figure 2/2E/20x_KO Bleo.tif]

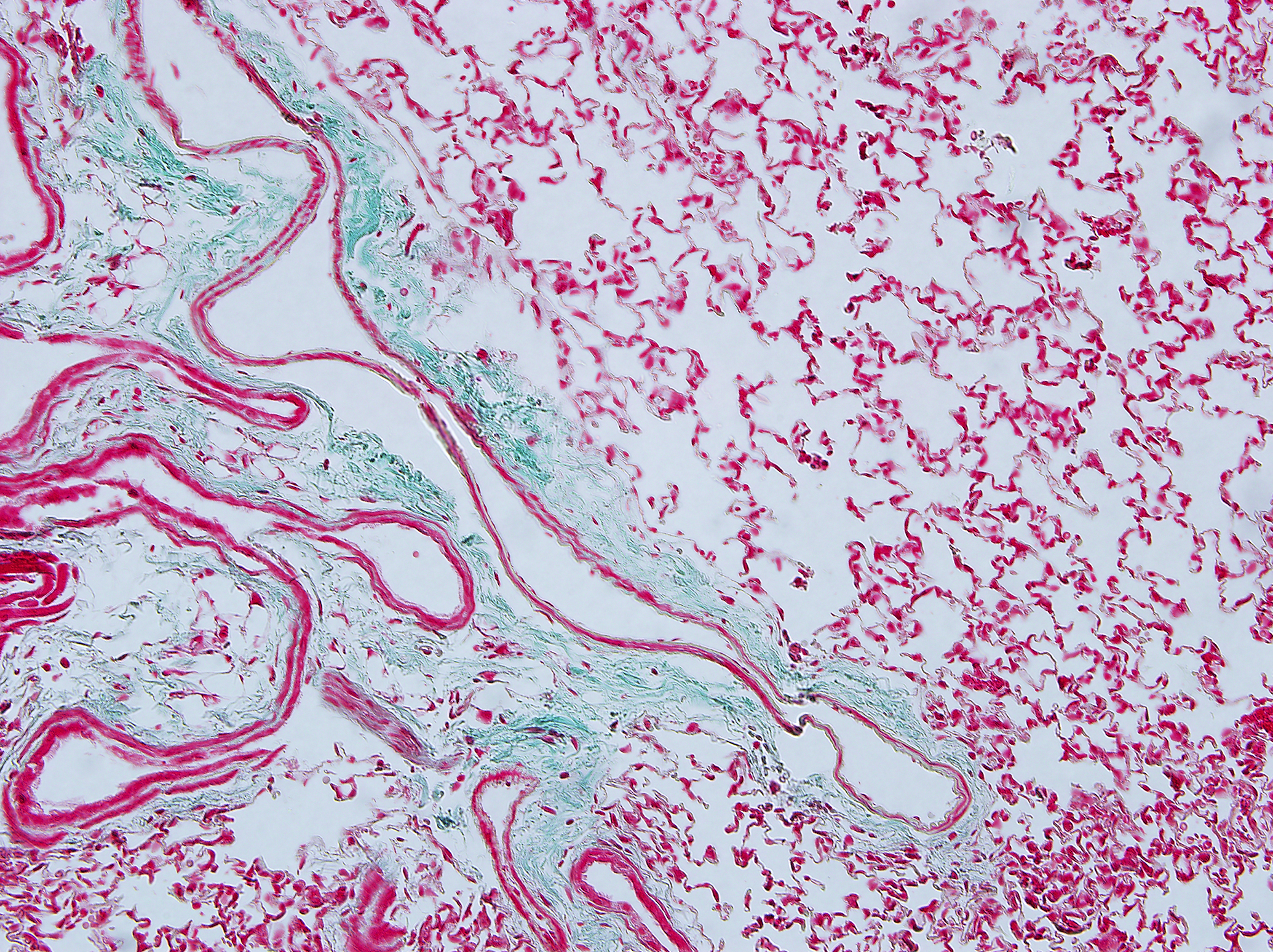

Supplement: Supplementary file 3 — Source data Fig. 2 [file 44318_2026_712_MOESM3_ESM.zip › Figure 2/2E/20x_KO PBS.tif]

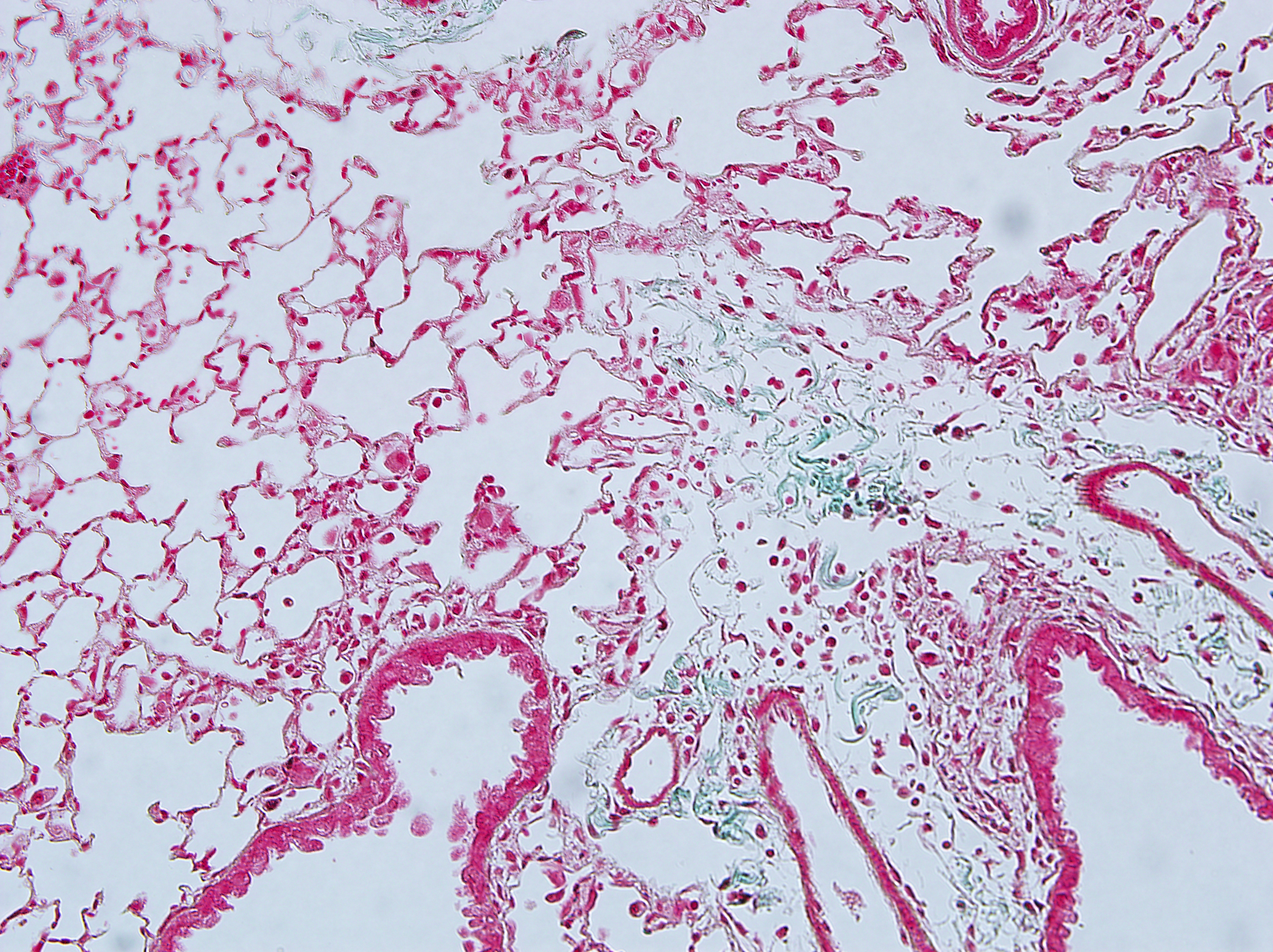

Supplement: Supplementary file 3 — Source data Fig. 2 [file 44318_2026_712_MOESM3_ESM.zip › Figure 2/2E/20x_WT Bleo.tif]

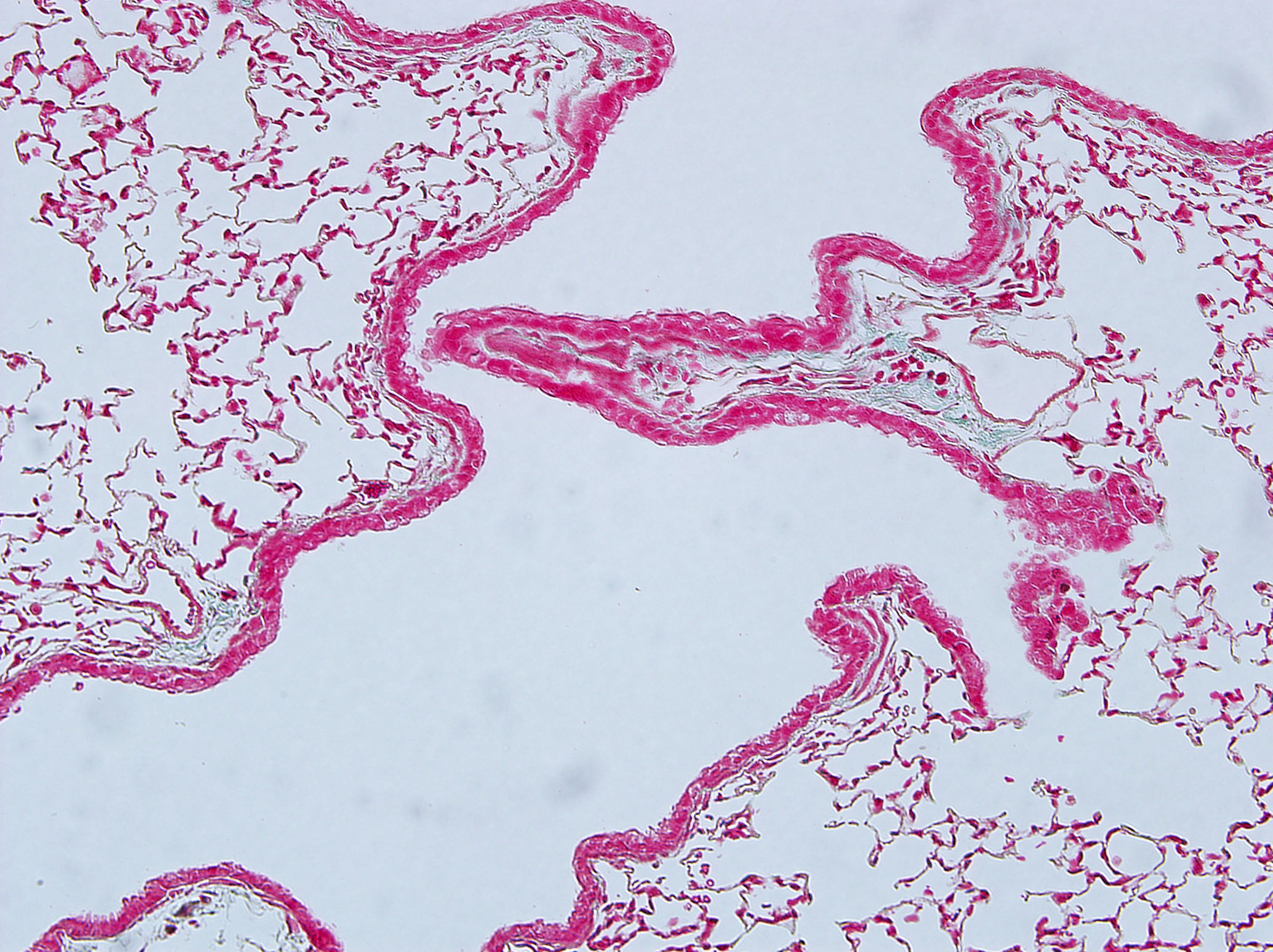

Supplement: Supplementary file 3 — Source data Fig. 2 [file 44318_2026_712_MOESM3_ESM.zip › Figure 2/2E/20x_WT PBS.tif]

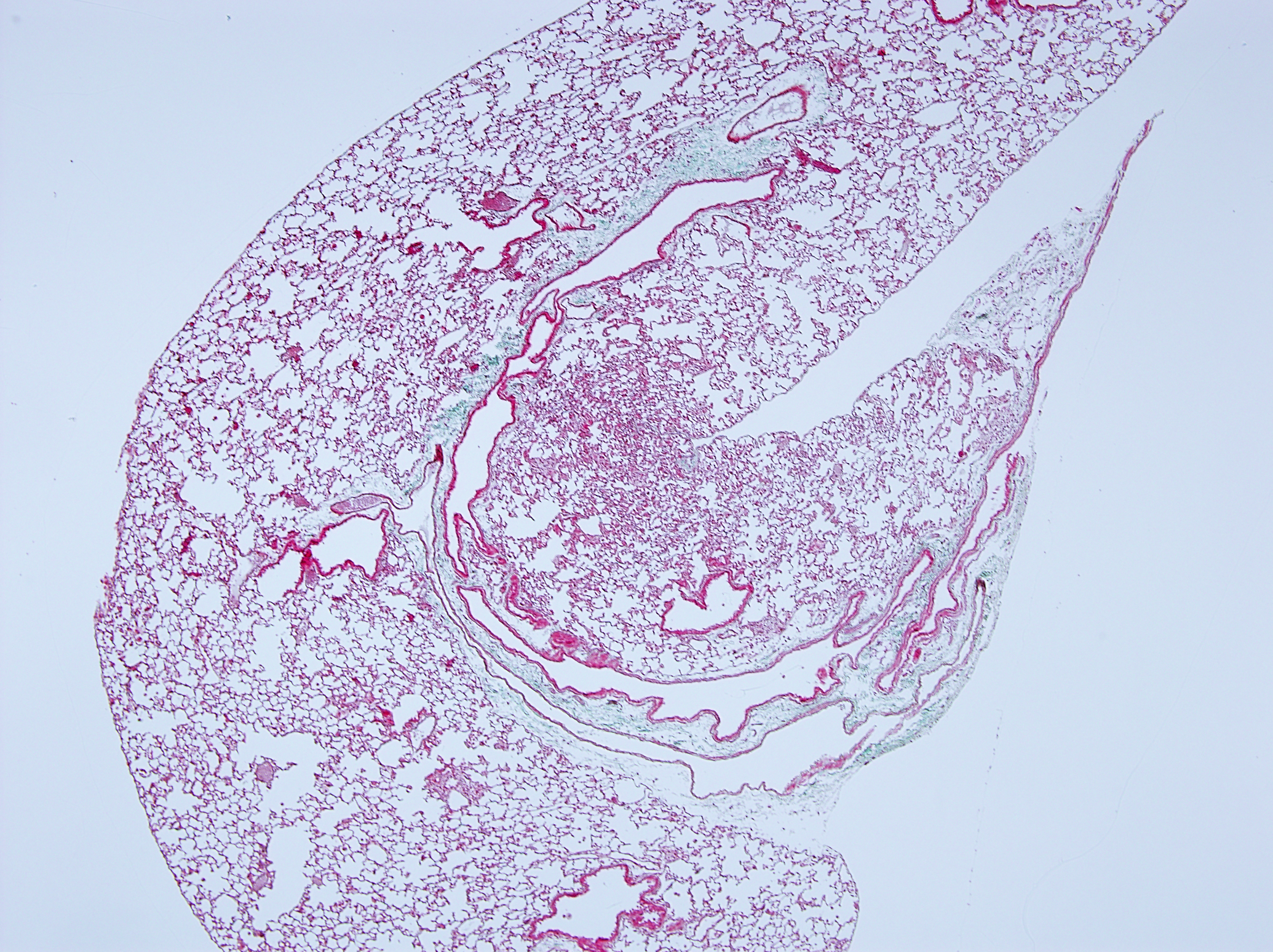

Supplement: Supplementary file 3 — Source data Fig. 2 [file 44318_2026_712_MOESM3_ESM.zip › Figure 2/2E/4x_KO Bleo.tif]

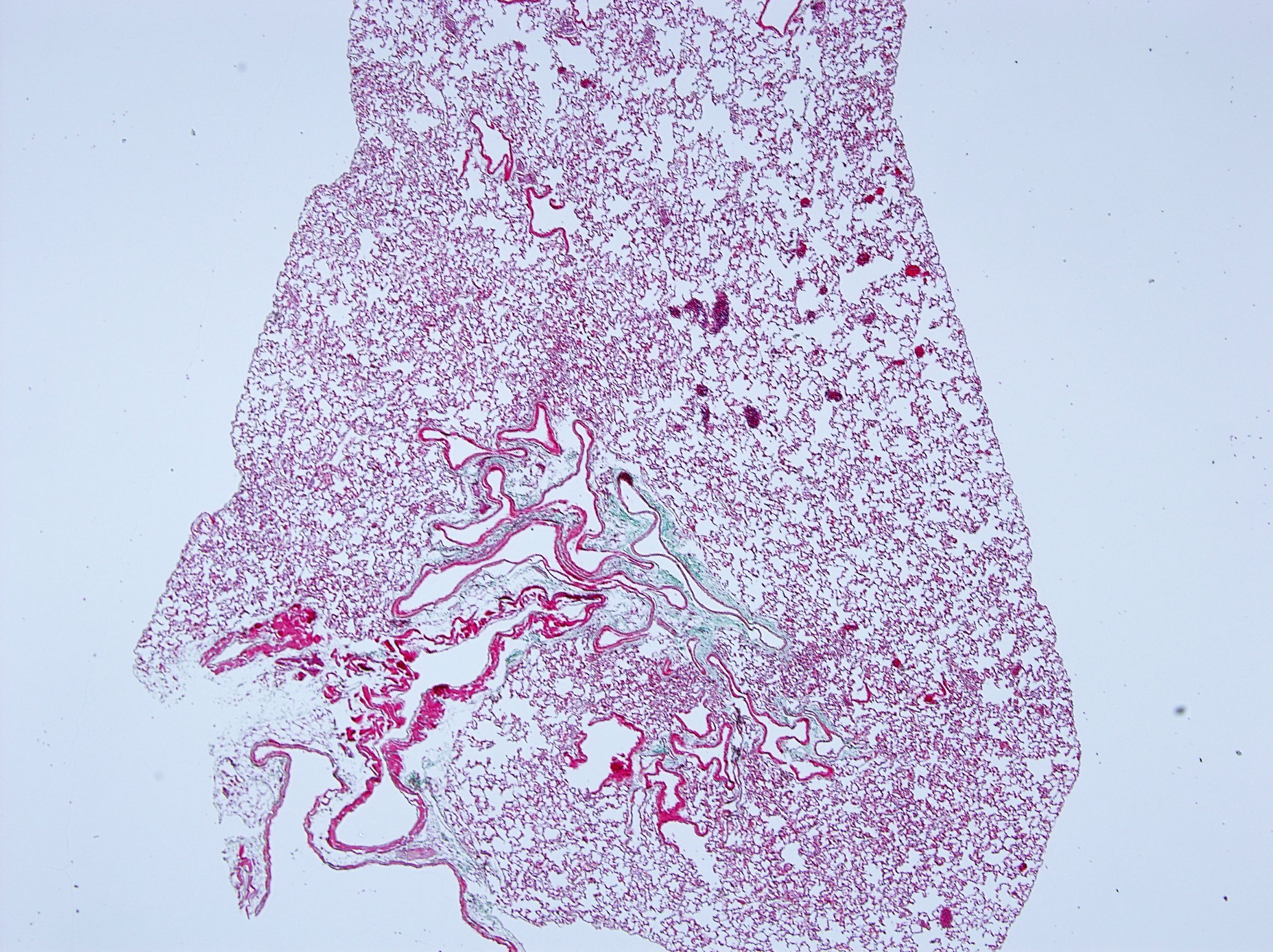

Supplement: Supplementary file 3 — Source data Fig. 2 [file 44318_2026_712_MOESM3_ESM.zip › Figure 2/2E/4x_KO PBS.tif]

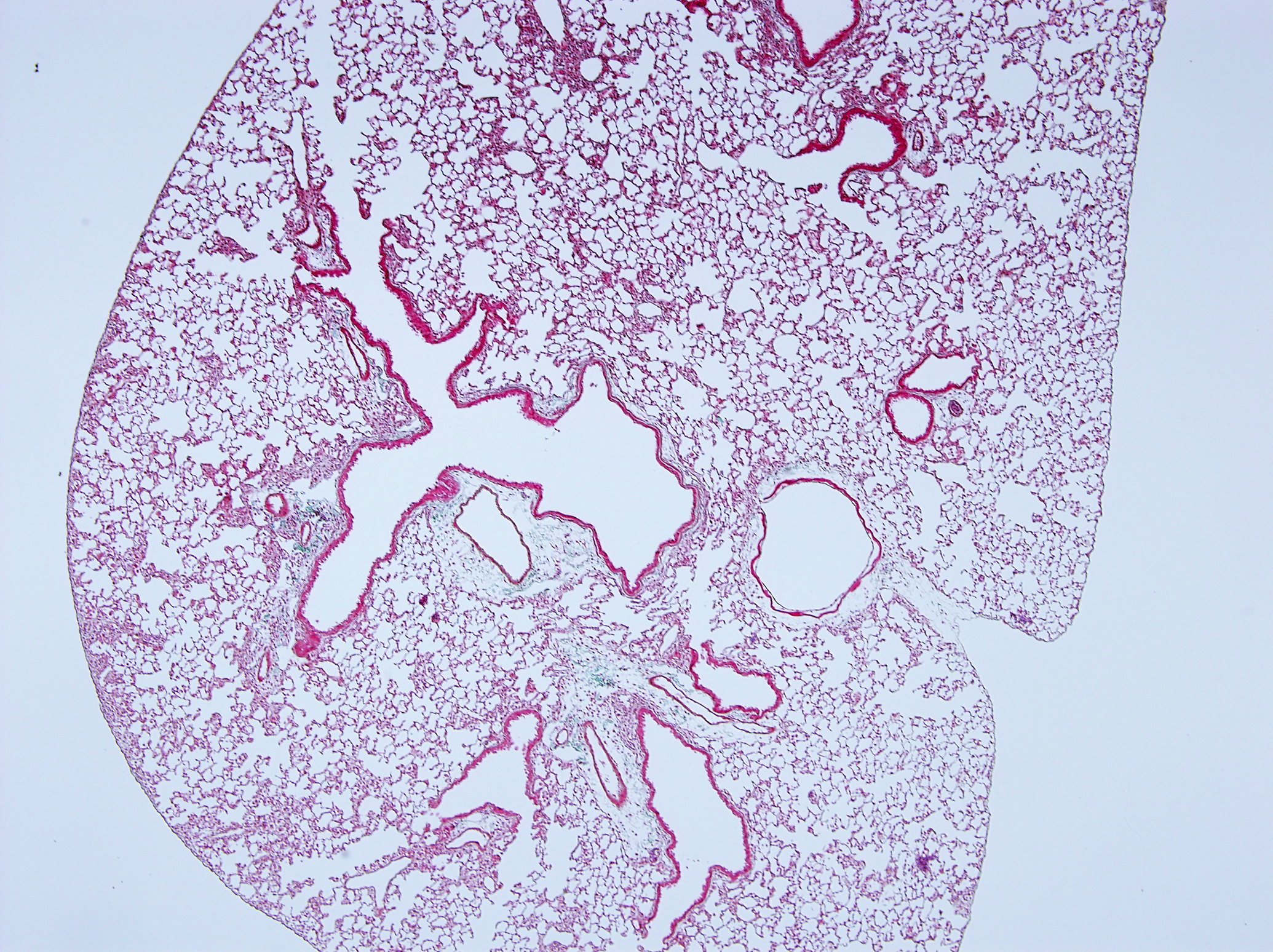

Supplement: Supplementary file 3 — Source data Fig. 2 [file 44318_2026_712_MOESM3_ESM.zip › Figure 2/2E/4x_WT Bleo.tif]

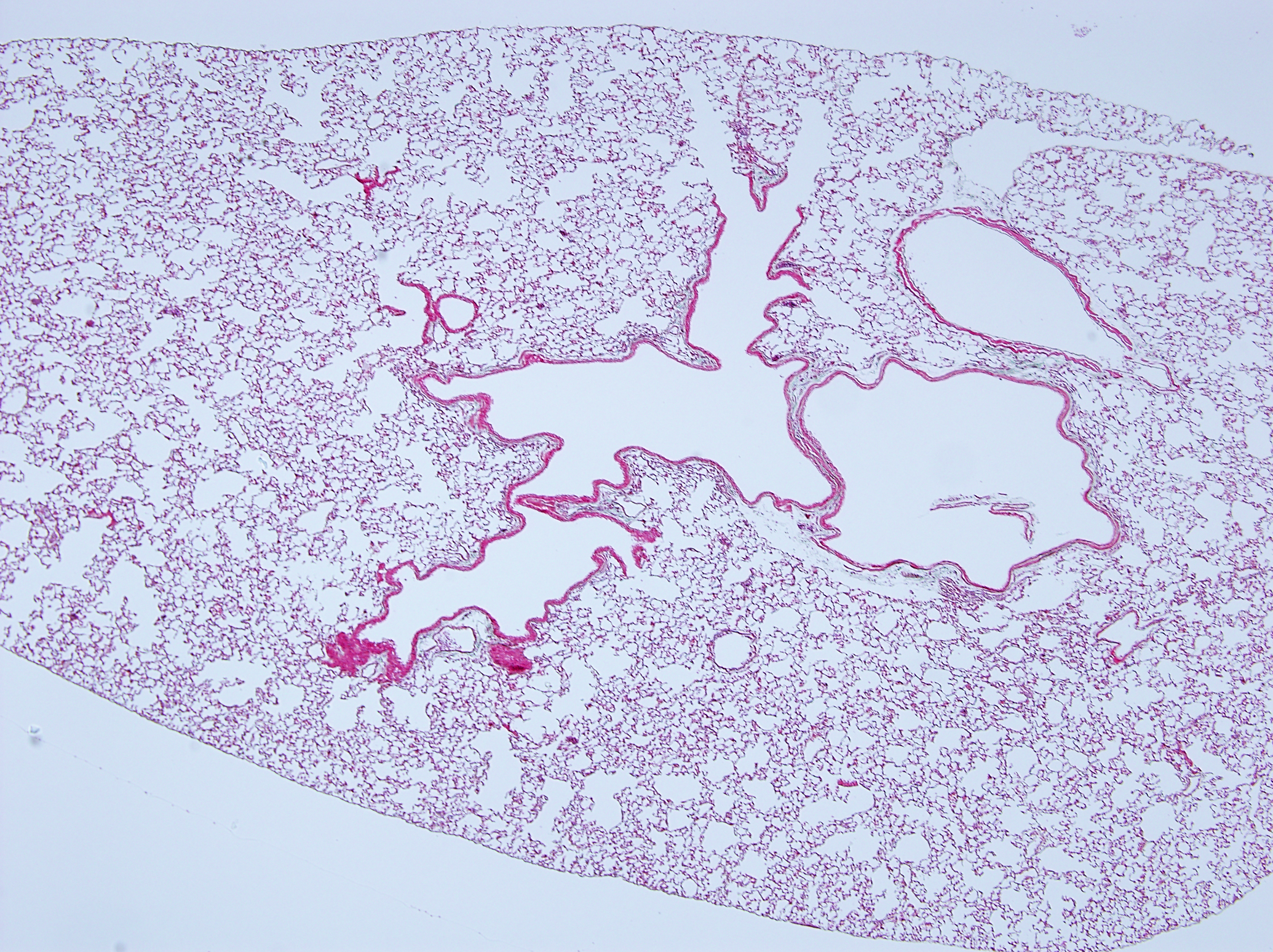

Supplement: Supplementary file 3 — Source data Fig. 2 [file 44318_2026_712_MOESM3_ESM.zip › Figure 2/2E/4x_WT PBS.tif]

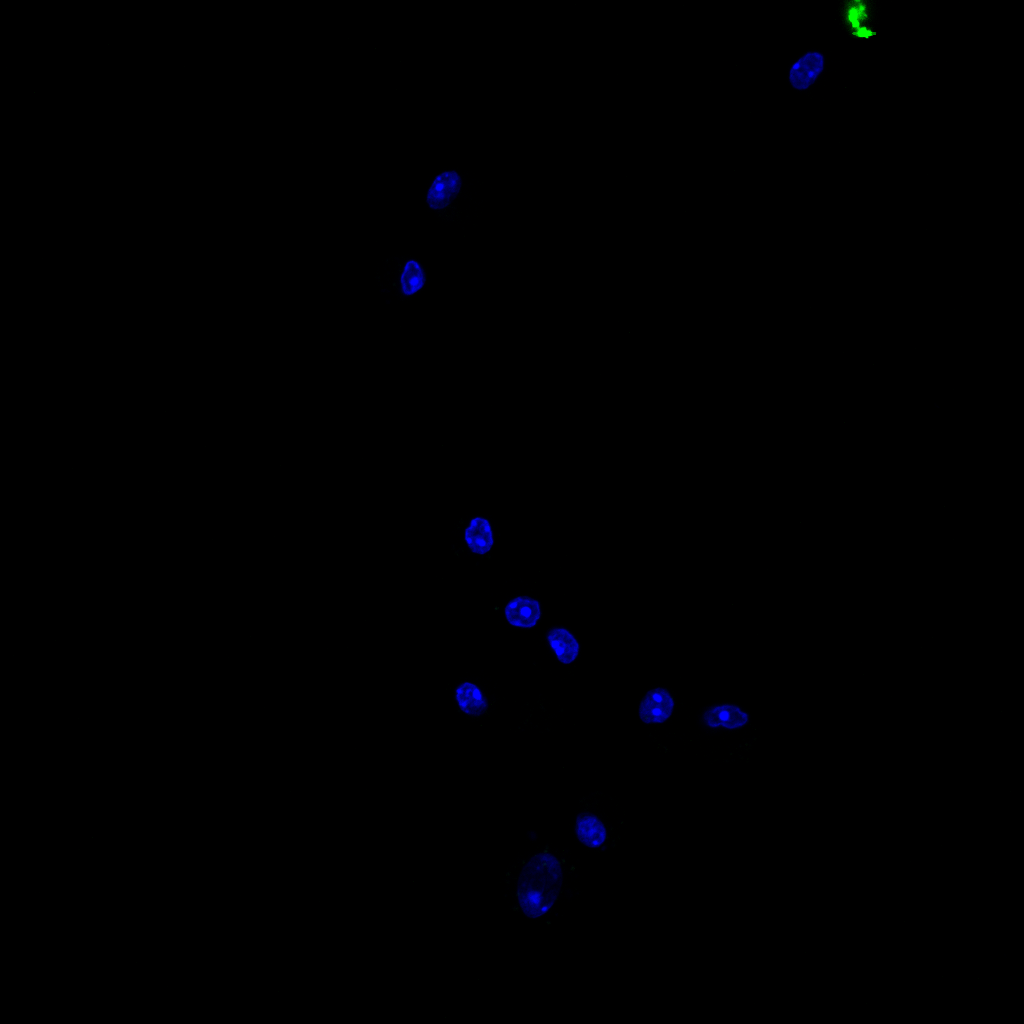

Supplement: Supplementary file 7 — Source data Fig. 6 [file 44318_2026_712_MOESM7_ESM.zip › Figure 6/6F-G/Fig6 G - Confocal Images/TRPML1KO Alveolar Macrophages/DMSO/Composite (RGB).tif]

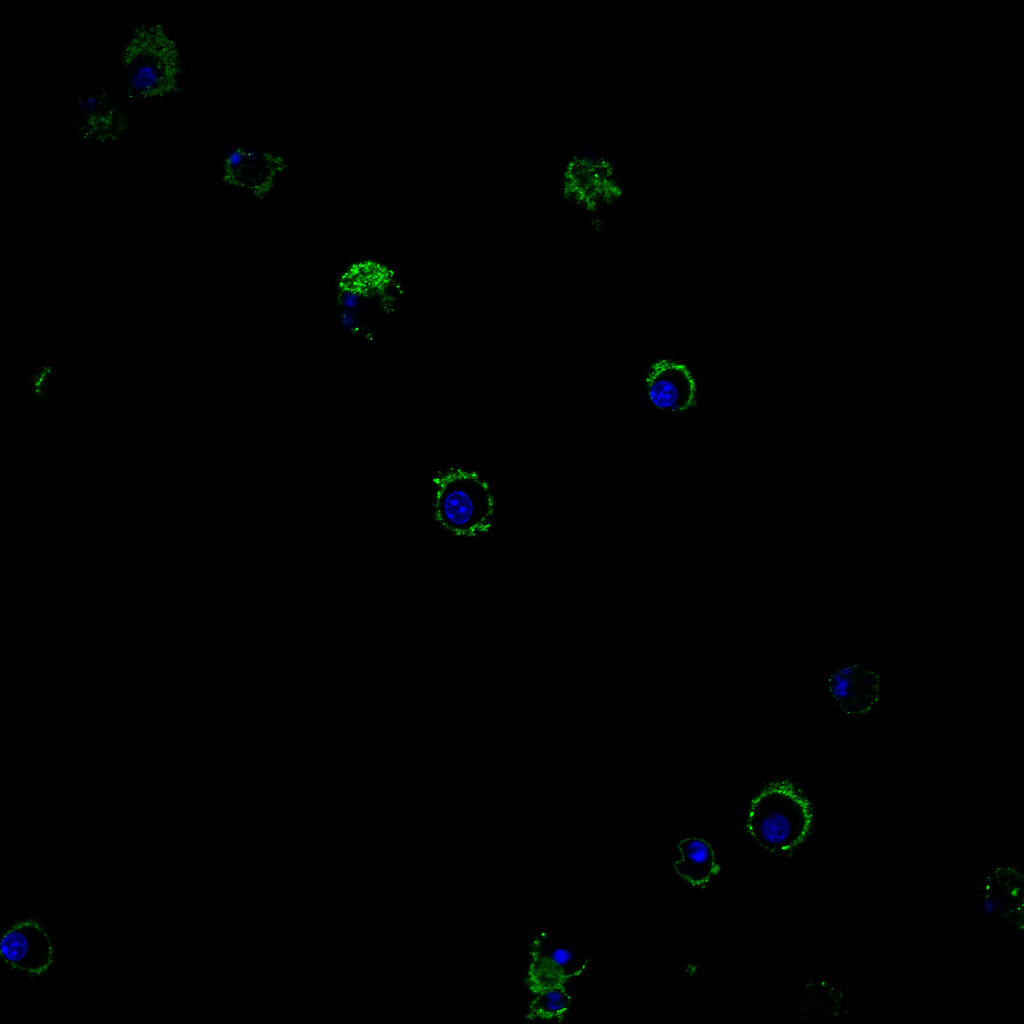

Supplement: Supplementary file 7 — Source data Fig. 6 [file 44318_2026_712_MOESM7_ESM.zip › Figure 6/6F-G/Fig6 G - Confocal Images/TRPML1KO Alveolar Macrophages/Ionomycin/TRPML1 KO_4uM Iono 10min 1.tif]

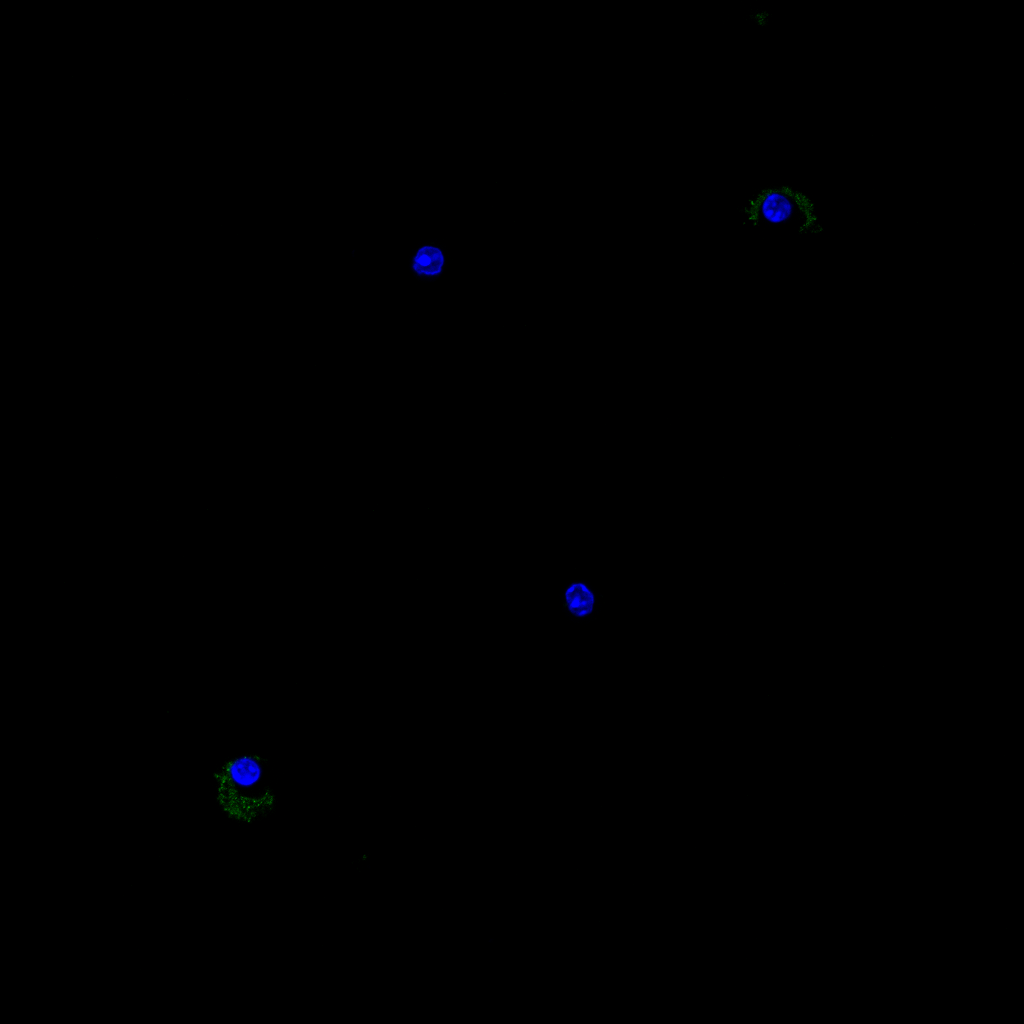

Supplement: Supplementary file 7 — Source data Fig. 6 [file 44318_2026_712_MOESM7_ESM.zip › Figure 6/6F-G/Fig6 G - Confocal Images/TRPML1KO Alveolar Macrophages/MLSA1/Composite (RGB).tif]

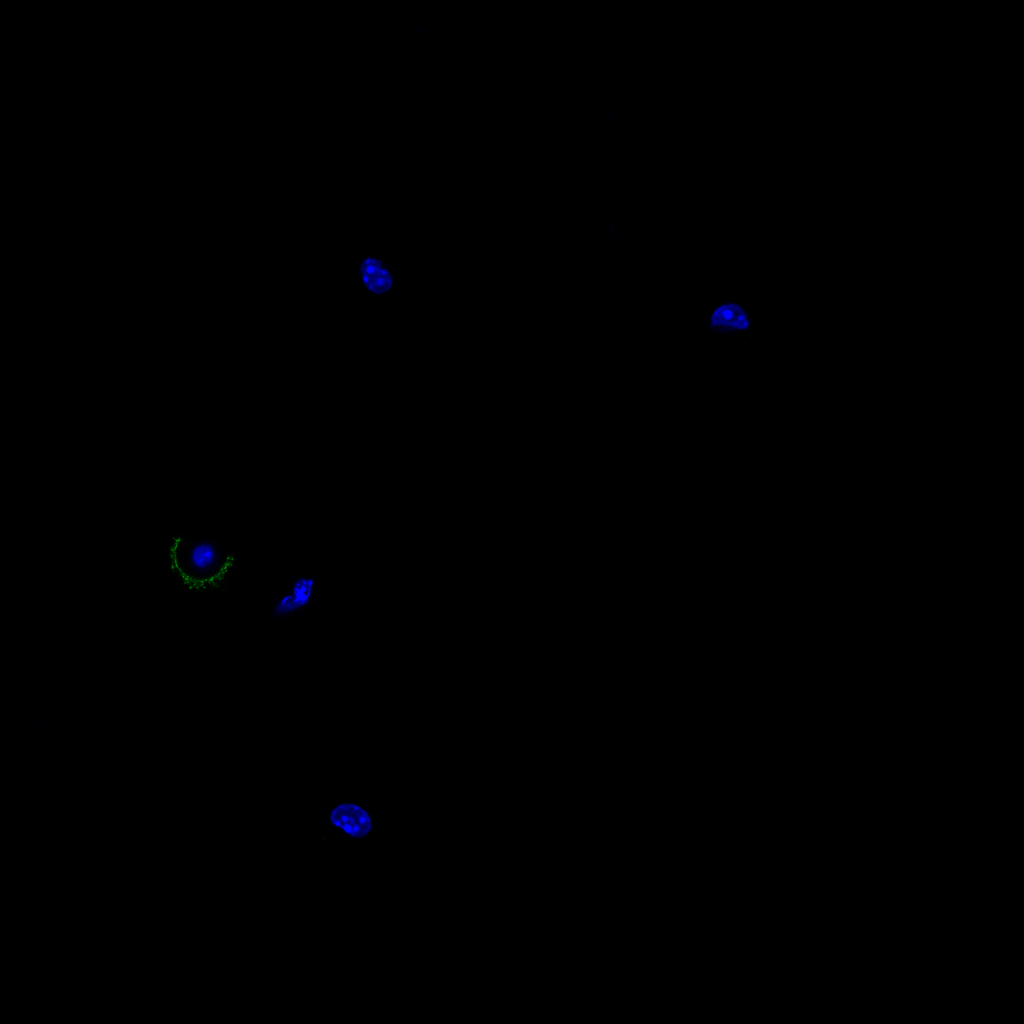

Supplement: Supplementary file 7 — Source data Fig. 6 [file 44318_2026_712_MOESM7_ESM.zip › Figure 6/6F-G/Fig6 G - Confocal Images/TRPML1KO Alveolar Macrophages/WR1-002/Composite (RGB).tif]

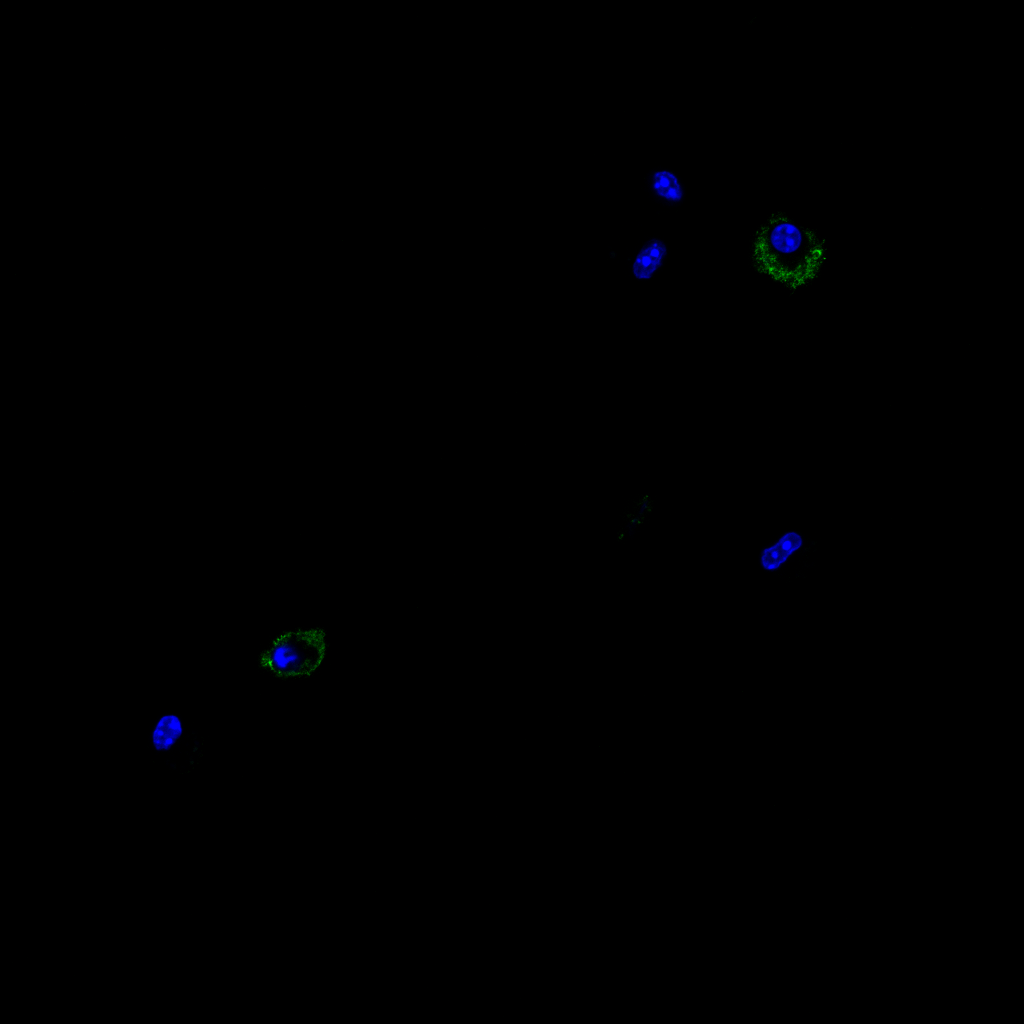

Supplement: Supplementary file 7 — Source data Fig. 6 [file 44318_2026_712_MOESM7_ESM.zip › Figure 6/6F-G/Fig6 G - Confocal Images/TRPML1KO Alveolar Macrophages/WR250-003/Composite (RGB).tif]

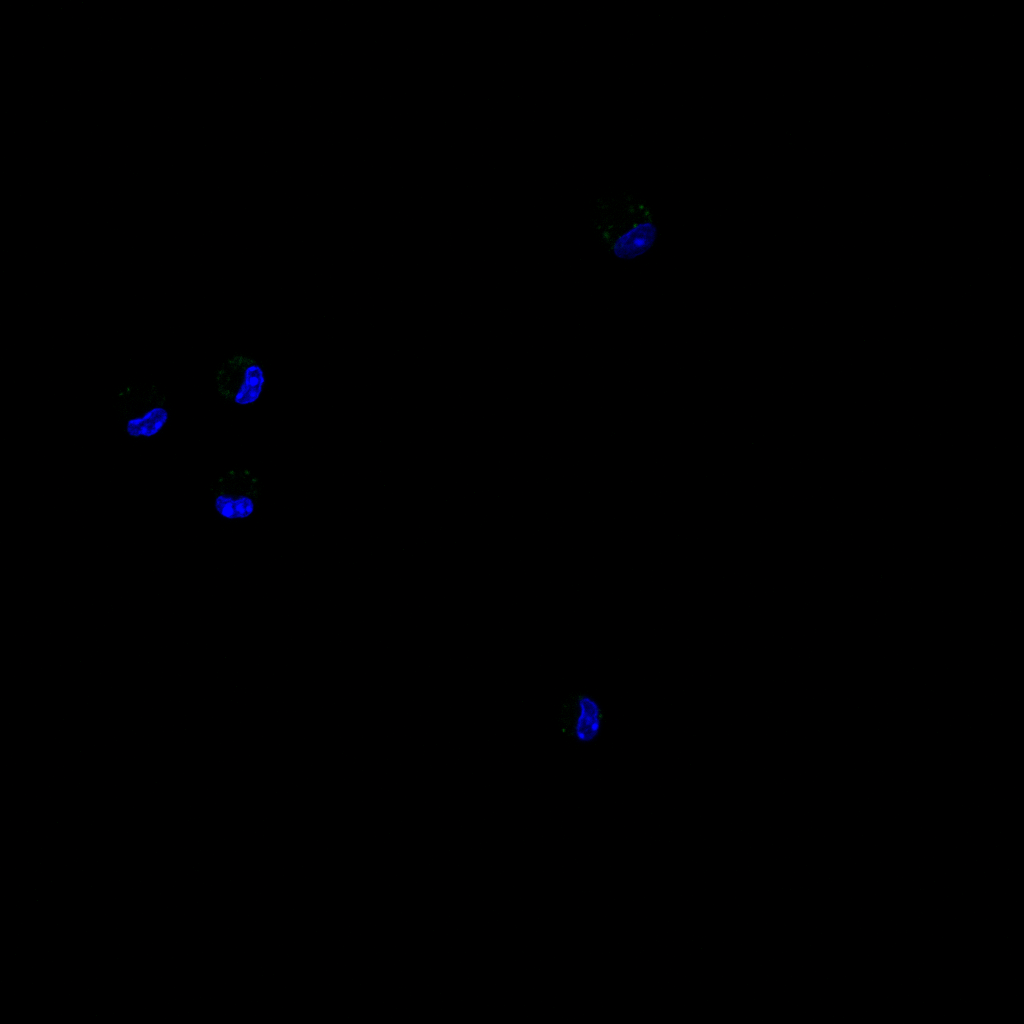

Supplement: Supplementary file 7 — Source data Fig. 6 [file 44318_2026_712_MOESM7_ESM.zip › Figure 6/6F-G/Fig6 G - Confocal Images/WT Alveolar macrophages/DMSO/Composite (RGB).tif]

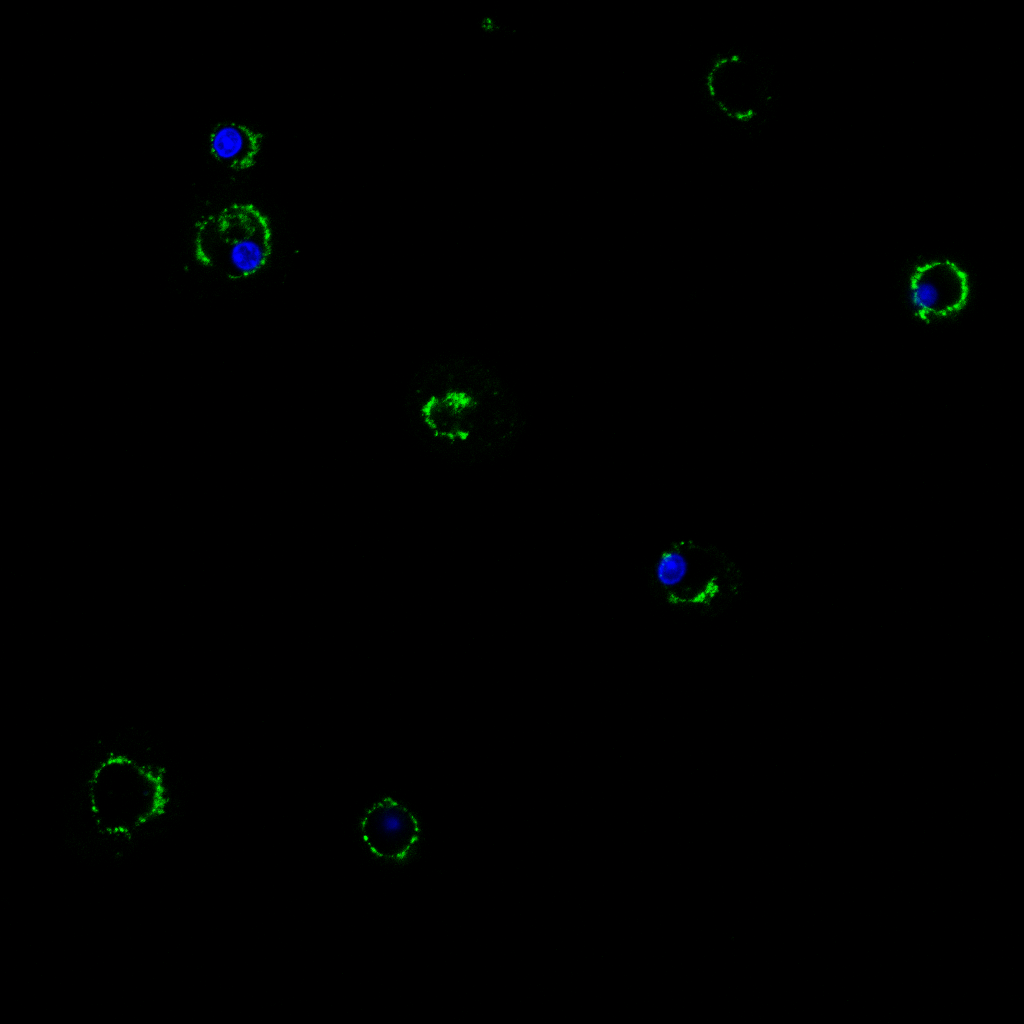

Supplement: Supplementary file 7 — Source data Fig. 6 [file 44318_2026_712_MOESM7_ESM.zip › Figure 6/6F-G/Fig6 G - Confocal Images/WT Alveolar macrophages/Ionomycin/Composite (RGB).tif]

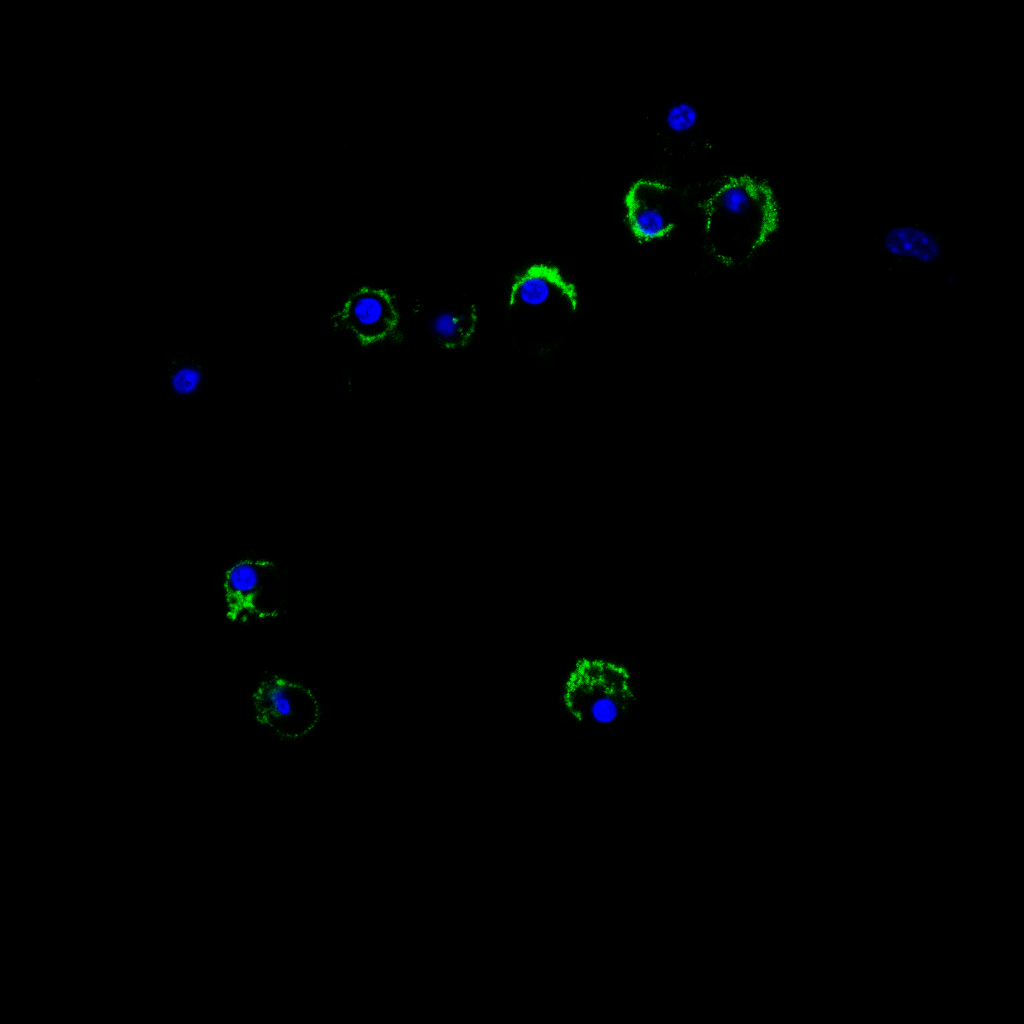

Supplement: Supplementary file 7 — Source data Fig. 6 [file 44318_2026_712_MOESM7_ESM.zip › Figure 6/6F-G/Fig6 G - Confocal Images/WT Alveolar macrophages/MLSA1/Composite (RGB).tif]

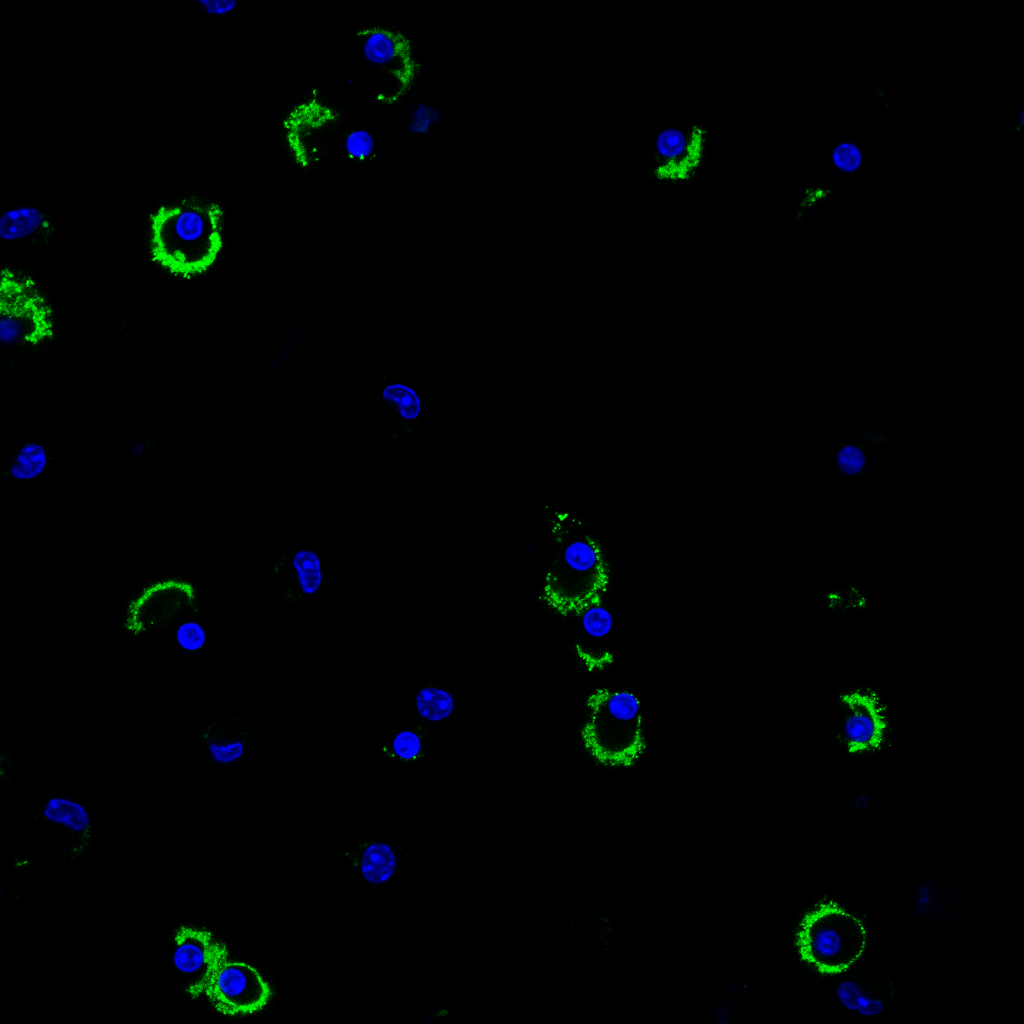

Supplement: Supplementary file 7 — Source data Fig. 6 [file 44318_2026_712_MOESM7_ESM.zip › Figure 6/6F-G/Fig6 G - Confocal Images/WT Alveolar macrophages/WR1-002/Composite (RGB).tif]

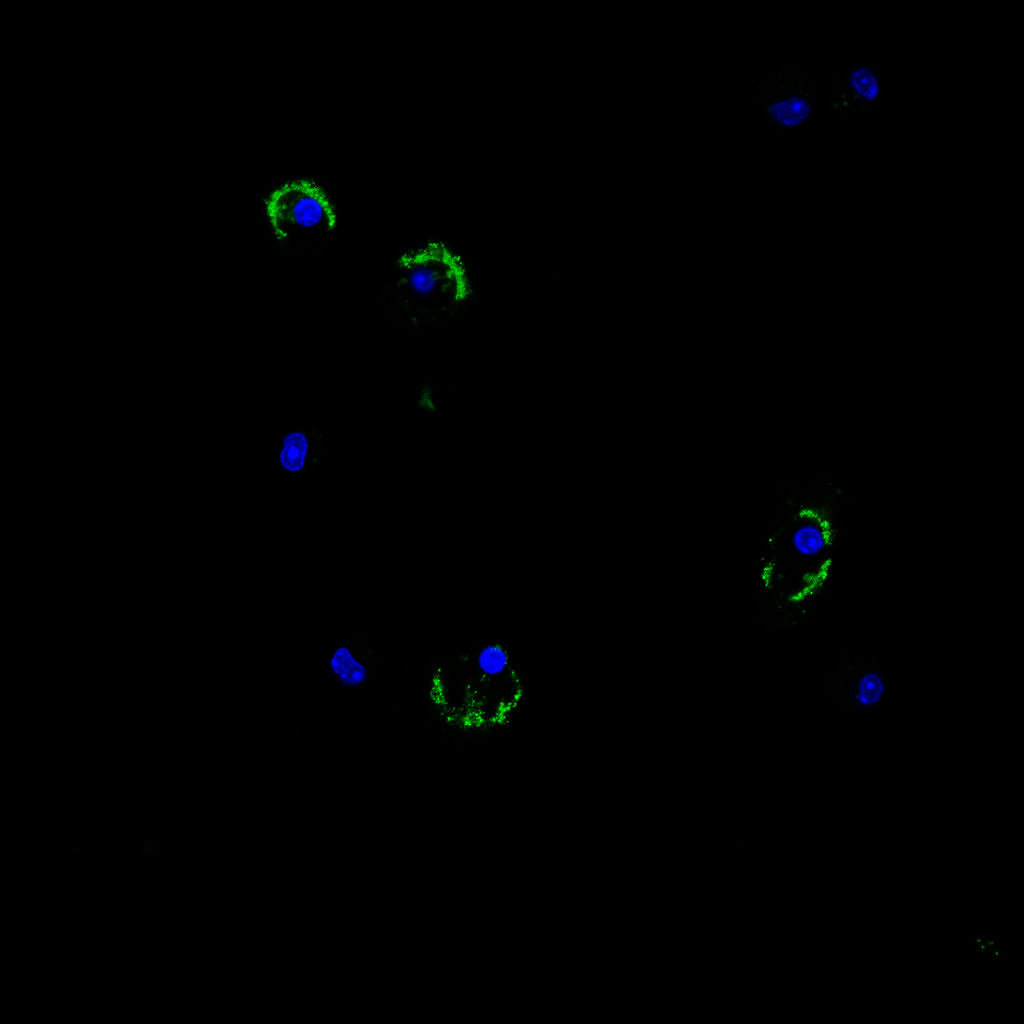

Supplement: Supplementary file 7 — Source data Fig. 6 [file 44318_2026_712_MOESM7_ESM.zip › Figure 6/6F-G/Fig6 G - Confocal Images/WT Alveolar macrophages/WR250-003/Composite (RGB).tif]

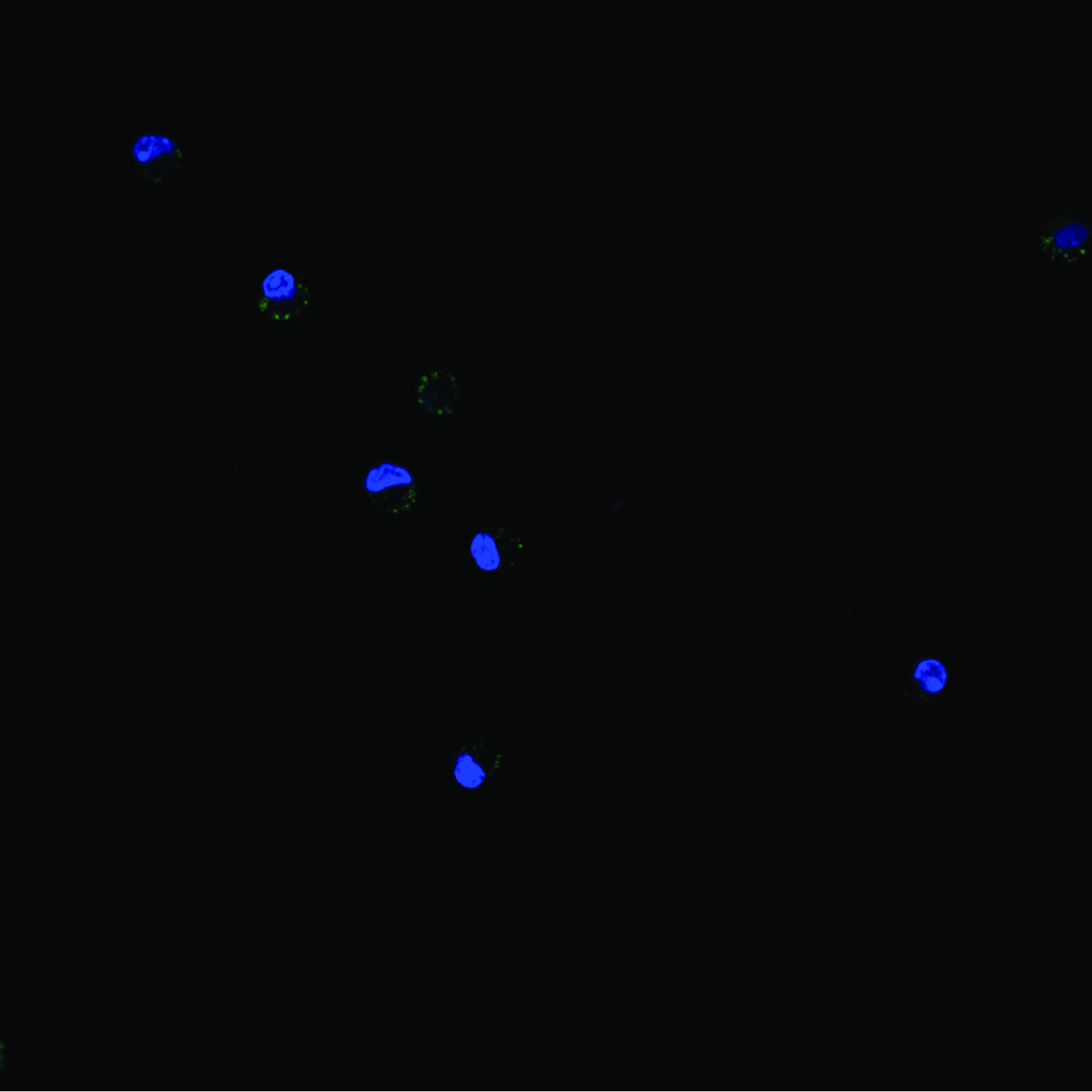

Supplement: Supplementary file 7 — Source data Fig. 6 [file 44318_2026_712_MOESM7_ESM.zip › Figure 6/6D/WTDMSO.tif]

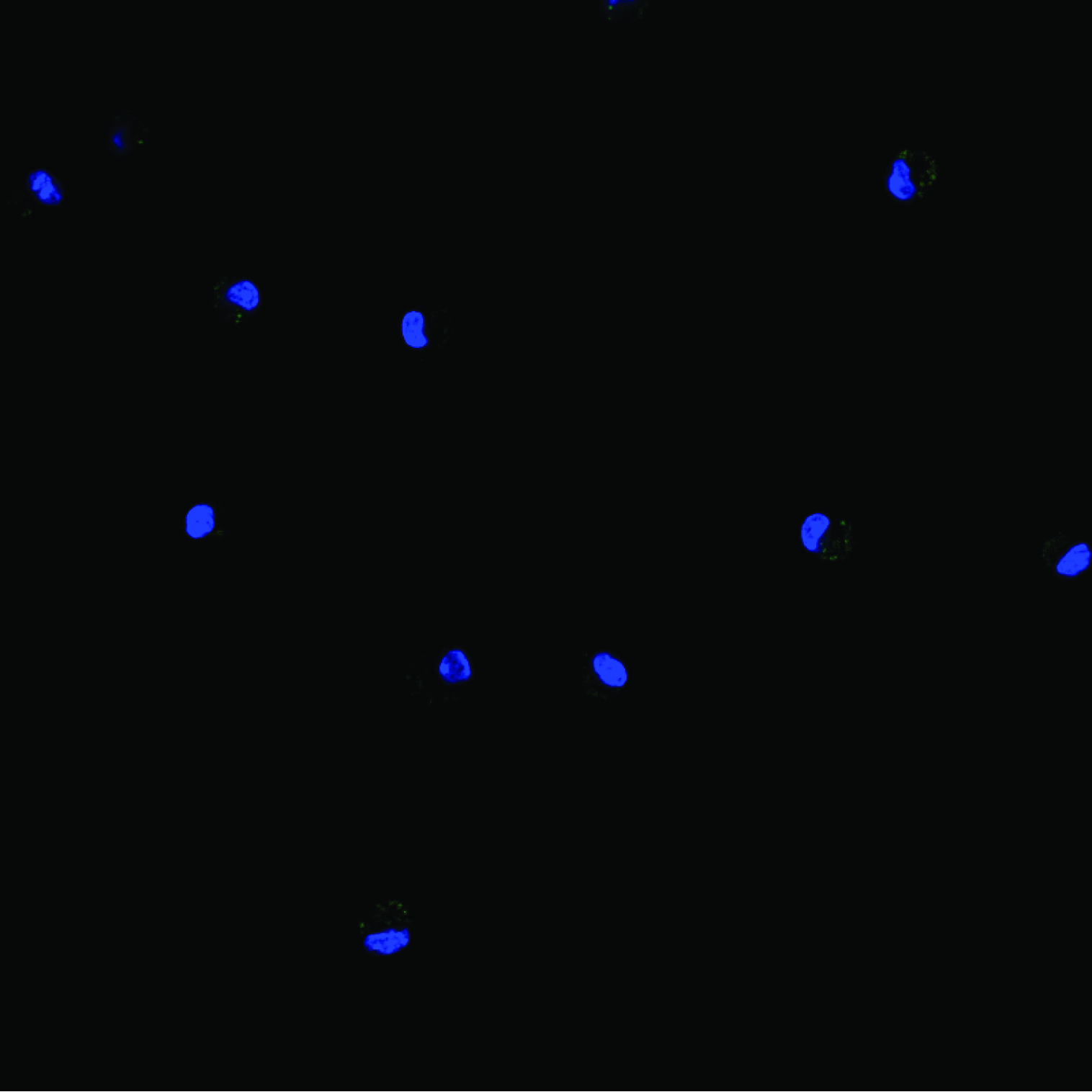

Supplement: Supplementary file 7 — Source data Fig. 6 [file 44318_2026_712_MOESM7_ESM.zip › Figure 6/6D/WTDMSO+EDME.tif]

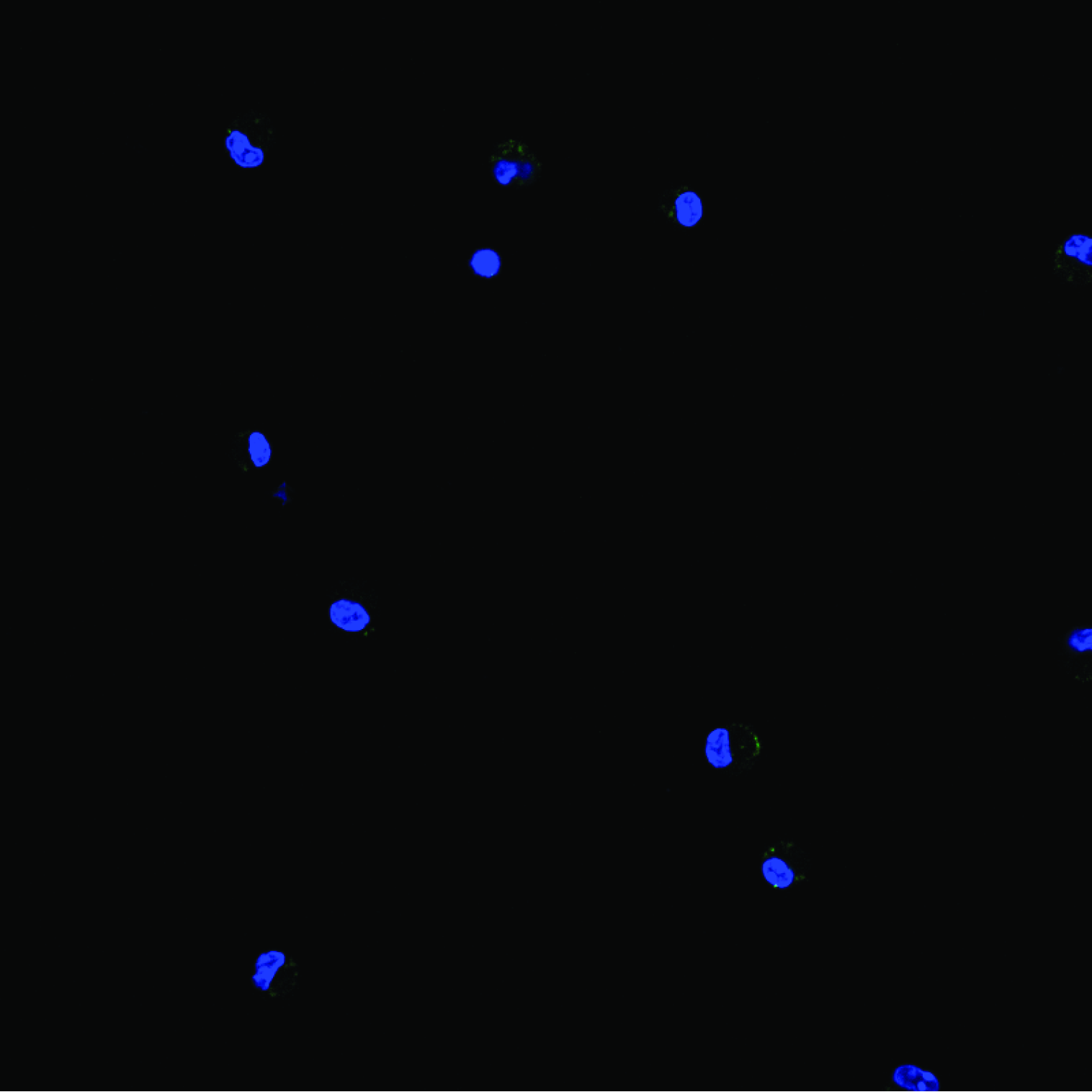

Supplement: Supplementary file 7 — Source data Fig. 6 [file 44318_2026_712_MOESM7_ESM.zip › Figure 6/6D/WTDMSO+MLSI3.tif]

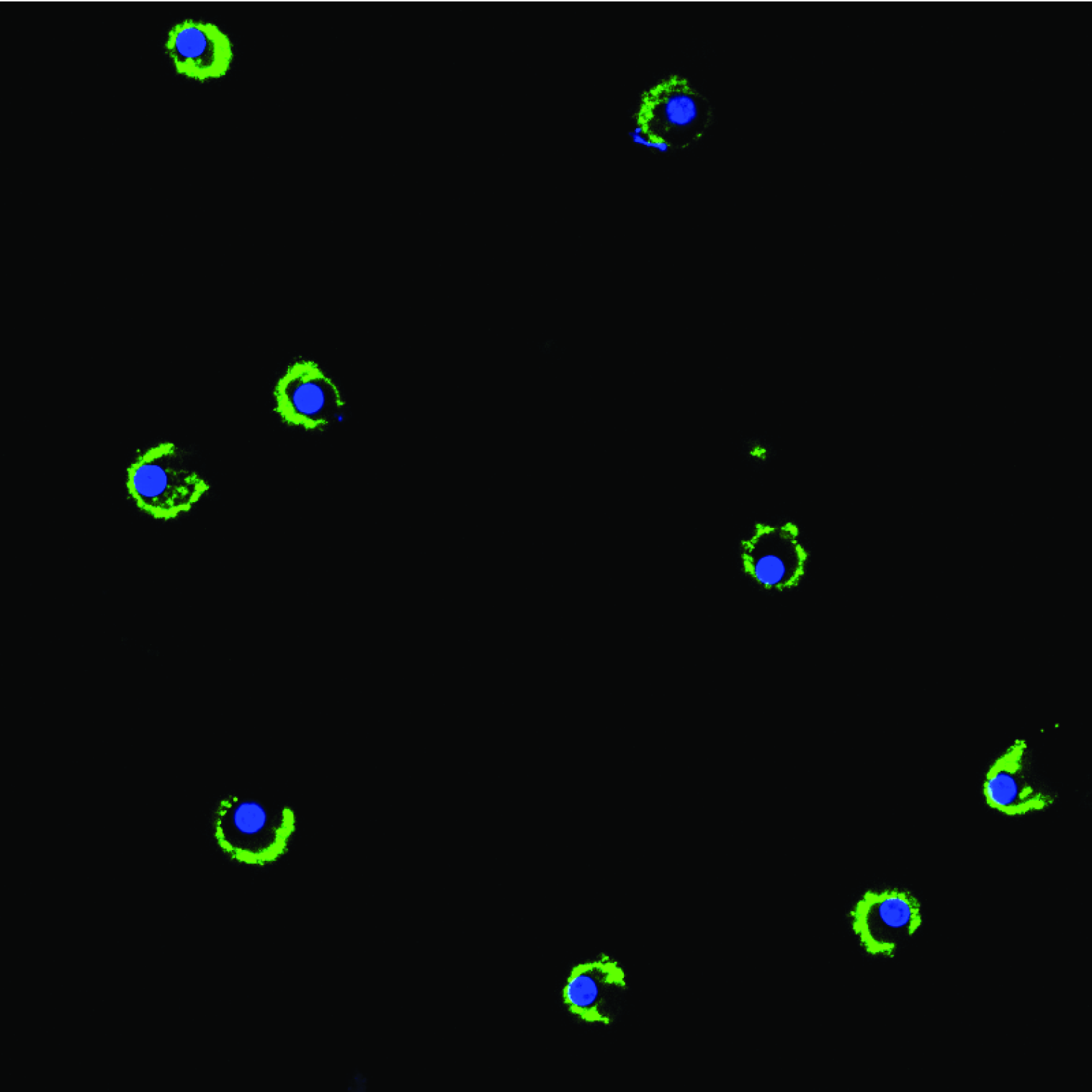

Supplement: Supplementary file 7 — Source data Fig. 6 [file 44318_2026_712_MOESM7_ESM.zip › Figure 6/6D/WTIonomycin.tif]

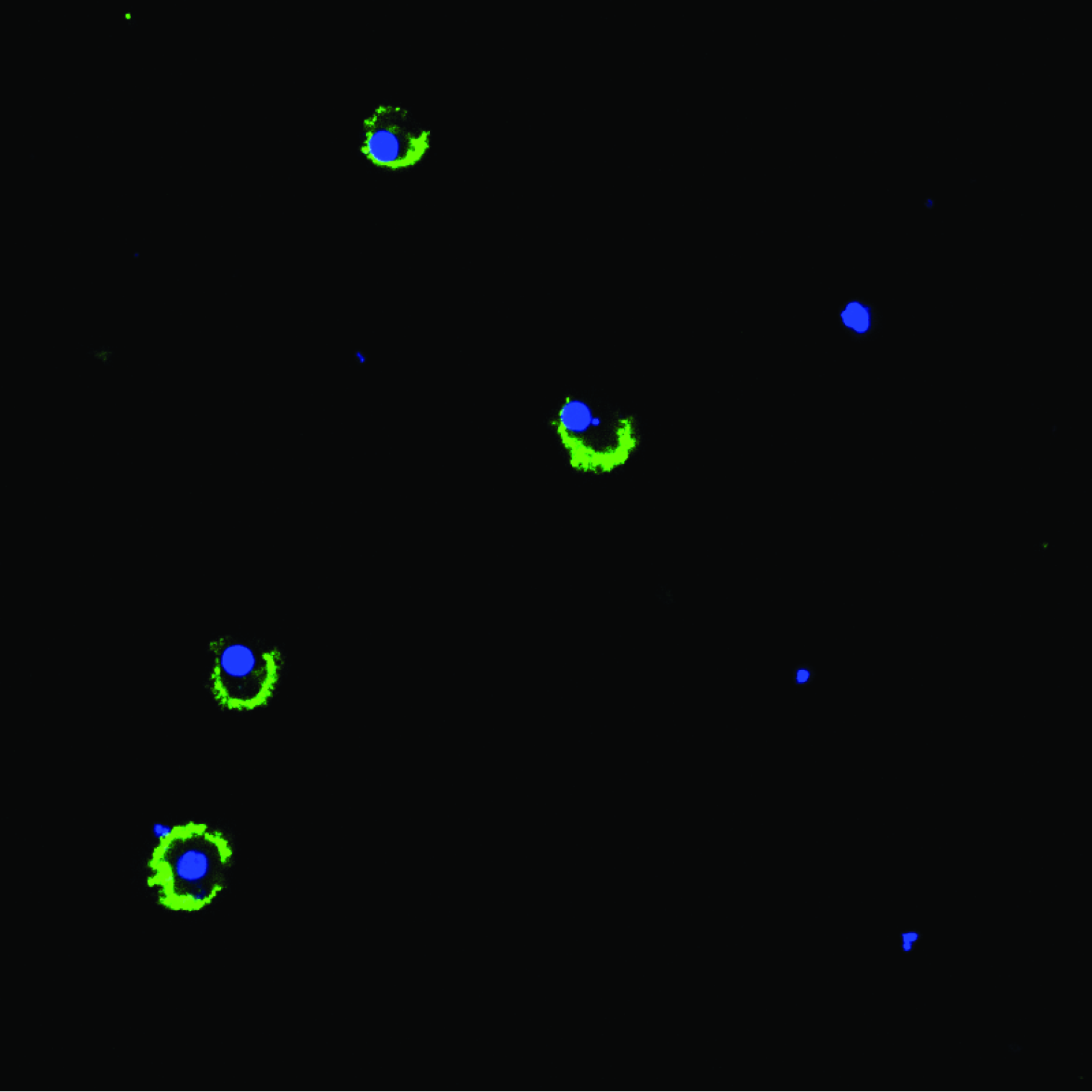

Supplement: Supplementary file 7 — Source data Fig. 6 [file 44318_2026_712_MOESM7_ESM.zip › Figure 6/6D/WTIonomycin+EDME.tif]

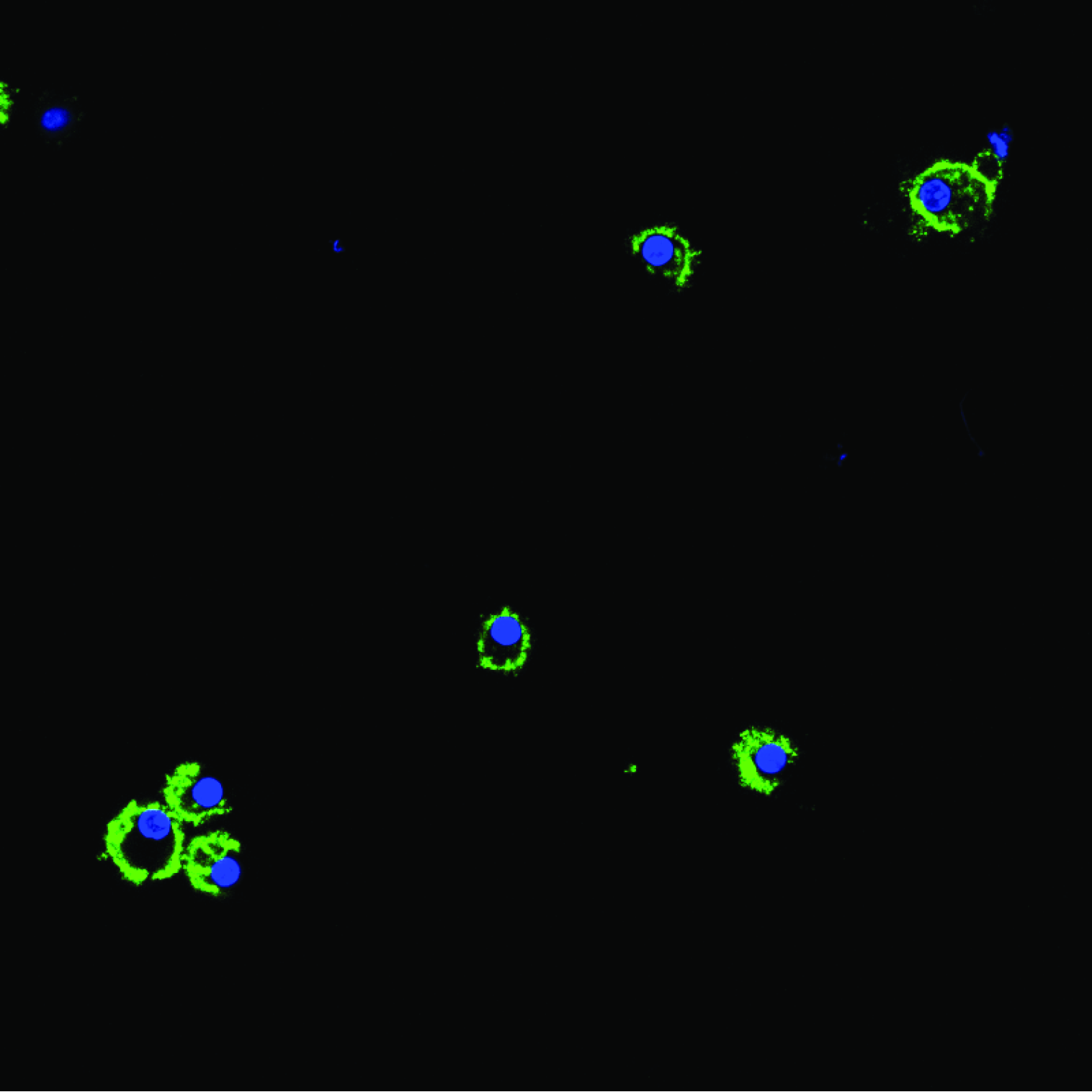

Supplement: Supplementary file 7 — Source data Fig. 6 [file 44318_2026_712_MOESM7_ESM.zip › Figure 6/6D/WTIonomycin+MLSI3.tif]

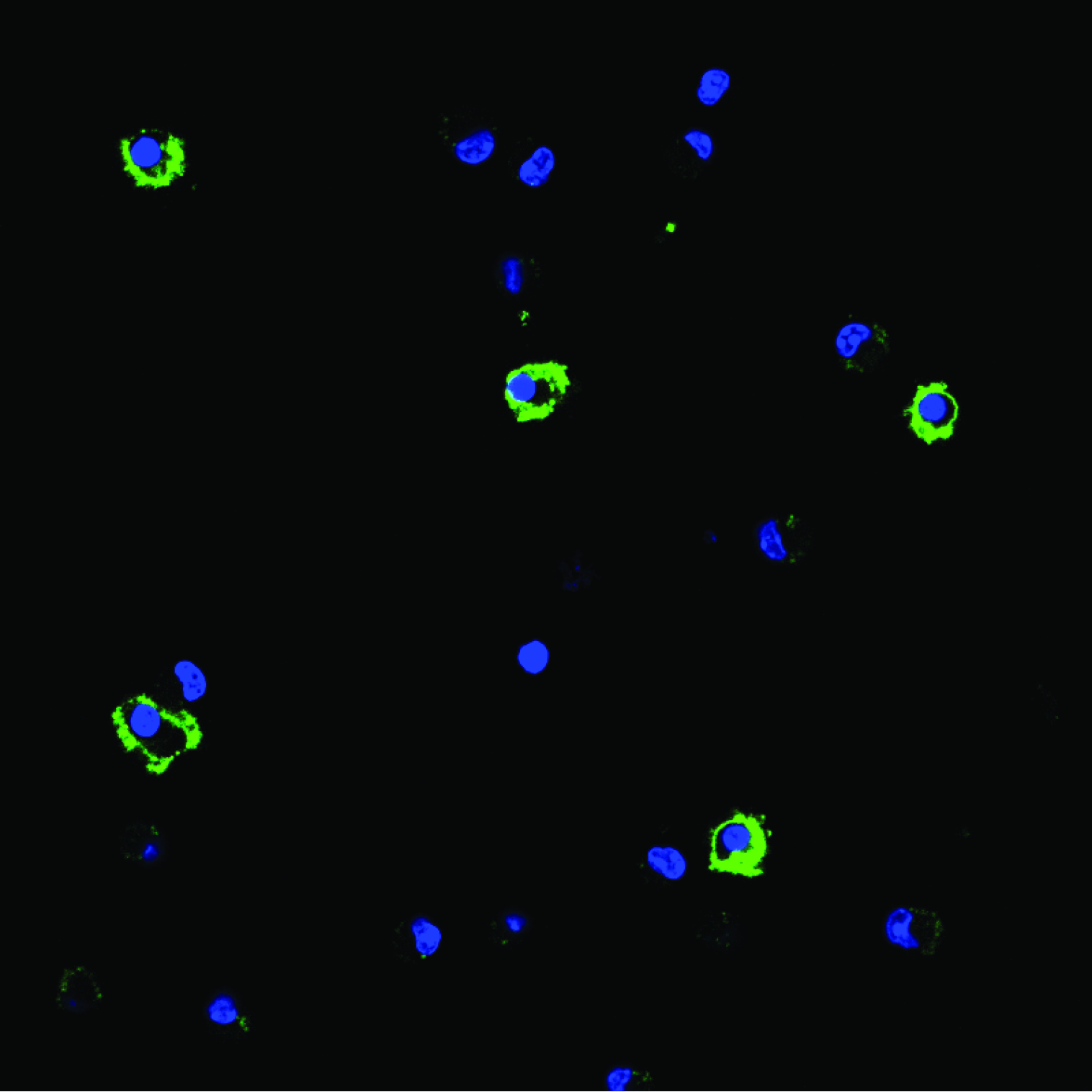

Supplement: Supplementary file 7 — Source data Fig. 6 [file 44318_2026_712_MOESM7_ESM.zip › Figure 6/6D/WTMLSA1.tif]

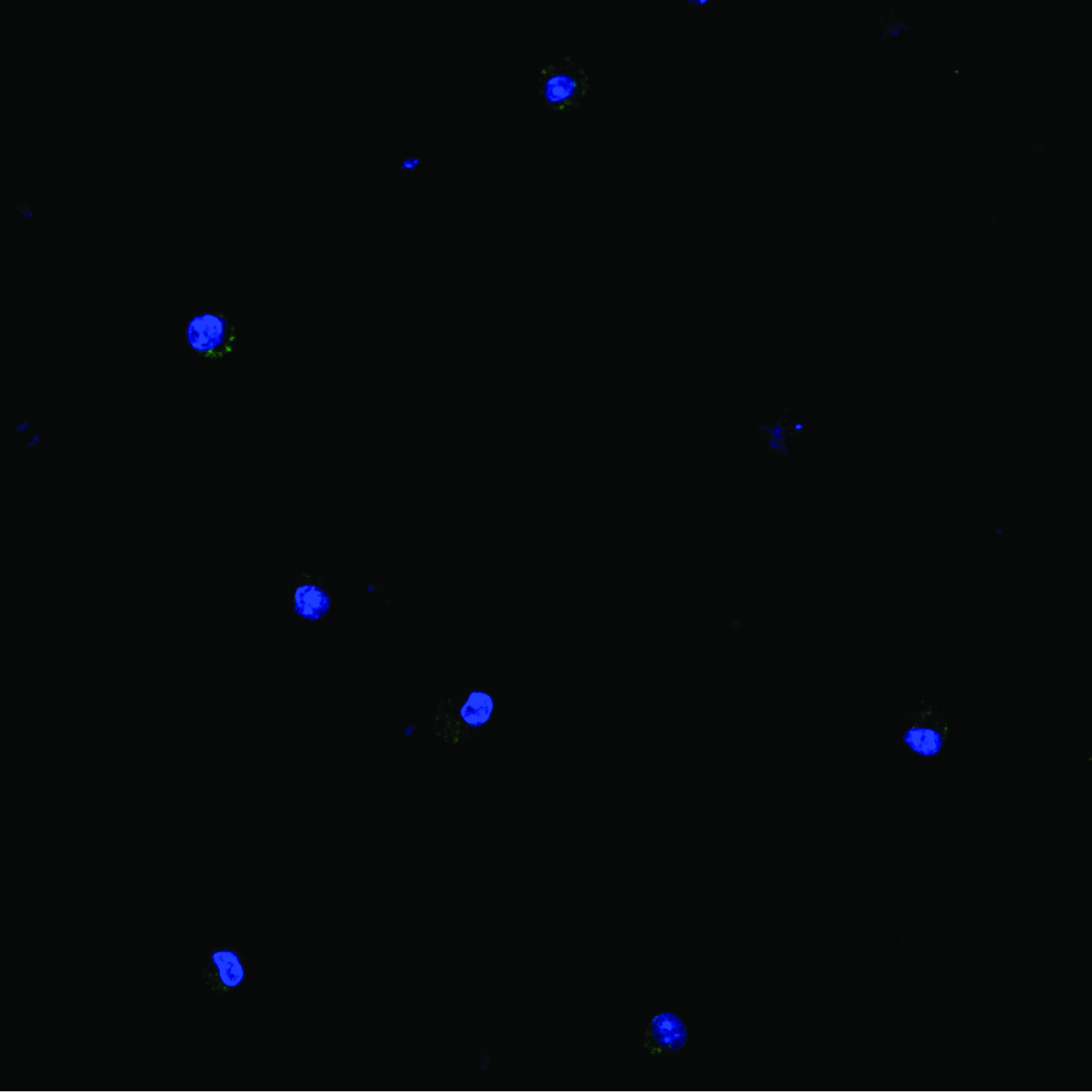

Supplement: Supplementary file 7 — Source data Fig. 6 [file 44318_2026_712_MOESM7_ESM.zip › Figure 6/6D/WTMLSA1+EDME.tif]

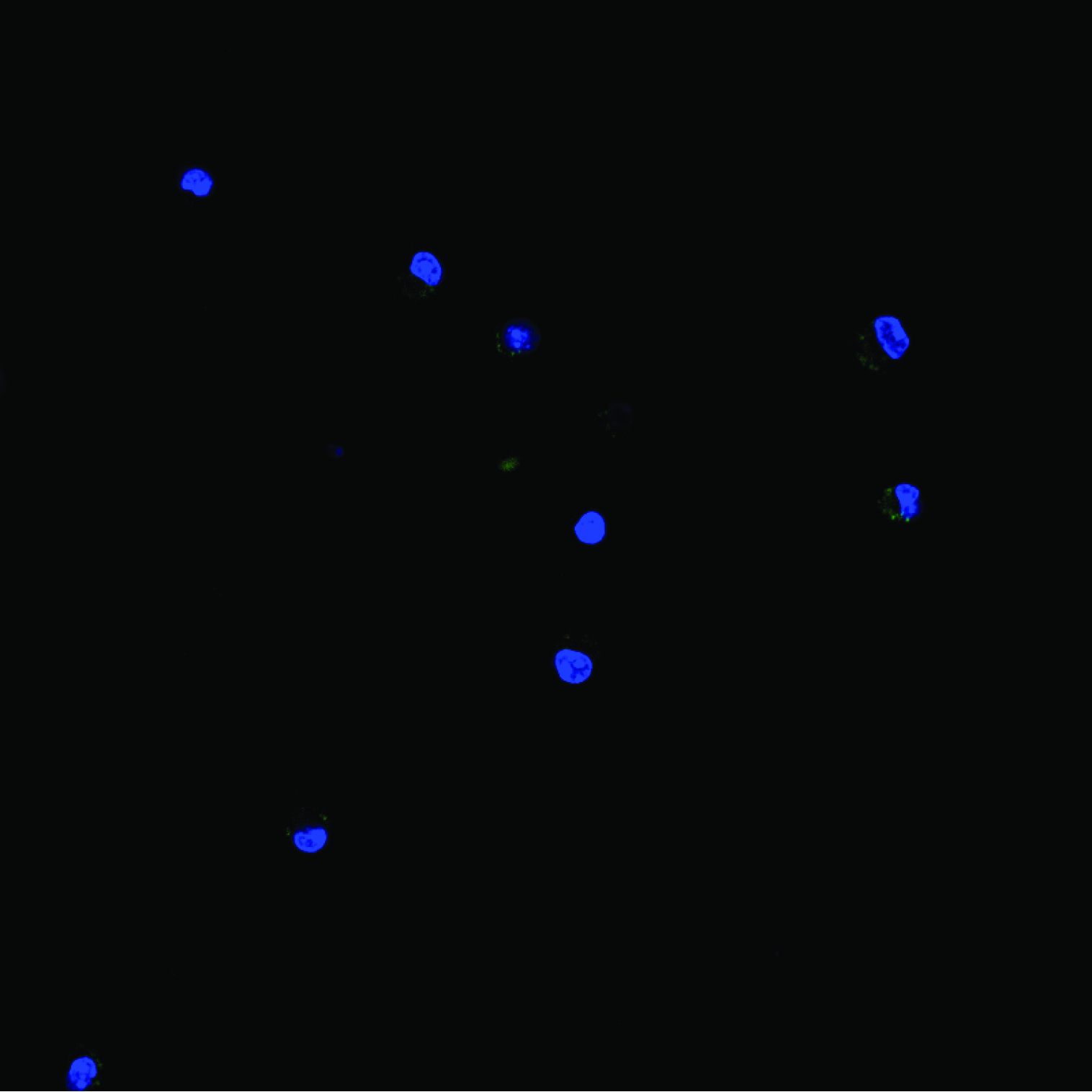

Supplement: Supplementary file 7 — Source data Fig. 6 [file 44318_2026_712_MOESM7_ESM.zip › Figure 6/6D/WTMLSA1+MLSI3.tif]

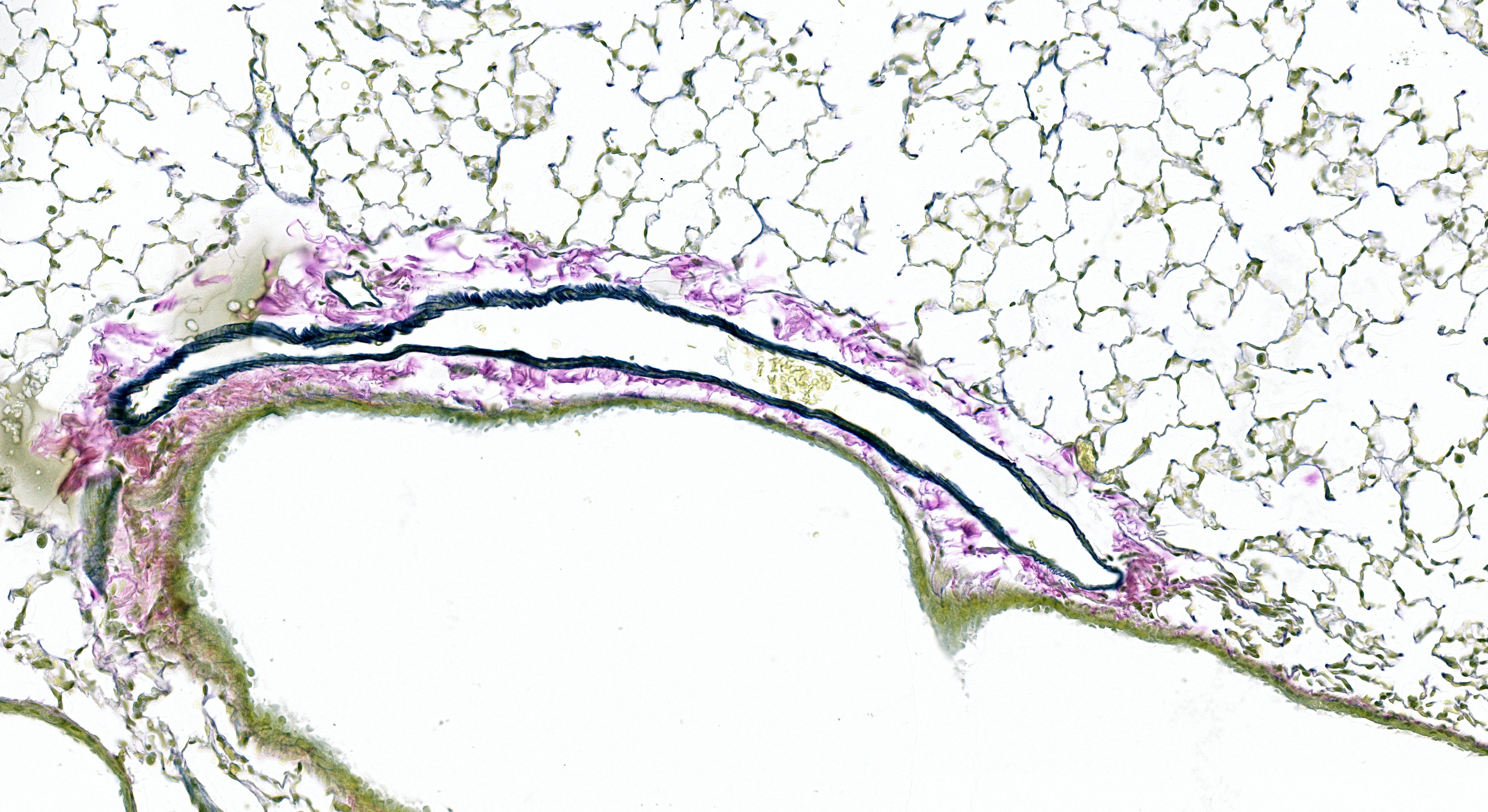

Supplement: Supplementary file 9 — Figure EV1 Source Data [file 44318_2026_712_MOESM9_ESM.zip › EV 1/EV1A/F32_WT_20x.tif]

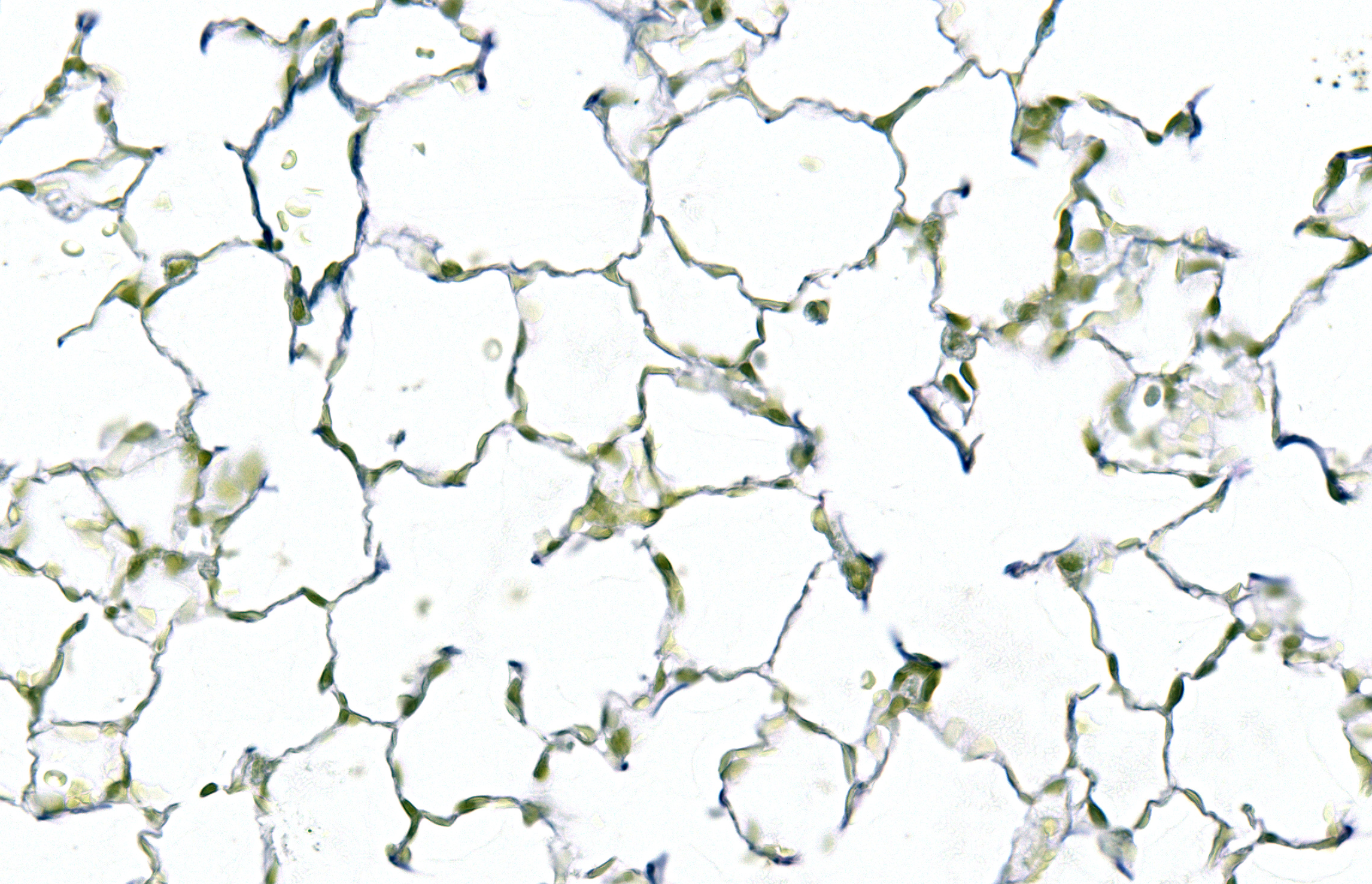

Supplement: Supplementary file 9 — Figure EV1 Source Data [file 44318_2026_712_MOESM9_ESM.zip › EV 1/EV1A/F32_WT_40x1.tif]

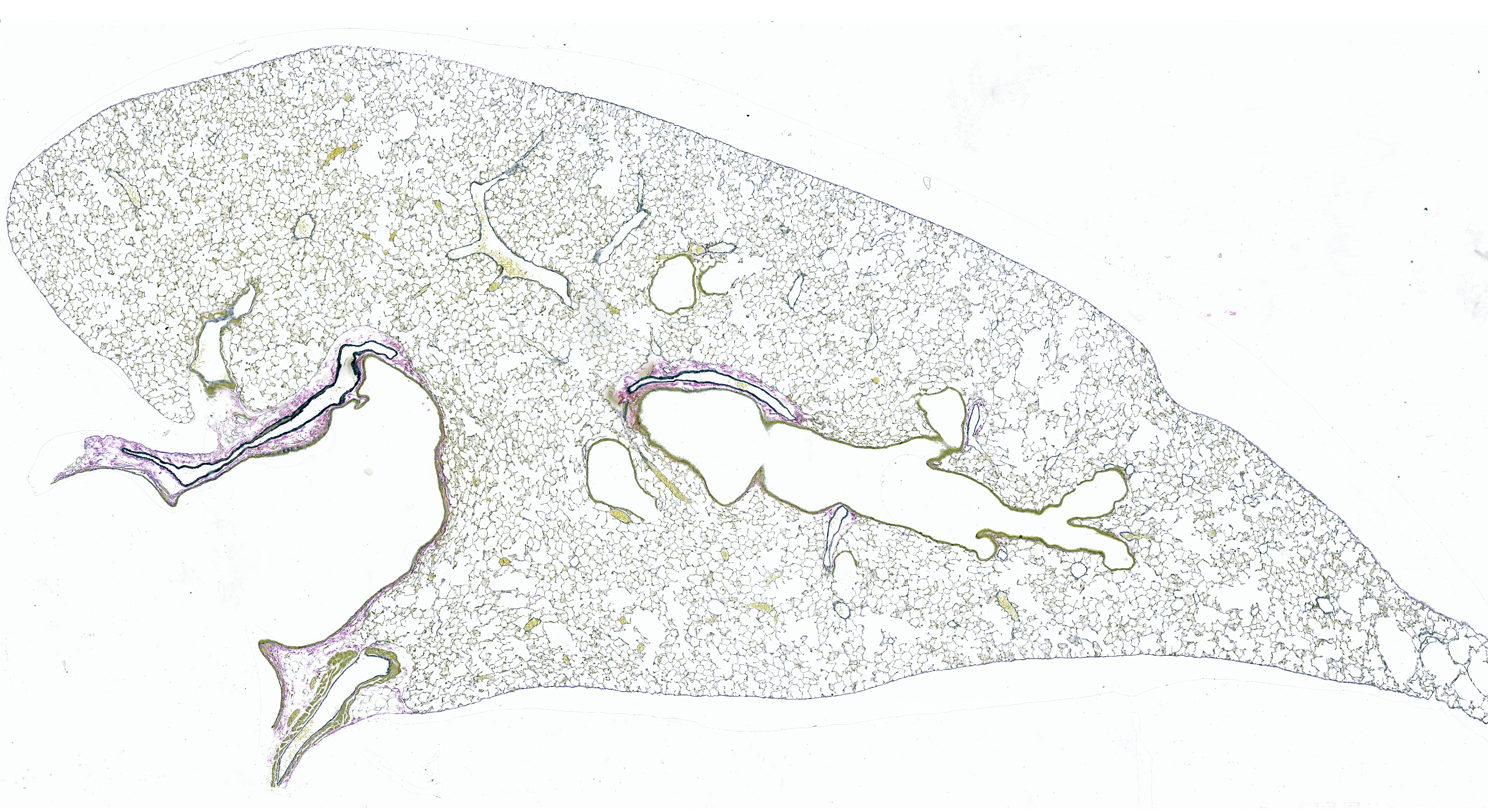

Supplement: Supplementary file 9 — Figure EV1 Source Data [file 44318_2026_712_MOESM9_ESM.zip › EV 1/EV1A/F32_WT_600um.tif]

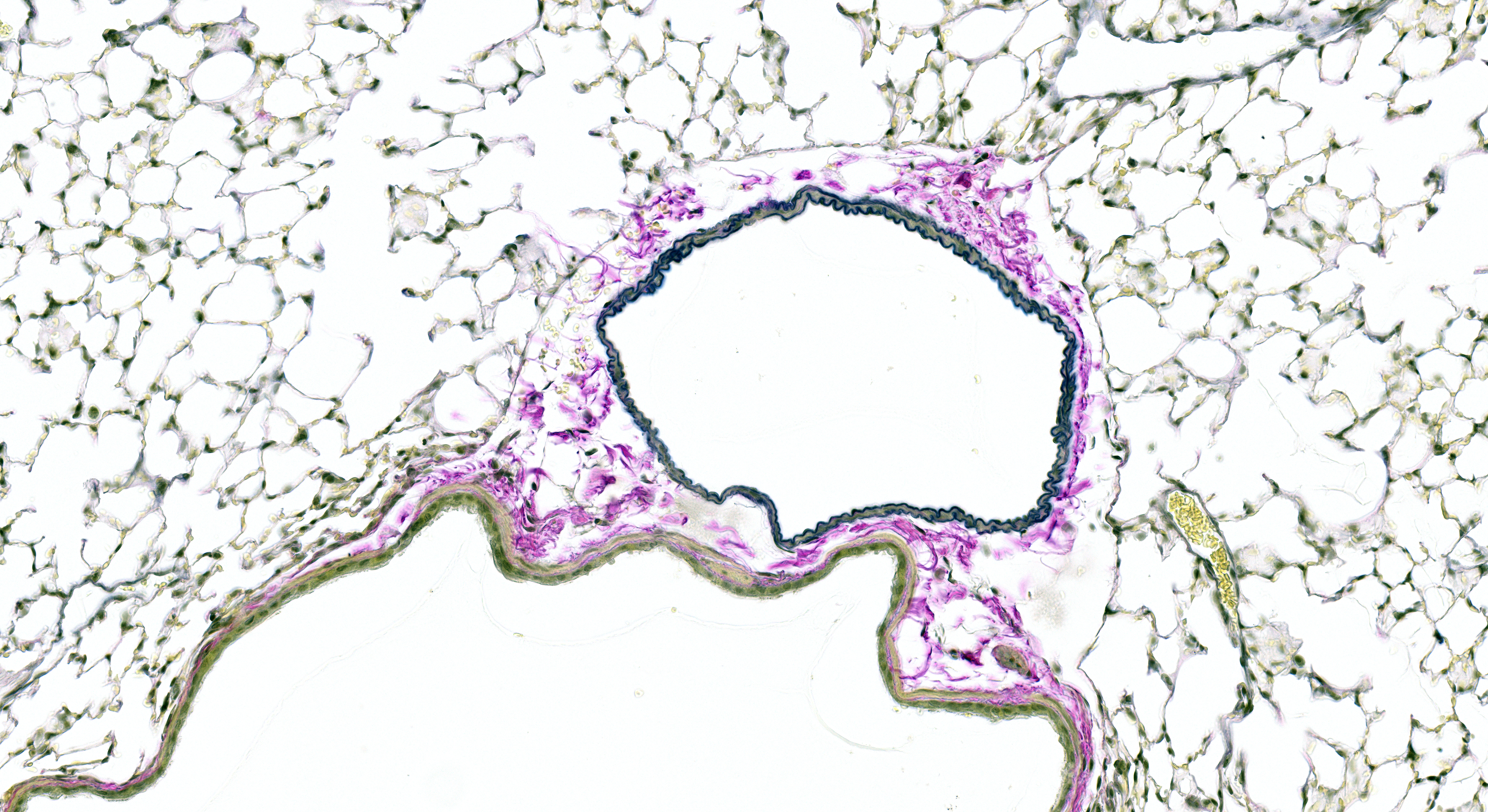

Supplement: Supplementary file 9 — Figure EV1 Source Data [file 44318_2026_712_MOESM9_ESM.zip › EV 1/EV1A/F33_Het_20x.tif]

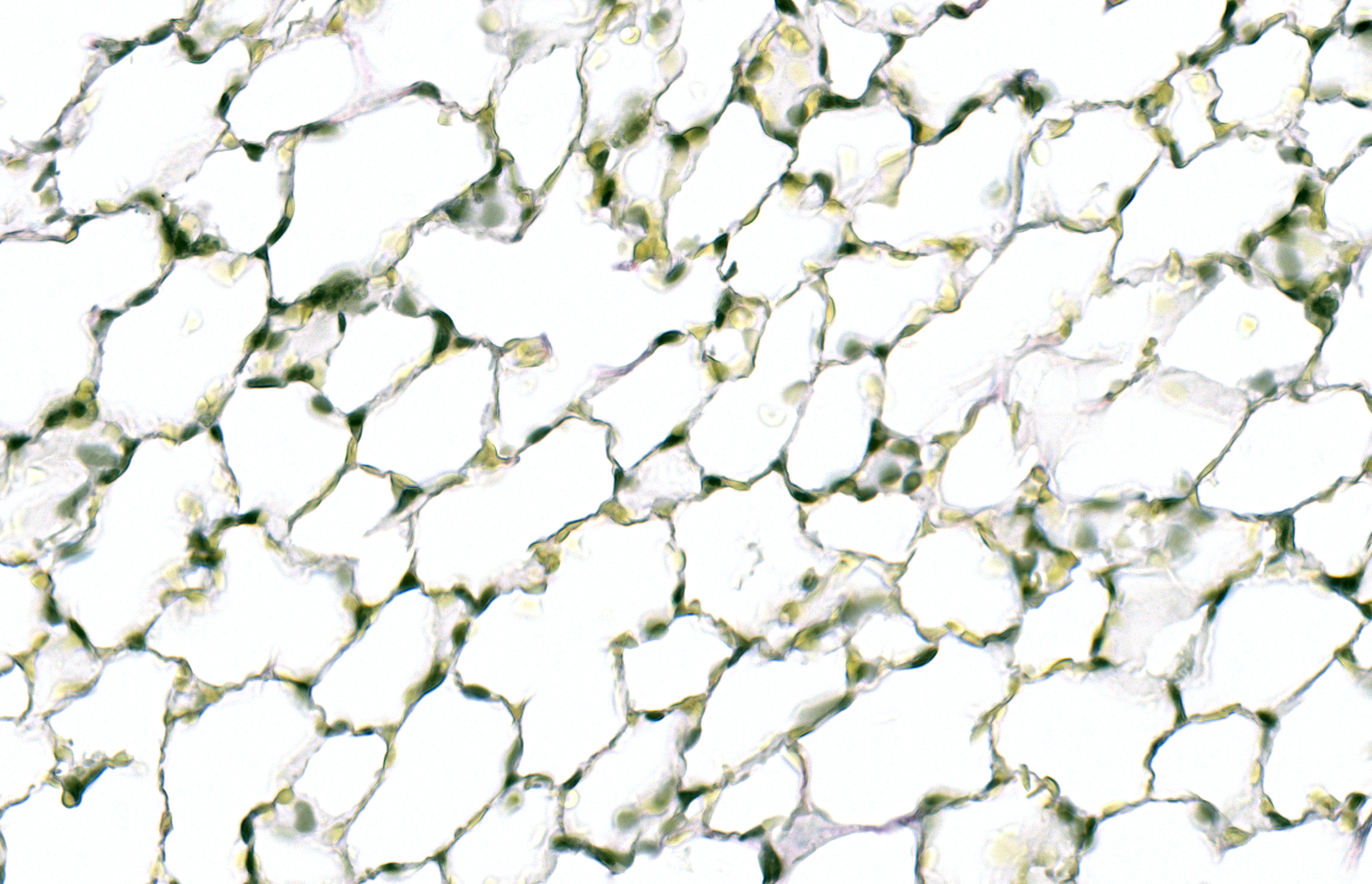

Supplement: Supplementary file 9 — Figure EV1 Source Data [file 44318_2026_712_MOESM9_ESM.zip › EV 1/EV1A/F33_Het_40x1.tif]

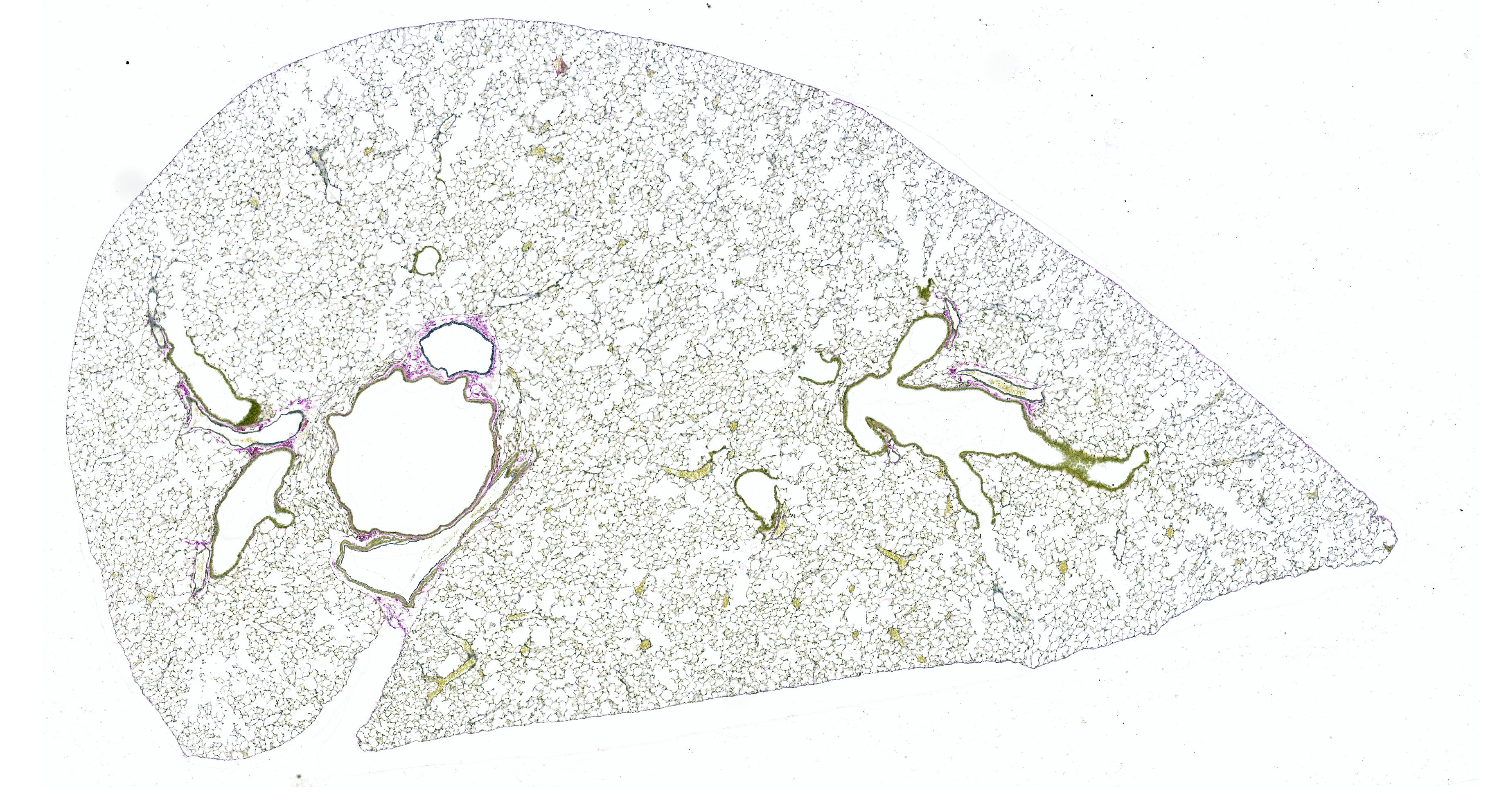

Supplement: Supplementary file 9 — Figure EV1 Source Data [file 44318_2026_712_MOESM9_ESM.zip › EV 1/EV1A/F33_Het_600um.tif]

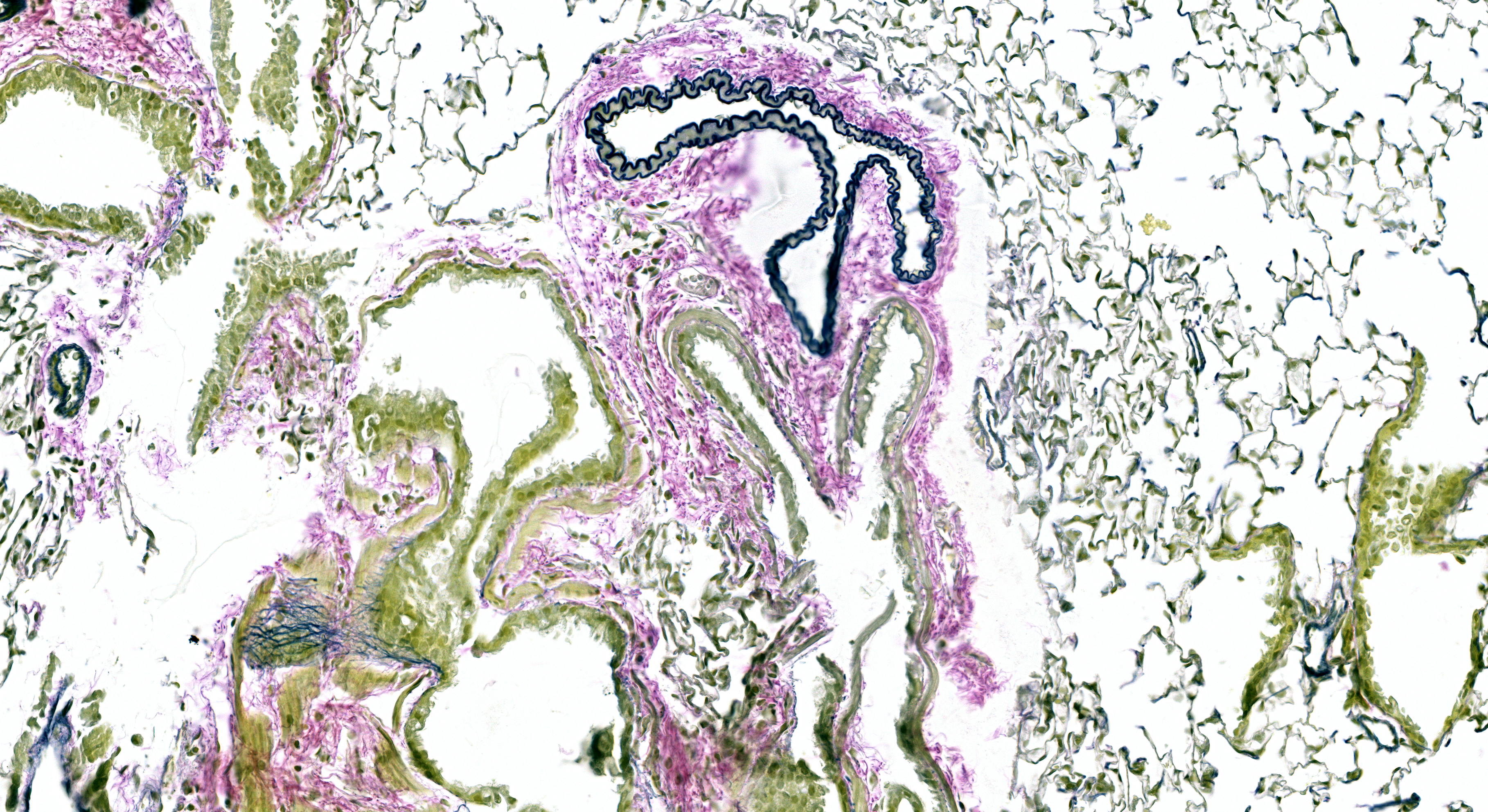

Supplement: Supplementary file 9 — Figure EV1 Source Data [file 44318_2026_712_MOESM9_ESM.zip › EV 1/EV1A/F78_KO_20x2.tif]

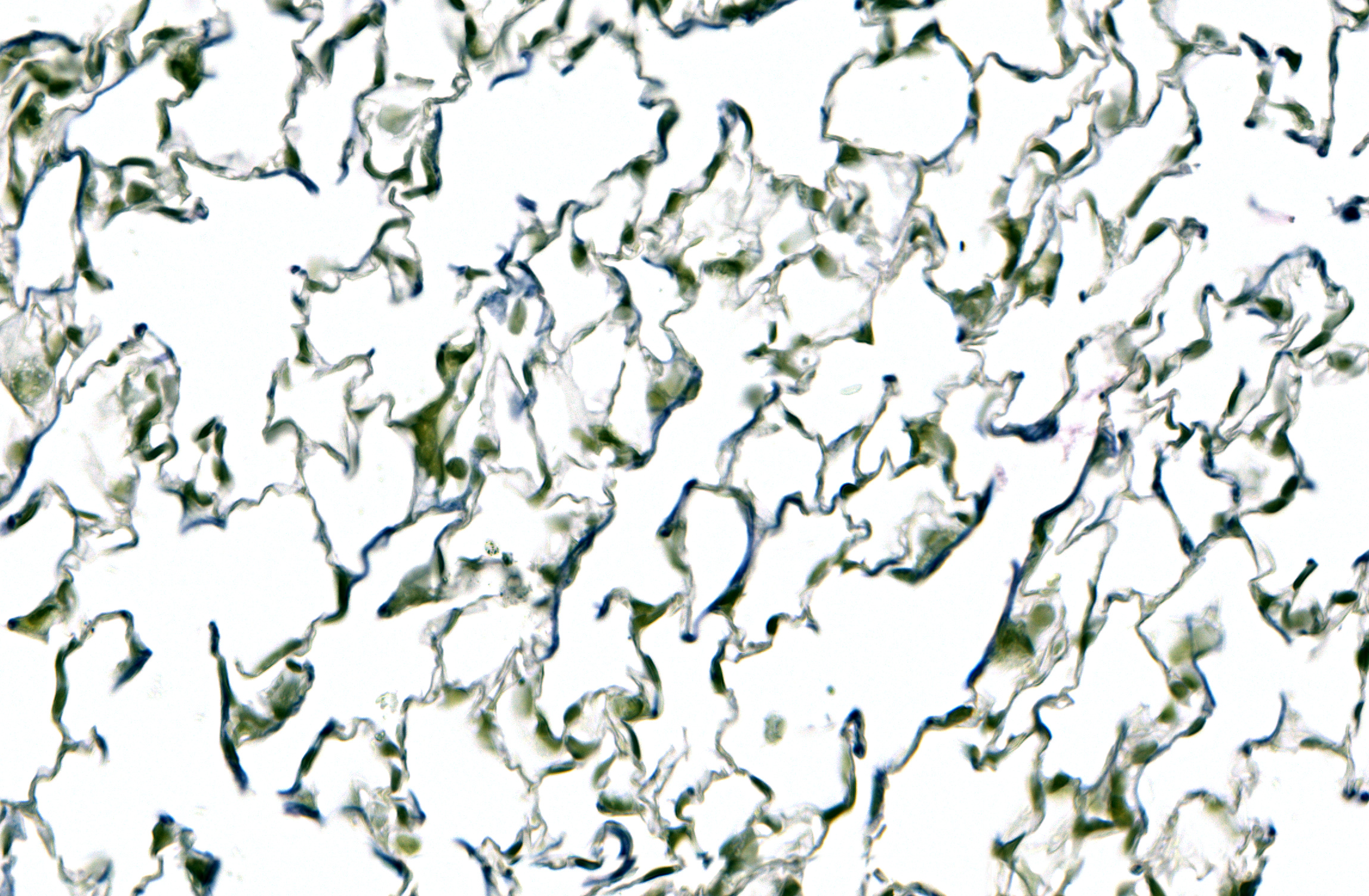

Supplement: Supplementary file 9 — Figure EV1 Source Data [file 44318_2026_712_MOESM9_ESM.zip › EV 1/EV1A/F78_KO_40x1.tif]

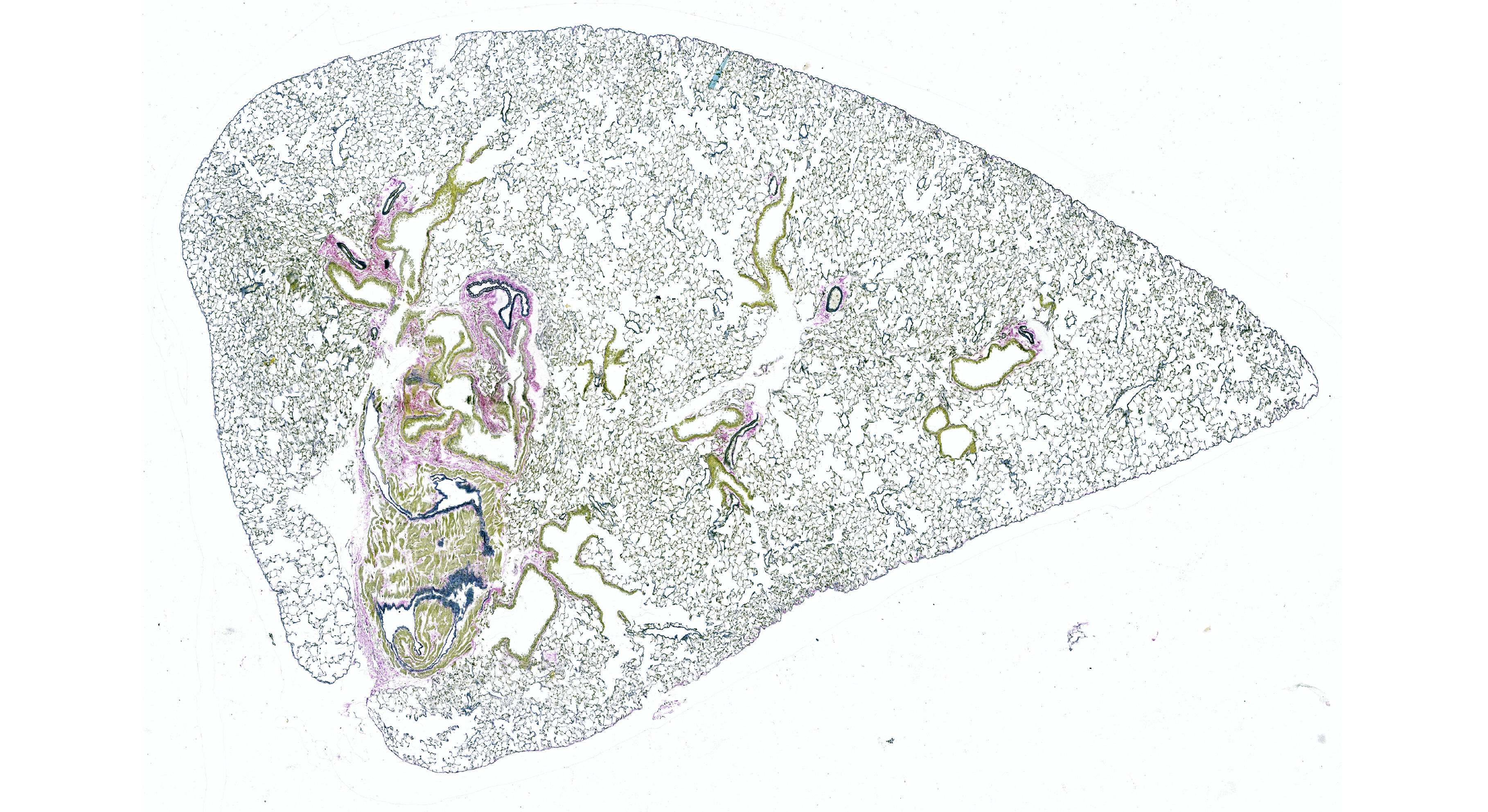

Supplement: Supplementary file 9 — Figure EV1 Source Data [file 44318_2026_712_MOESM9_ESM.zip › EV 1/EV1A/F78_KO_600um.tif]

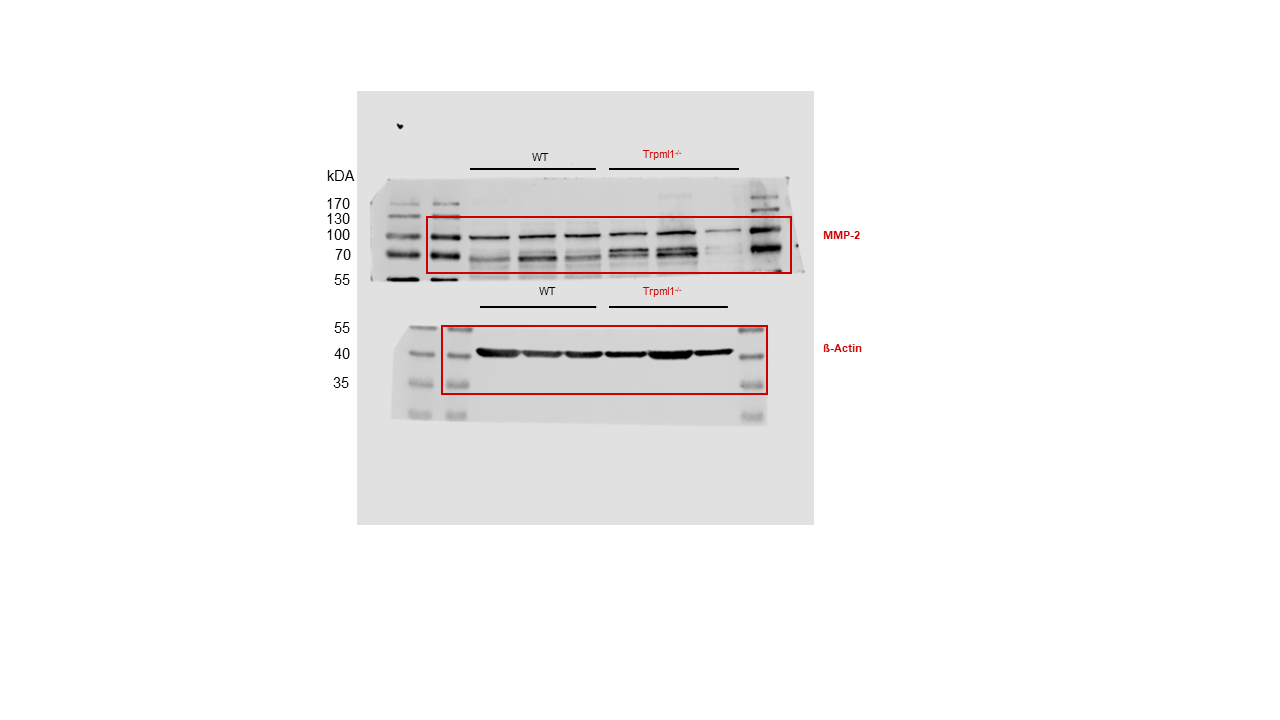

Supplement: Supplementary file 10 — Figure EV2 Source Data [file 44318_2026_712_MOESM10_ESM.zip › EV2/EV2 G-J/EV2J - WB-raw-mmp2-betaactin.tif]

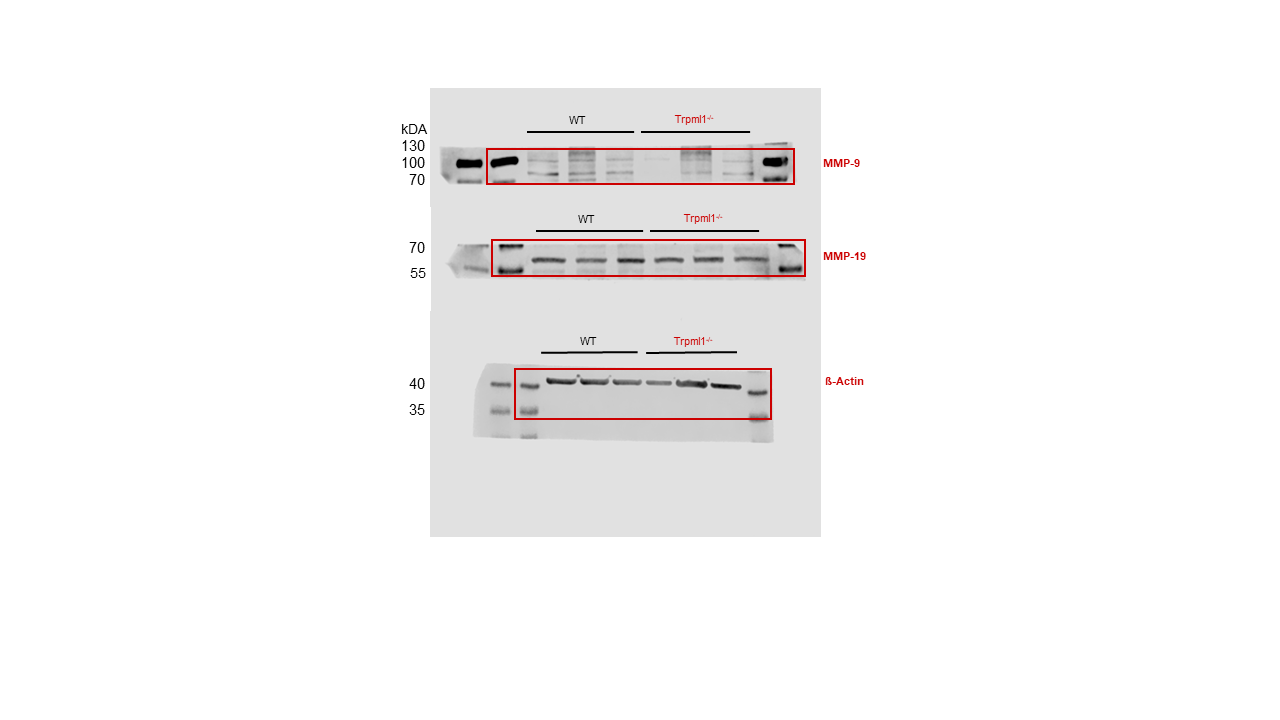

Supplement: Supplementary file 10 — Figure EV2 Source Data [file 44318_2026_712_MOESM10_ESM.zip › EV2/EV2 G-J/EV2J-WB-raw-mmp9-mmp19-betaactin.tif]

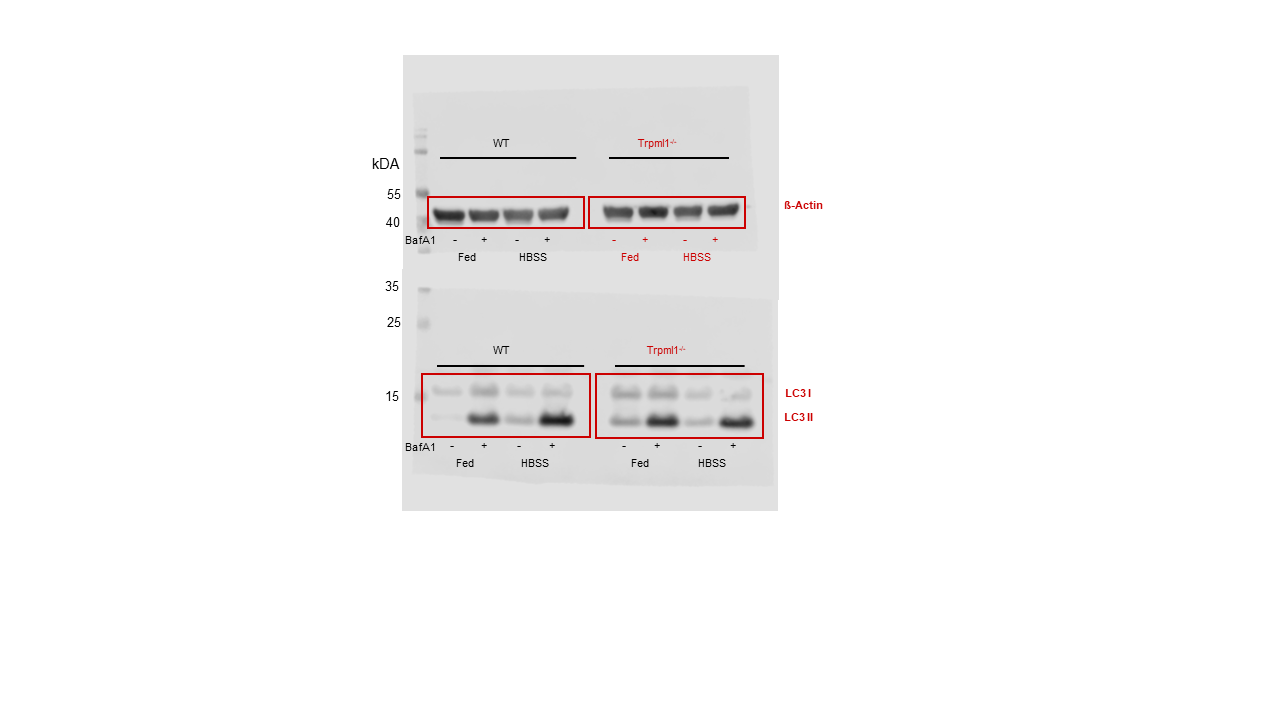

Supplement: Supplementary file 13 — Figure EV5 Source Data [file 44318_2026_712_MOESM13_ESM.zip › EV5/EV5A-B/EV5A_WB-raw-autophagy-LC3I-LC3II_betaactin.tif]

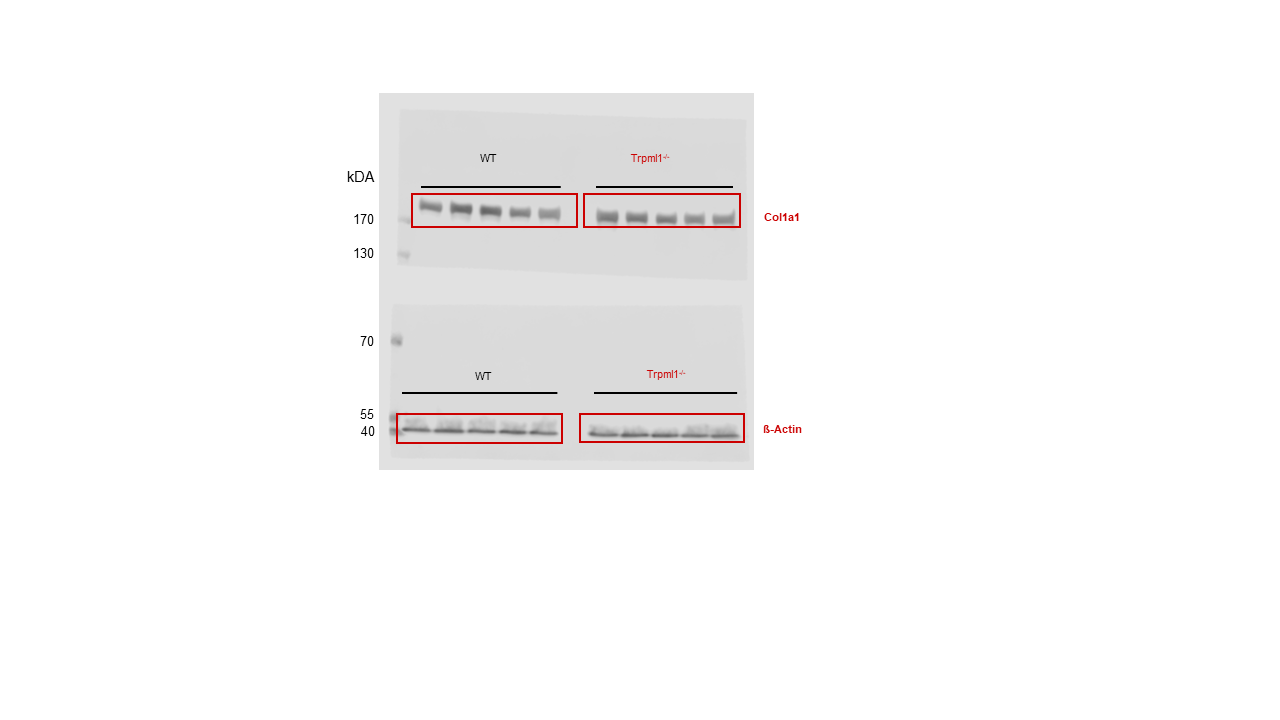

Supplement: Supplementary file 13 — Figure EV5 Source Data [file 44318_2026_712_MOESM13_ESM.zip › EV5/EV5C-D/EV5C_WB_raw-col1a1-betaactin.tif]

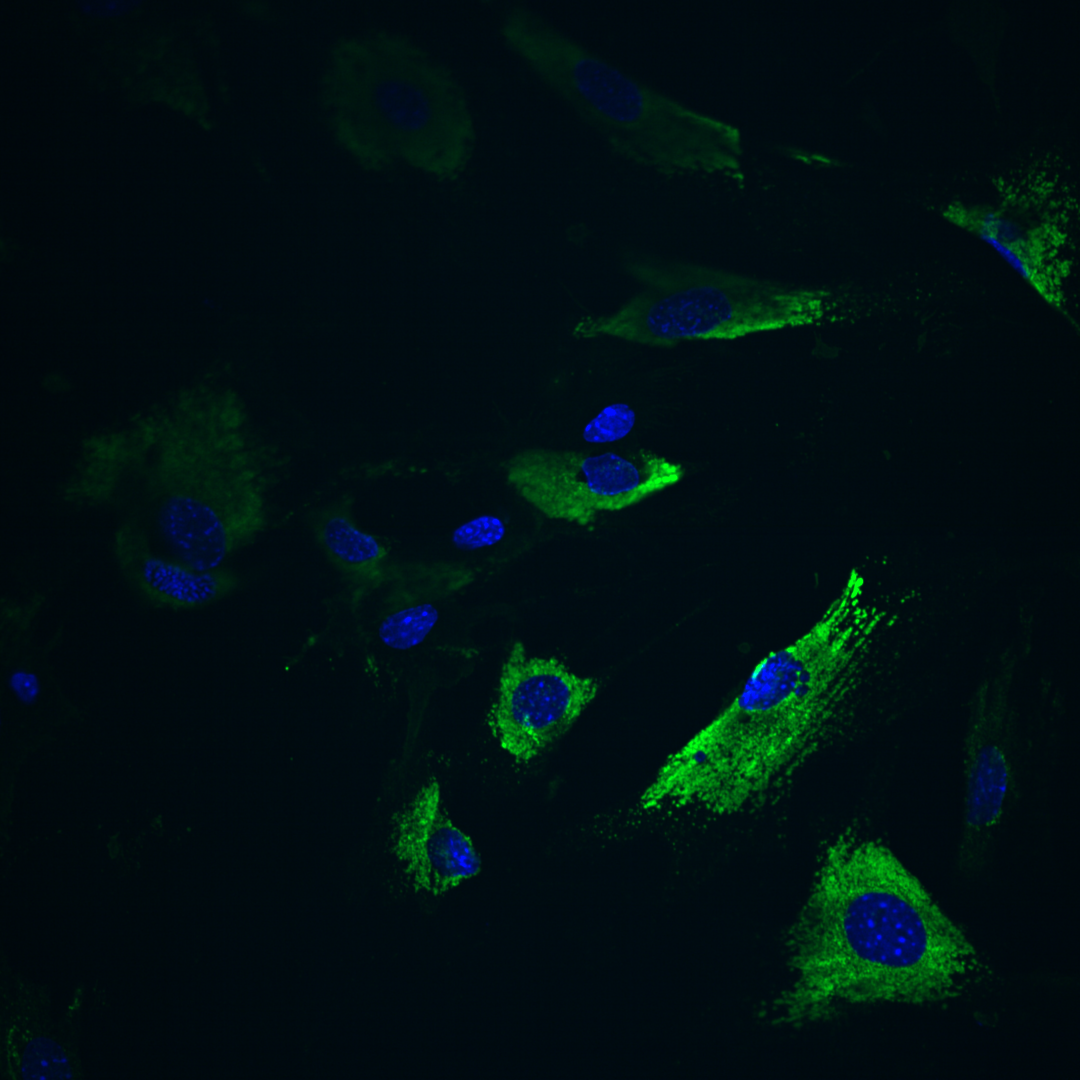

Supplement: Supplementary file 13 — Figure EV5 Source Data [file 44318_2026_712_MOESM13_ESM.zip › EV5/EV5F-G/EV5F opera KO.tif]

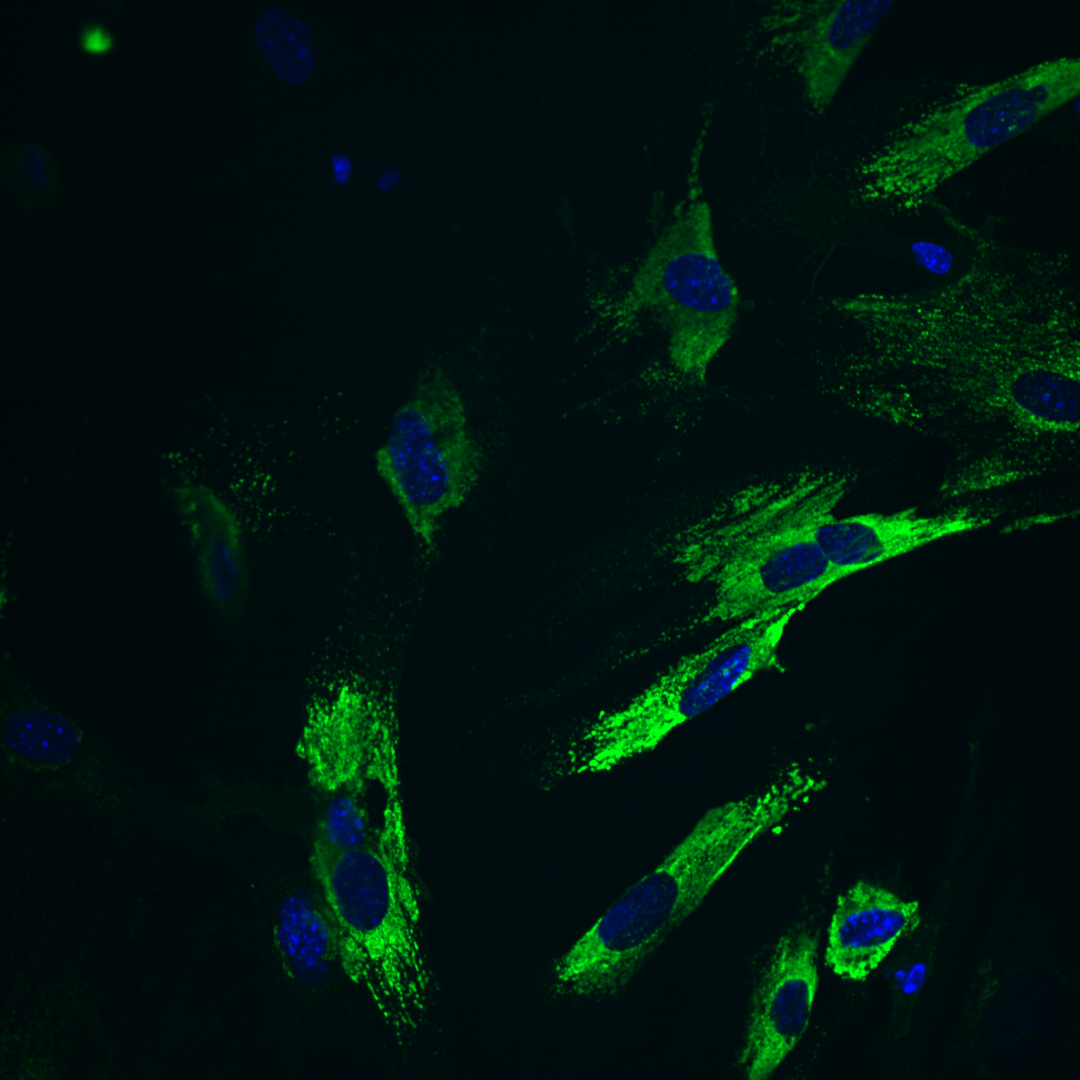

Supplement: Supplementary file 13 — Figure EV5 Source Data [file 44318_2026_712_MOESM13_ESM.zip › EV5/EV5F-G/EV5F Opera WT.tif]

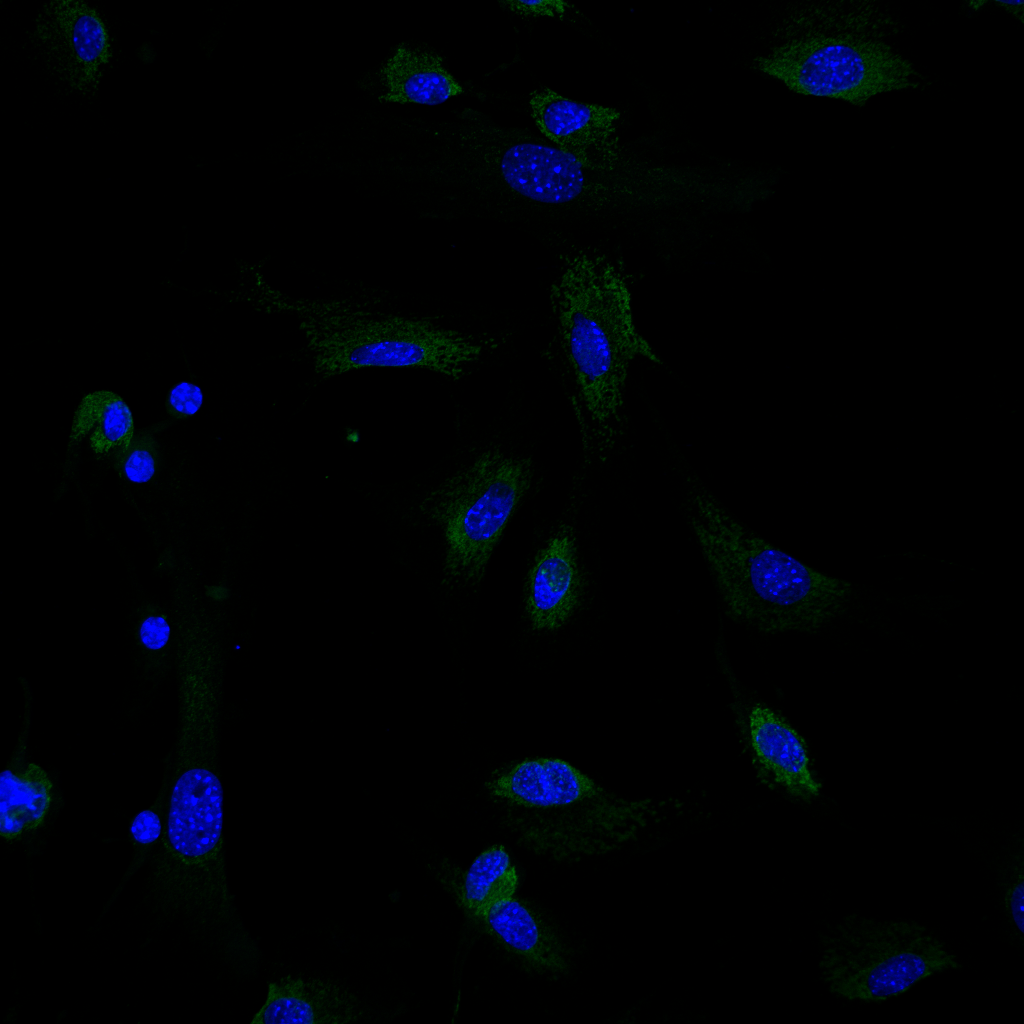

Supplement: Supplementary file 13 — Figure EV5 Source Data [file 44318_2026_712_MOESM13_ESM.zip › EV5/EV5F-G/EV5F- confocal_KO.tif]

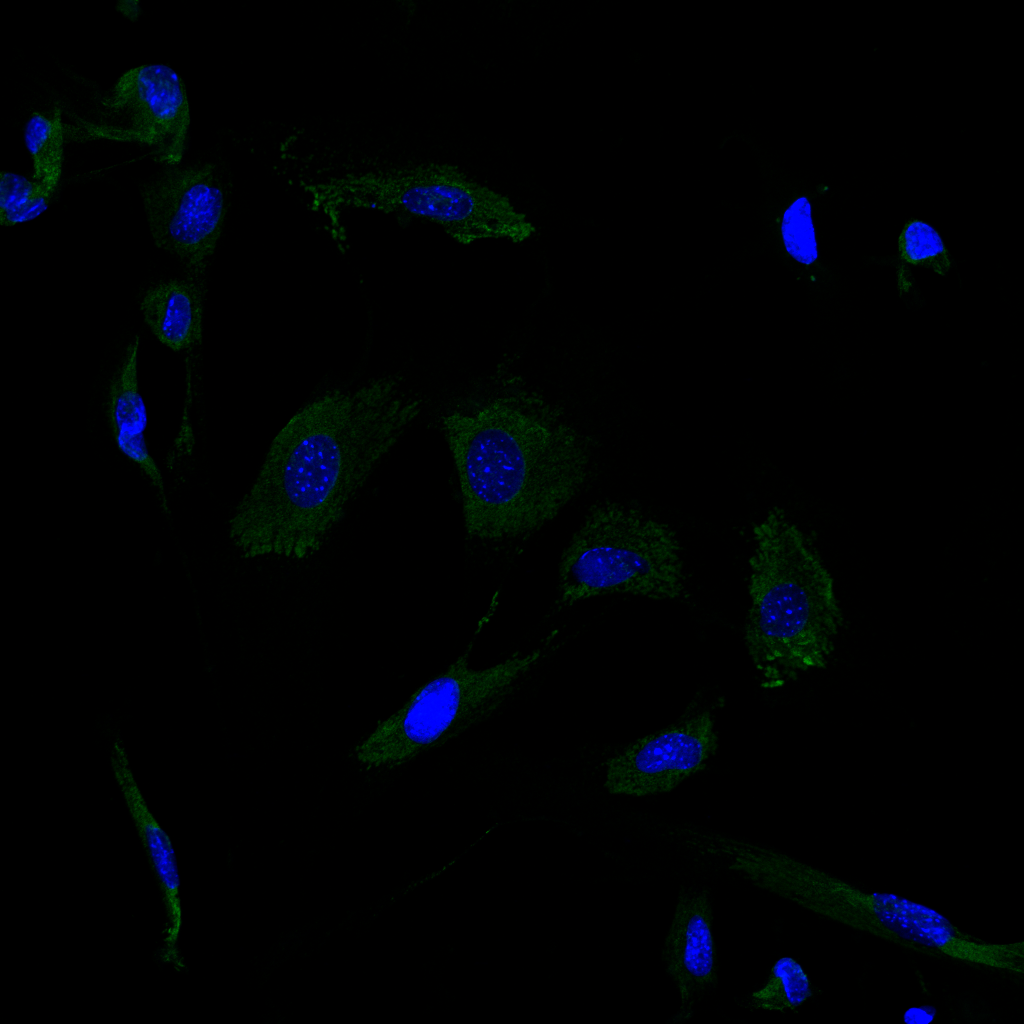

Supplement: Supplementary file 13 — Figure EV5 Source Data [file 44318_2026_712_MOESM13_ESM.zip › EV5/EV5F-G/EV5F- confocal_WT.tif]
