# Supplementary material for: TRPML1 suppresses pulmonary fibrosis by limiting collagen and elastin deposition
Source: EMBO J. 2026 Feb 19;45(7):2182–209. doi: 10.1038/s44318-026-00712-4 (PMC13043727; doi:10.1038/s44318-026-00712-4)

In [2]:
```python
import numpy as np
import matplotlib.pyplot as plt
import scanpy as sc
import pandas as pd
import seaborn as sb

# Some custom functions
import ma_codes as codes
from ma_codes import mysize, mymap
sc.set_figure_params(vector_friendly=True, dpi_save=300)


sc.settings.verbosity = 3             # verbosity: errors (0), warnings (1), info (2), hints (3)
sc.logging.print_version_and_date()
```

```
Running Scanpy 1.8.1, on 2025-06-30 18:56.
```

In [5]:
```python
adata
```

Out[5]:
```
AnnData object with n_obs × n_vars = 62635 × 27575
    obs: 'n_counts', 'condition', 'data_set', 'name', 'time_point', 'treatment', 'identifier', 'n_genes', 'percent_mito', 'QC_group', 'doublet_scores', 'size_factors', 'S_score', 'G2M_score', 'phase', 'time_point_all', 'louvain_1', 'louvain_2', 'low_qual', 'domain', 'cell_type', 'celltype_time', 'meta_celltype'
    var: 'n_cells', 'highly_variable', 'means', 'dispersions', 'dispersions_norm', 'highly_variable_nbatches', 'highly_variable_intersection'
    uns: 'cell_type_colors', 'condition_colors', 'domain_colors', 'hvg', 'identifier_colors', 'louvain', 'louvain_1_colors', 'louvain_2_colors', 'low_qual_colors', 'meta_celltype_colors', 'name_colors', 'neighbors', 'pca', 'time_point_colors', 'treatment_colors', 'umap'
    obsm: 'X_pca', 'X_umap'
    varm: 'PCs'
    layers: 'counts', 'unsouped_counts'
    obsp: 'connectivities', 'distances'
```

In [5]:
```python
sc.pl.umap(adata, color = ["time_point"], wspace = 0.2, size = 20,cmap=mymap )
```

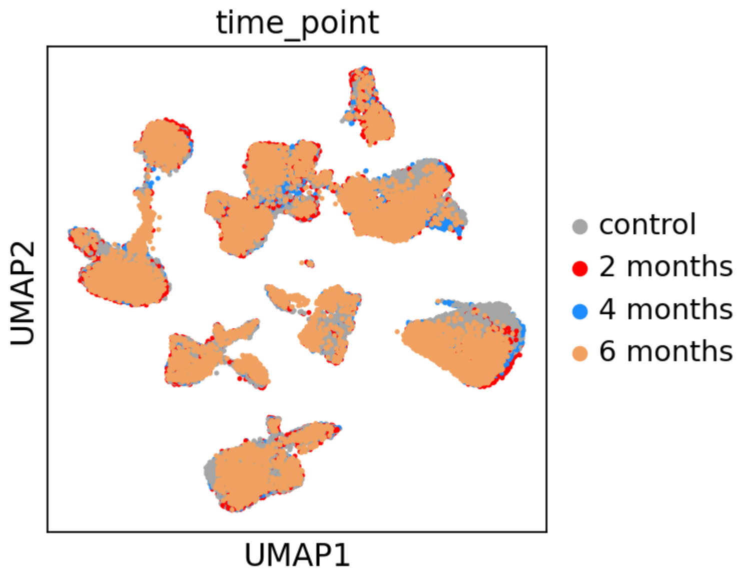

In [8]:
```
control = adata[adata.obs.time_point.isin(["control"])].copy()

sc.pl.umap(control, color = ["time_point"], wspace = 0.2, size = 20)
```

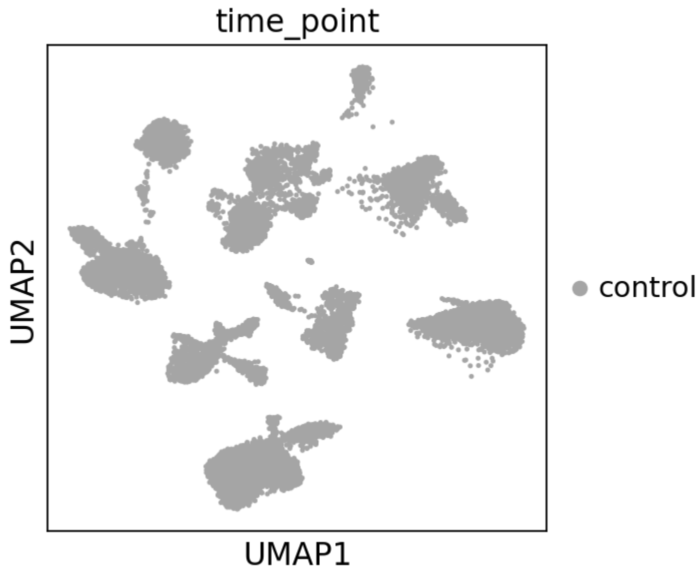

In [10]:
```
control
```

Out[10]:
```
AnnData object with n_obs × n_vars = 21439 × 27575
    obs: 'n_counts', 'condition', 'data_set', 'name', 'time_point', 'treatment', 'identifier', 'n_genes', 'percent_mito', 'QC_group', 'doublet_scores', 'size_factors', 'S_score', 'G2M_score', 'phase', 'time_point_all', 'louvain_1', 'louvain_2', 'low_qual', 'domain', 'cell_type', 'celltype_time', 'meta_celltype'
    var: 'n_cells', 'highly_variable', 'means', 'dispersions', 'dispersions_norm', 'highly_variable_nbatches', 'highly_variable_intersection'
    uns: 'cell_type_colors', 'condition_colors', 'domain_colors', 'hvg', 'identifier_colors', 'louvain', 'louvain_1_colors', 'louvain_2_colors', 'low_qual_colors', 'meta_celltype_colors', 'name_colors', 'neighbors', 'pca', 'time_point_colors', 'treatment_colors', 'umap'
    obsm: 'X_pca', 'X_umap'
    varm: 'PCs'
    layers: 'counts', 'unsouped_counts'
    obsp: 'connectivities', 'distances'
```

In [22]:
```
sc.pl.umap(control, color = ['Mcoln1',"cell_type"], wspace = 0.2, size = 20,cmap=mymap )
```

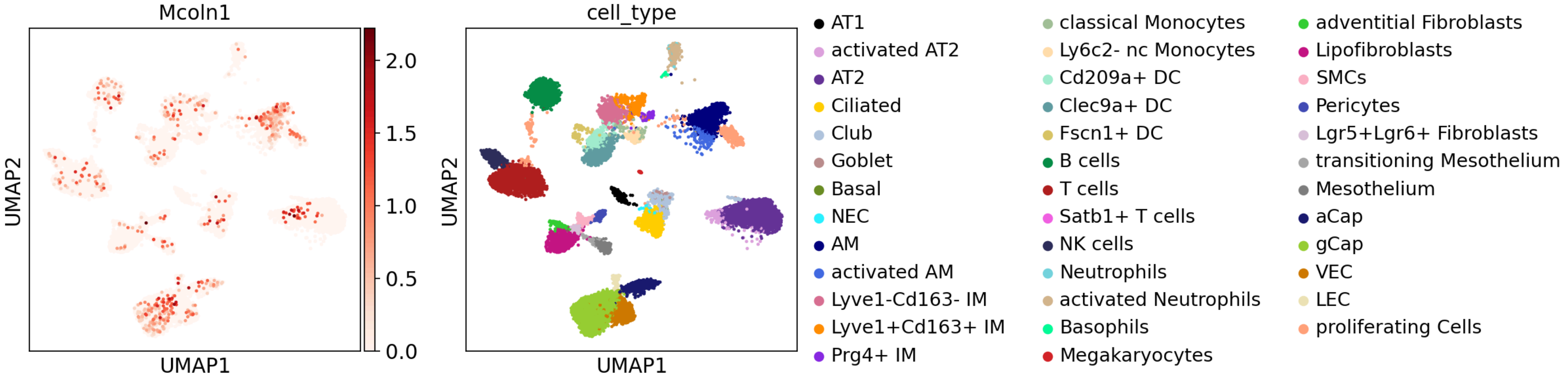

In [33]:
```
sc.pl.umap(control, color = ['Mmp10', 'Mmp11', 'Mmp15', 'Mmp16', 'Mmp17','Mmp19', 'Mmp20', 'Mmp21', 'Mmp23',
                             'Mmp24', 'Mmp25', 'Mmp27', 'Mmp28'], ncols=2, cmap =mymap, size = 80,vmin=0.1)
```

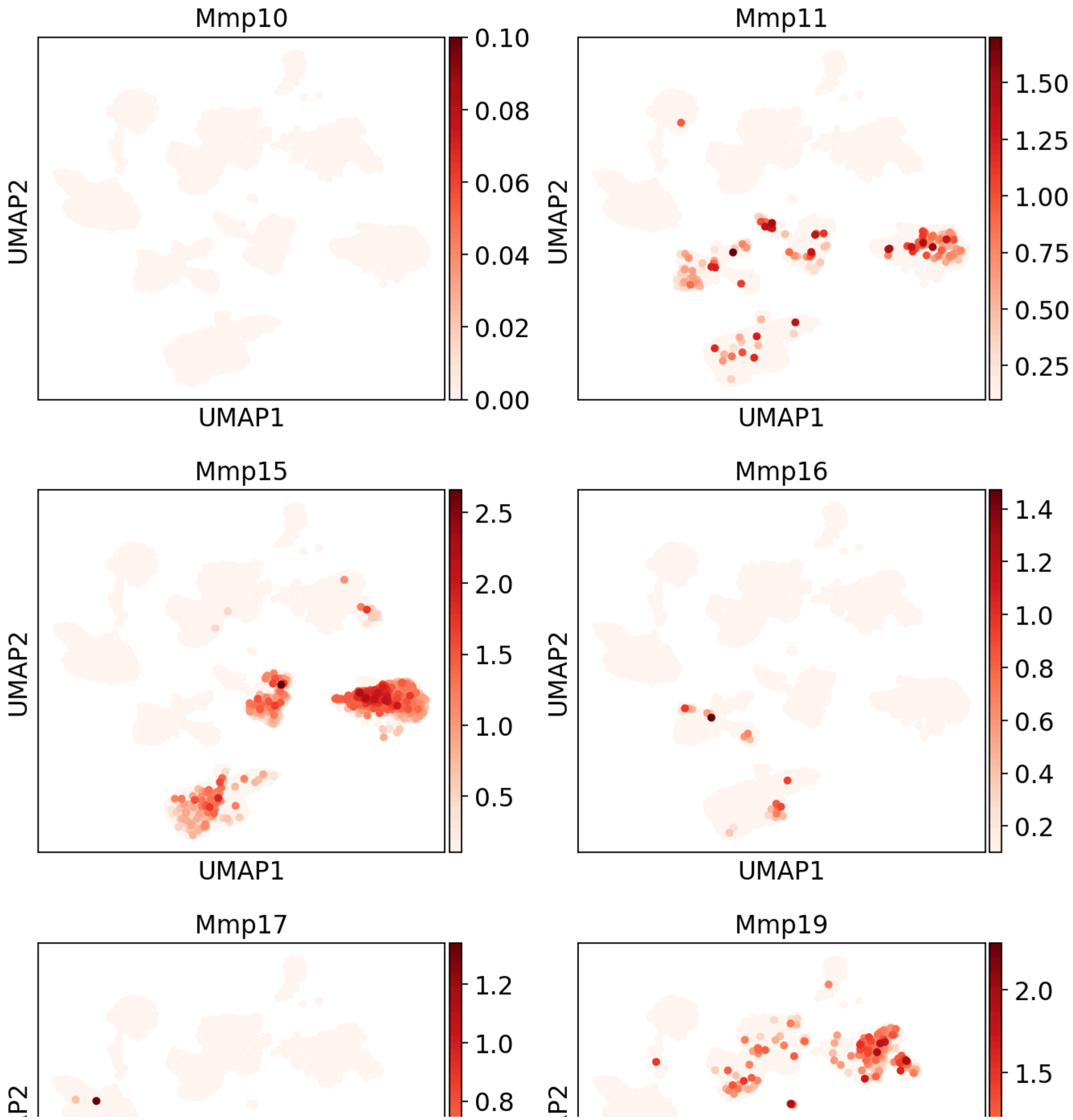

Mmp10
0.10
0.08
0.06
0.04
0.02
0.00
UMAP2
UMAP1
Mmp11
1.50
1.25
1.00
0.75
0.50
0.25
UMAP2
UMAP1
Mmp15
2.5
2.0
1.5
1.0
0.5
UMAP2
UMAP1
Mmp16
1.4
1.2
1.0
0.8
0.6
0.4
0.2
UMAP2
UMAP1
Mmp17
1.2
1.0
0.8
Mmp19
2.0
1.5

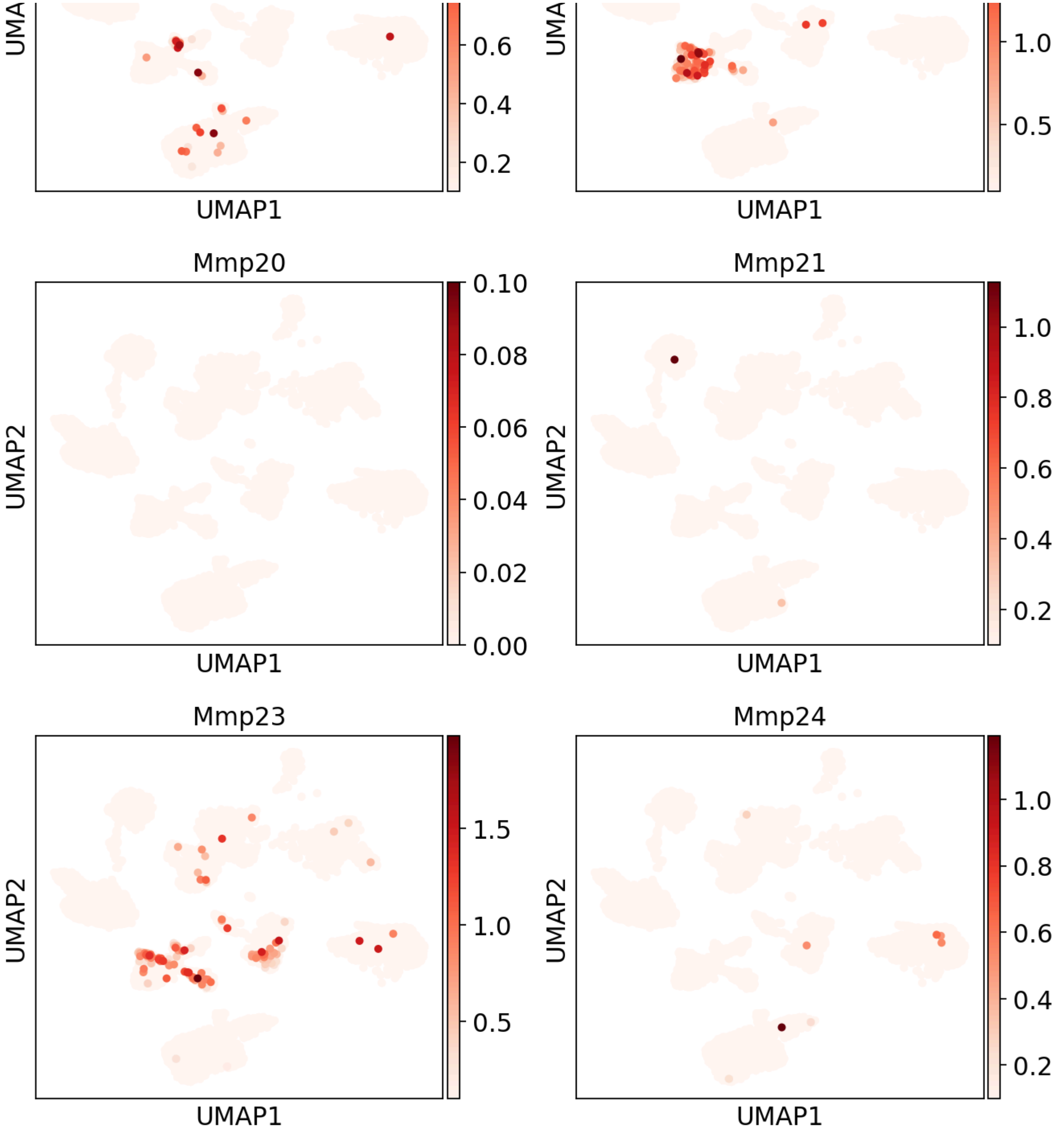
UMA
0.6
0.4
0.2
UMAP1
UMA
1.0
0.5
UMAP1
Mmp20
UMAP2
UMAP1
0.10
0.08
0.06
0.04
0.02
0.00
Mmp21
UMAP2
UMAP1
1.0
0.8
0.6
0.4
0.2
Mmp23
UMAP2
UMAP1
1.5
1.0
0.5
Mmp24
UMAP2
UMAP1
1.0
0.8
0.6
0.4
0.2

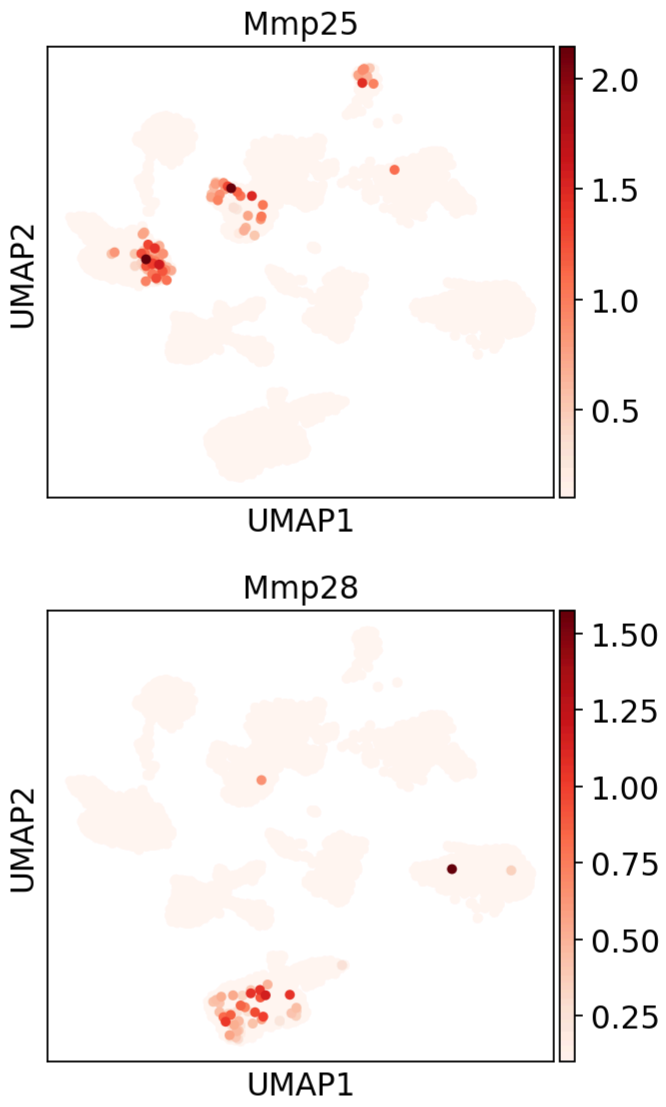

In [37]:

```
sc.pl.umap(control, color = ["Mmp2",'Mmp3','Mmp7','Mmp8',
                              "Mmp9",
                              "Mmp12",
                              "Mmp13", "Mmp14"
                              ,"Mmp19"], ncols=2, cmap =mymap, size = 80)
```

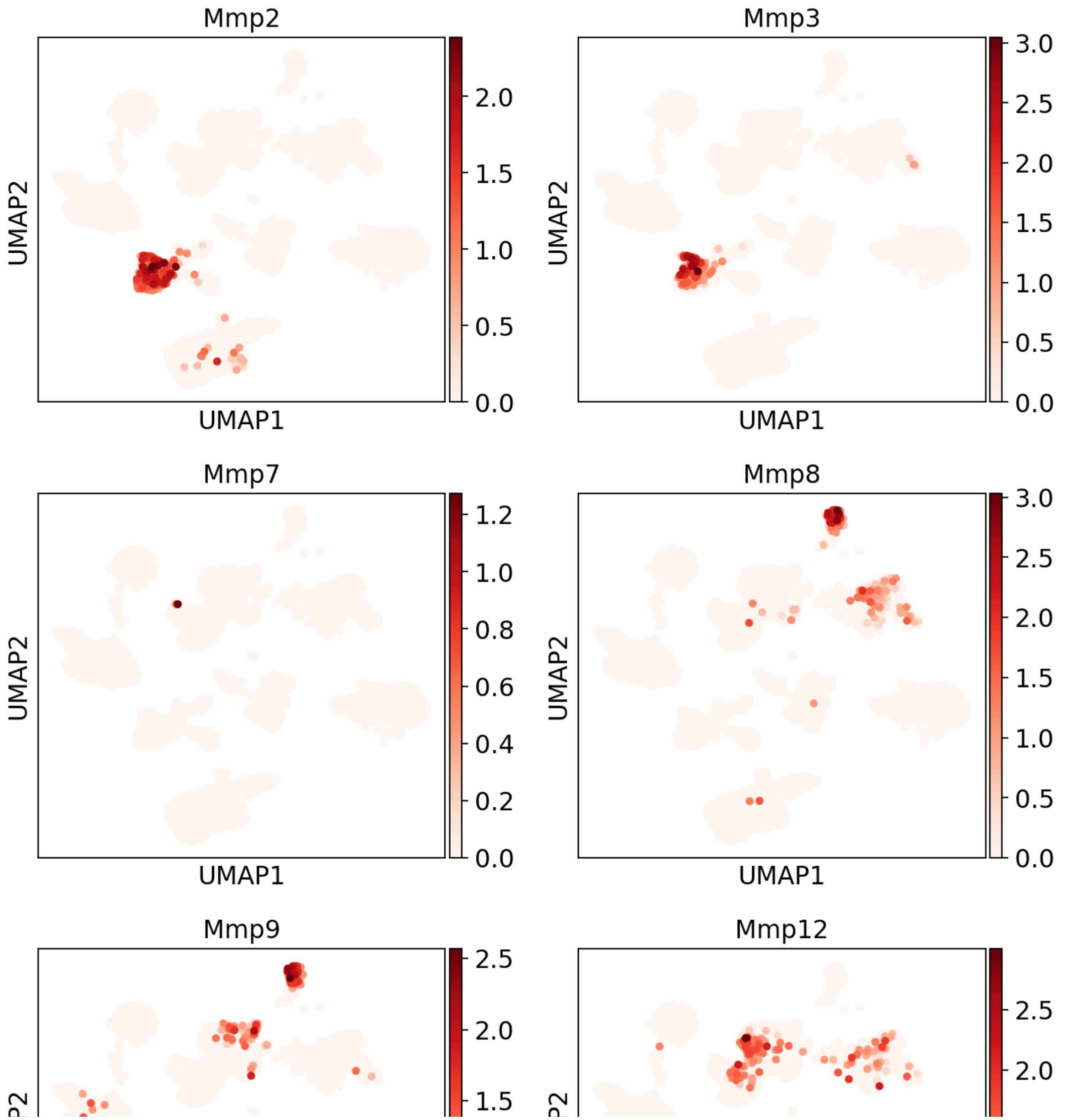
Mmp2
UMAP2
UMAP1
0.0
0.5
1.0
1.5
2.0
Mmp3
UMAP2
UMAP1
0.0
0.5
1.0
1.5
2.0
2.5
3.0
Mmp7
UMAP2
UMAP1
0.0
0.2
0.4
0.6
0.8
1.0
1.2
Mmp8
UMAP2
UMAP1
0.0
0.5
1.0
1.5
2.0
2.5
3.0
Mmp9
1.5
2.0
2.5
Mmp12
2.0
2.5

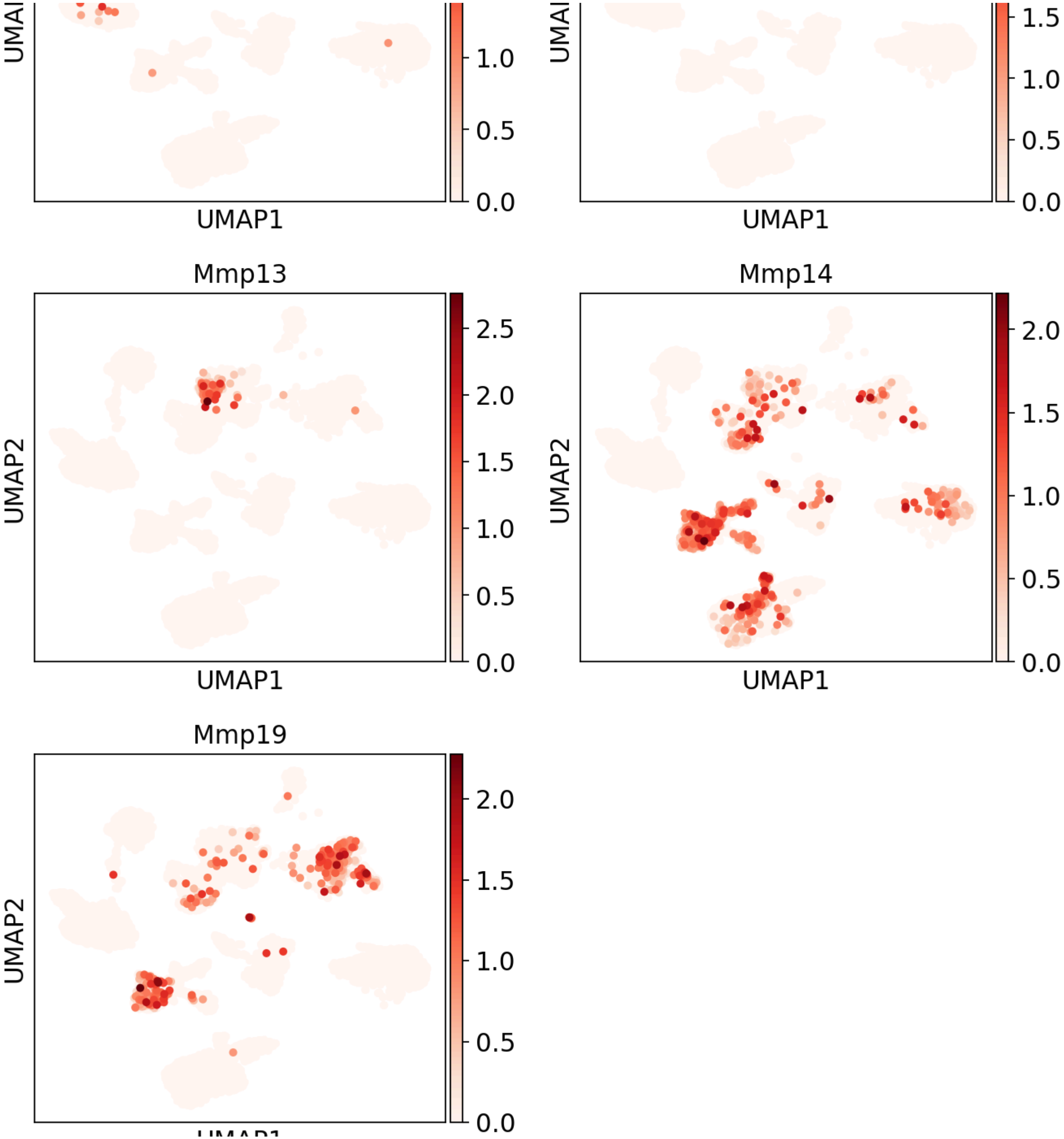
UMAP1
UMAP1
Mmp13
Mmp14
UMAP2
UMAP2
UMAP1
UMAP1
Mmp19
UMAP2
0.0
0.5
1.0
1.5
2.0
2.5

UMAP1

In [47]:
```
sc.pl.umap(control, color = ["Timp1","Timp2","Timp3","Timp4"], ncols=2, cmap =mymap, size = 80)
```

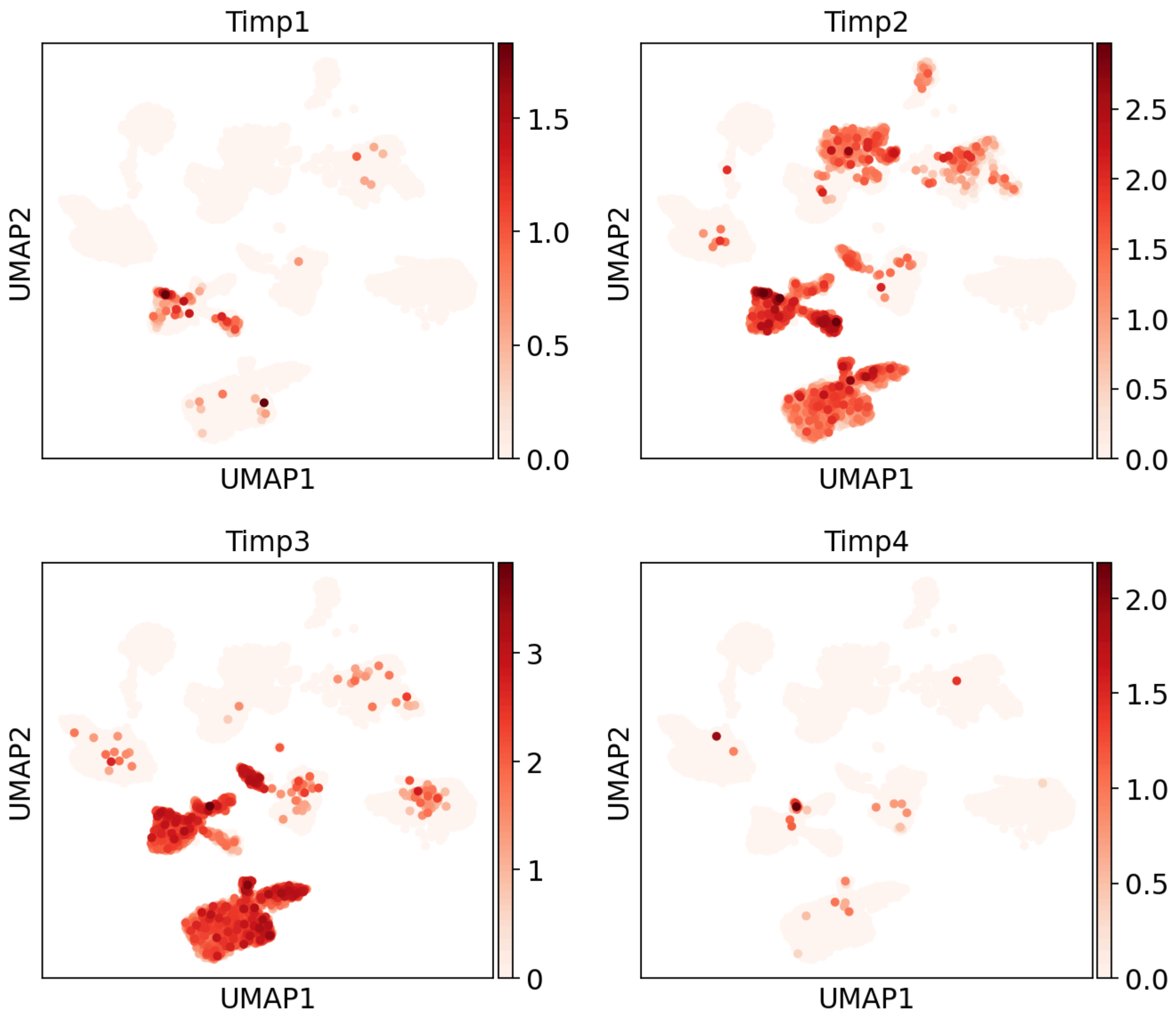

Supplement: Supplementary file 4 — Source data Fig. 3 [file 44318_2026_712_MOESM4_ESM.zip › Figure 3/RNAseq_Notebook_figure3A-B_figure4D_figure5A-B_figureEV2.pdf]
