# Supplementary material for: TRPML1 suppresses pulmonary fibrosis by limiting collagen and elastin deposition
Source: EMBO J. 2026 Feb 19;45(7):2182–209. doi: 10.1038/s44318-026-00712-4 (PMC13043727; doi:10.1038/s44318-026-00712-4)
Supplement: Supplementary file 4 — Source data Fig. 3 [file 44318_2026_712_MOESM4_ESM.zip › Figure 3/RNAseq_Notebook_figure3C-D.pdf]

In [2]:
```python
import numpy as np
import matplotlib.pyplot as plt
import scanpy as sc
import pandas as pd
import seaborn as sb

# Some custom functions
import ma_codes as codes
from ma_codes import mysize, mymap
sc.set_figure_params(vector_friendly=True, dpi_save=300)


sc.settings.verbosity = 3             # verbosity: errors (0), warnings (1), info (2), hints (3)
sc.logging.print_version_and_date()
```

```
Running Scanpy 1.8.1, on 2025-07-09 14:25.
```

In [4]:
```python
adata
```

Out[4]:
```
AnnData object with n_obs × n_vars = 160477 × 29814
    obs: 'age', 'barcode', 'batch', 'day', 'name', 'sex', 'treatment', 'identifier', 'n_counts', 'n_genes', 'percent_mito', 'doublet_scores', 'size_factors', 'S_score', 'G2M_score', 'phase', 'time_point', 'leiden_1', 'leiden_2', 'domain', 'cell_type', 'ct_level2', 'compartment_label', 'meta_label', 'age_treatment', 'age_day'
    var: 'n_cells', 'highly_variable', 'means', 'dispersions', 'dispersions_norm', 'highly_variable_nbatches', 'highly_variable_intersection'
    uns: 'age_colors', 'age_day_colors', 'age_treatment_colors', 'batch_colors', 'cell_type_colors', 'compartment_label_colors', 'ct_level2_colors', 'day_colors', 'dendrogram_cell_type', 'domain_colors', 'hvg', 'leiden', 'leiden_1_colors', 'leiden_2_colors', 'meta_label_colors', 'name_colors', 'neighbors', 'pca', 'sex_colors', 'time_point_colors', 'treatment_colors', 'umap'
    obsm: 'X_pca', 'X_umap'
    varm: 'PCs'
    layers: 'counts', 'unsouped_counts'
    obsp: 'connectivities', 'distances'
```

In [6]:
```python
sc.pl.umap(adata, color = ["treatment"], wspace = 0.2, size = 20,cmap=mymap )
```

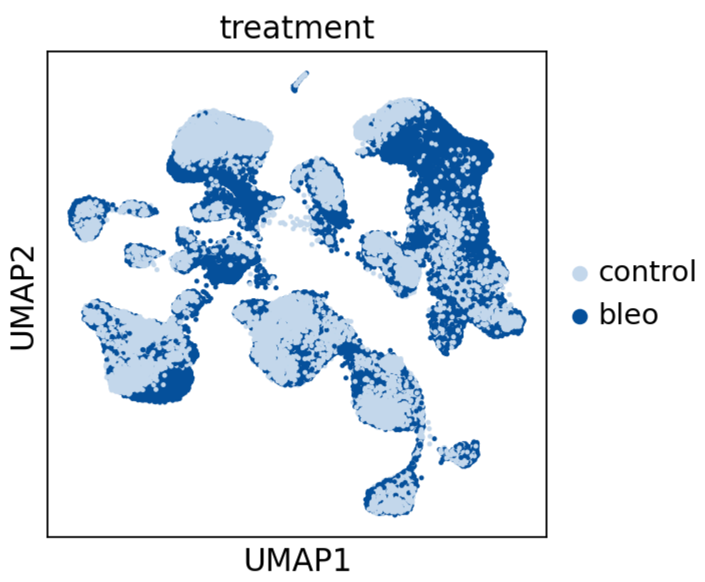

In [7]:
```
control = adata[adata.obs.treatment.isin(["control"])].copy()

sc.pl.umap(control, color = ["treatment"], wspace = 0.2, size = 20)
```

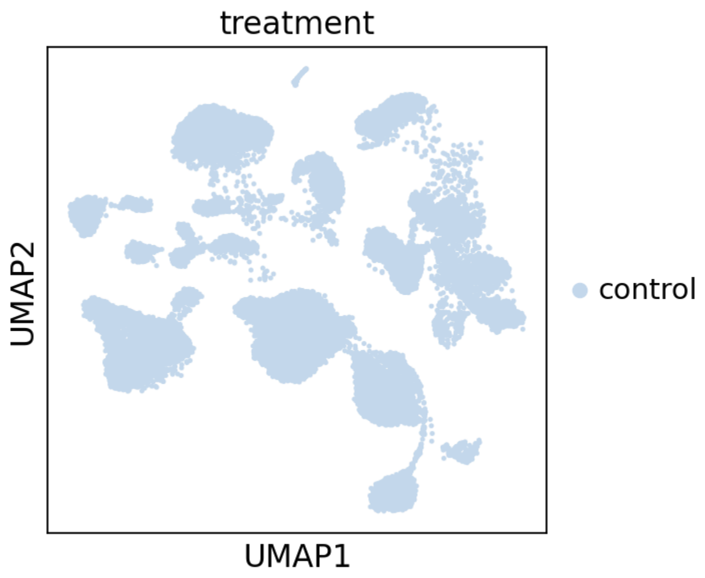

In [15]:
```
control
```

Out[15]:
```
AnnData object with n_obs × n_vars = 53778 × 29814
    obs: 'age', 'barcode', 'batch', 'day', 'name', 'sex', 'treatment', 'identifier', 'n_counts', 'n_genes', 'percent_mito',
'doublet_scores', 'size_factors', 'S_score', 'G2M_score', 'phase', 'time_point', 'leiden_1', 'leiden_2', 'domain', 'cell_typ
e', 'ct_level2', 'compartment_label', 'meta_label', 'age_treatment', 'age_day'
    var: 'n_cells', 'highly_variable', 'means', 'dispersions', 'dispersions_norm', 'highly_variable_nbatches', 'highly_variab
le_intersection'
    uns: 'age_colors', 'age_day_colors', 'age_treatment_colors', 'batch_colors', 'cell_type_colors', 'compartment_label_color
s', 'ct_level2_colors', 'day_colors', 'dendrogram_cell_type', 'domain_colors', 'hvg', 'leiden', 'leiden_1_colors', 'leiden_2_
colors', 'meta_label_colors', 'neighbors', 'pca', 'sex_colors', 'time_point_colors', 'treatment_colors', 'umap'
    obsm: 'X_pca', 'X_umap'
    varm: 'PCs'
    layers: 'counts', 'unsouped_counts'
    obsp: 'connectivities', 'distances'
```

In [14]:
```
sc.pl.dotplot(control, ["Mcoln1"], groupby ="cell_type",dot_max=0.2)
```

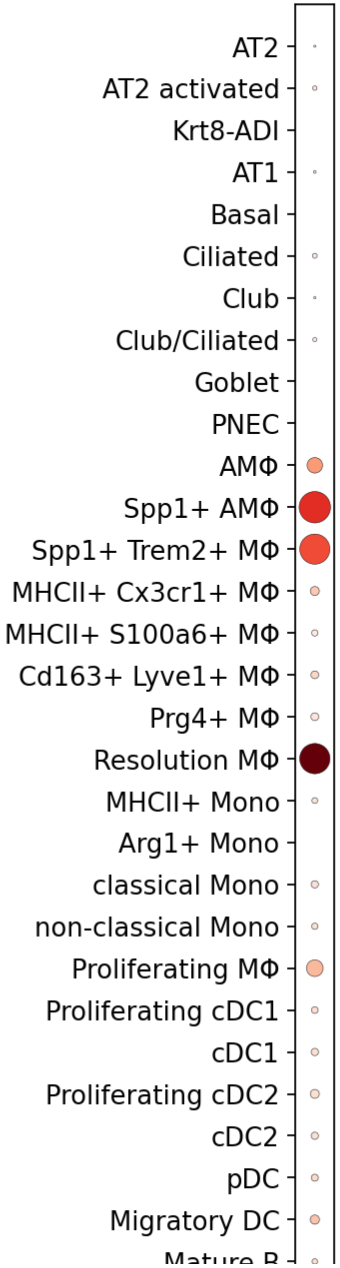
AT2
AT2 activated
Krt8-ADI
AT1
Basal
Ciliated
Club
Club/Ciliated
Goblet
PNEC
AMΦ
Spp1+ AMΦ
Spp1+ Trem2+ MΦ
MHCII+ Cx3cr1+ MΦ
MHCII+ S100a6+ MΦ
Cd163+ Lyve1+ MΦ
Prg4+ MΦ
Resolution MΦ
MHCII+ Mono
Arg1+ Mono
classical Mono
non-classical Mono
Proliferating MΦ
Proliferating cDC1
cDC1
Proliferating cDC2
cDC2
pDC
Migratory DC

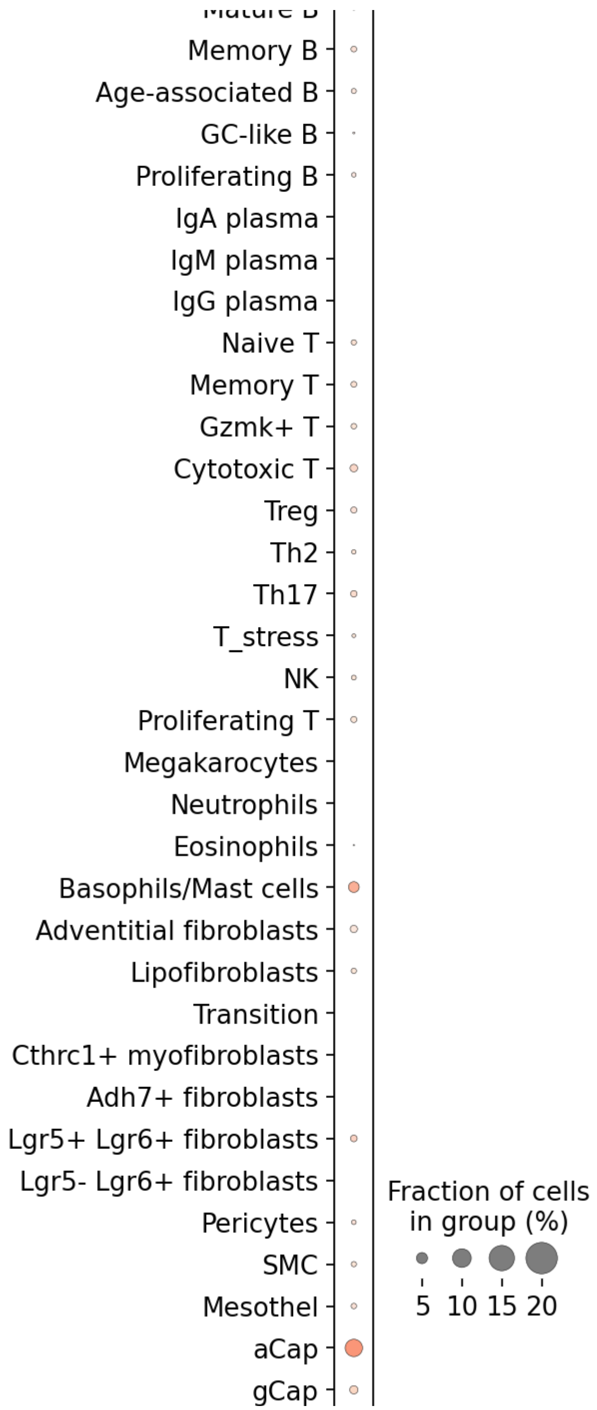
Memory B
Age-associated B
GC-like B
Proliferating B
IgA plasma
IgM plasma
IgG plasma
Naive T
Memory T
Gzmk+ T
Cytotoxic T
Treg
Th2
Th17
T_stress
NK
Proliferating T
Megakaryocytes
Neutrophils
Eosinophils
Basophils/Mast cells
Adventitial fibroblasts
Lipofibroblasts
Transition
Cthrc1+ myofibroblasts
Adh7+ fibroblasts
Lgr5+ Lgr6+ fibroblasts
Lgr5- Lgr6+ fibroblasts
Pericytes
SMC
Mesothel
aCap
gCap
Fraction of cells in group (%)
5
10
15
20

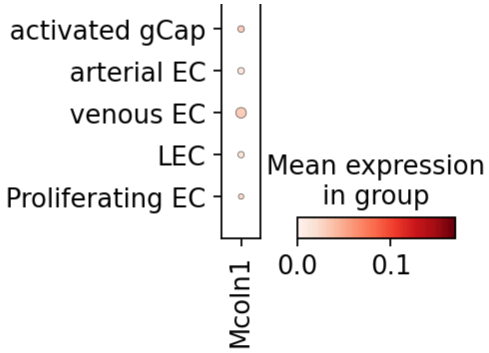
activated gCap
arterial EC
venous EC
LEC
Proliferating EC
Mcoln1
Mean expression
in group
0.0
0.1

In [ ]:
