# Supplementary material for: TRPML1 suppresses pulmonary fibrosis by limiting collagen and elastin deposition
Source: EMBO J. 2026 Feb 19;45(7):2182–209. doi: 10.1038/s44318-026-00712-4 (PMC13043727; doi:10.1038/s44318-026-00712-4)
Supplement: Supplementary file 14 — Expanded View Figures [file 44318_2026_712_MOESM14_ESM.pdf]

Expanded View Figures

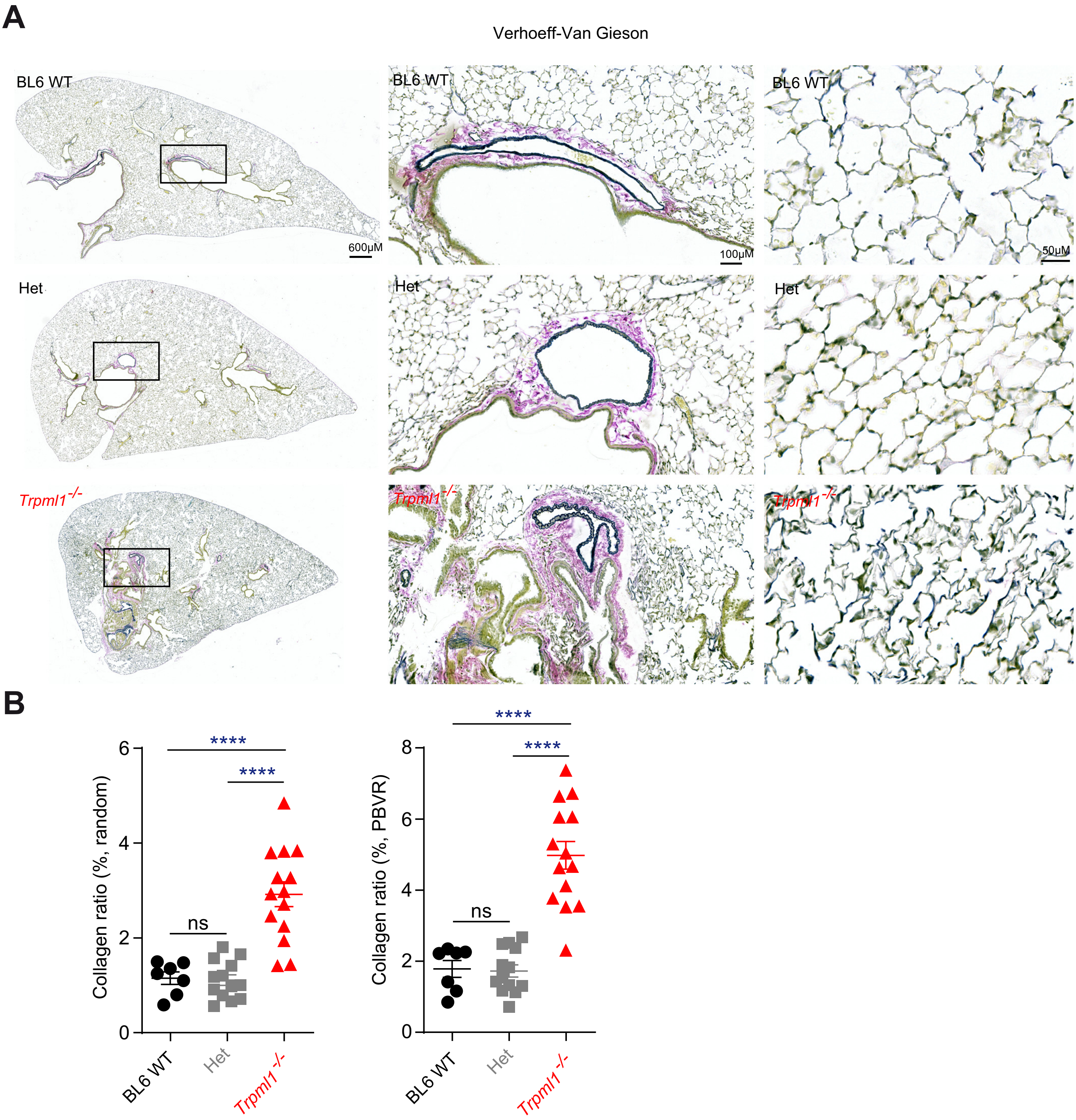

Figure EV1. Quantification of collagen and elastin using Verhoeff-Van Gieson staining (VVG).

(**A**) VVG-stained lung tissue sections from untreated BL6 WT and *Trpml1*$^{-/-}$ mouse lungs. Elastic fibers are stained blue-black, collagen appears red and other tissue elements yellow. The quantification of the elastin fibers as counts per field in the VVG-stained lung tissue sections (7–14 mice per group) is shown in Fig. 1I. (**B**) Quantification of collagen as part of the VVG-stained lung tissue section analysis from untreated BL6 WT and *Trpml1*$^{-/-}$ mouse lungs as shown in (**A**). For quantification five selected fields of view per lung (7–14 mice per group) were analysed, with each point representing the mean per mouse. ***$p < 0.001$; One-way ANOVA followed by Tukey's post hoc test. Data were mean ± SEM. Exact *p* values were: Col1a1 ratio (%, random), WT vs KO, $p < 0.0001$; Het vs KO, $p < 0.0001$; Col1a1 ratio (%, PBVR), WT vs KO, $p < 0.0001$; Het vs KO, $p < 0.0001$. Source data are provided as a Source Data file. Source data are available online for this figure.

© The Author(s)

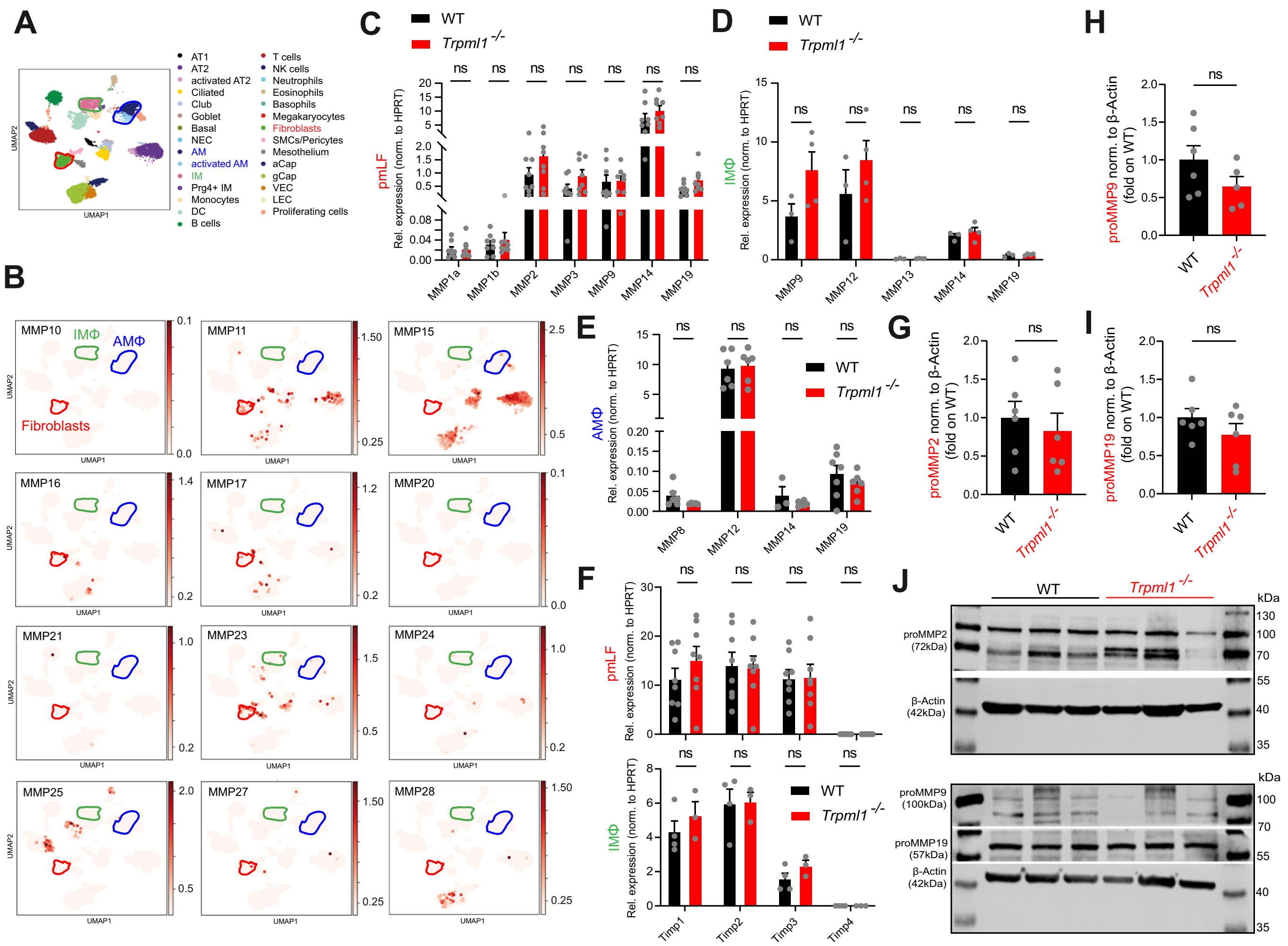

Figure EV2. Characterization of the expression of additional MMPs in the murine lung using single-cell transcriptomics and qPCR/WB data of MMPs and TIMPs.

(**A**) UMAP plot showing annotated cell clusters. Major cell types are labeled and color-coded, including epithelial, immune, endothelial, and stromal populations. MMP expression was determined in 32 different cell types. (**B**) UMAP plots showing the expression of selected MMP genes (MMP10, 11, 15, 16, 17, 20, 21, 23, 24, 25, 27, 28) in single cells from mouse lung tissue (filtered air group, $n = 9$). Expression is color-coded by log-normalized expression levels, with darker shades indicating higher expression. (**C–E**) qRT-PCR data showing mRNA expression levels of Mmp1a, Mmp1b, Mmp2, Mmp3, Mmp9, Mmp12, Mmp13, Mmp14, and Mmp19 in pmLF, IMΦ or AMΦ (WT and *Trpml1*$^{-/-}$). (**F**) qRT-PCR data showing mRNA expression levels of Timp1, Timp2, Timp3, Timp4 in pmLF and IMΦ (WT and *Trpml1*$^{-/-}$). In all figures, each single dot corresponds to one biologically independent sample. Data were mean ± SEM. Statistical analysis for qRT-PCR data were performed with multiple *t*-test, corrected for multiple comparisons using the Holm–Šídák method. (**G–J**) Western Blot analysis of different MMPs in pmLF isolated from WT and *Trpml1*$^{-/-}$ mice. Graphs show quantification of each MMP band normalized to ß-actin. Each single dot corresponds to cells isolated from one mouse. Data were mean ± SEM. Student's *t*-test, unpaired, two-tailed. Source data are available online for this figure.

© The Author(s)

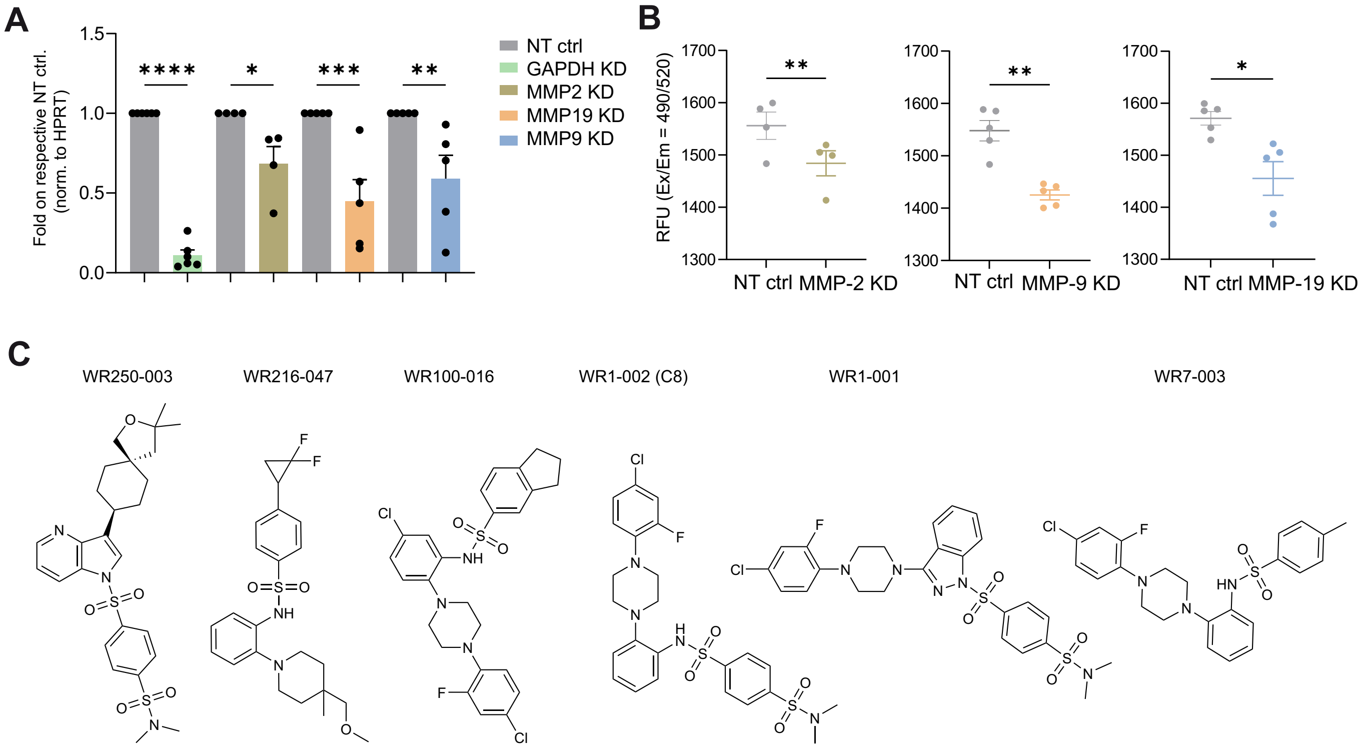

Figure EV3. MMP knockdown experiments, zymography assay and structures of TRPML1 agonists.

(**A**) Knockdown efficiency of the targeted gene determined by qPCR, shown as fold change relative to the non-targeting (NT) control and normalized to the housekeeping gene ($n = 4$–6 biological replicates). Data were mean ± SEM. Statistical significance was determined using two-way ANOVA followed by Holm–Šídák's multiple comparisons test. Shown are mean values ± SEM. $^{**}p < 0.01$, $^{****}p < 0.0001$. Exact p values were: GAPDH, $p < 0.0001$; MMP2, $p = 0.0154$; MMP9, $p < 0.0001$; MMP19, $p = 0.0016$. (**B**) Zymography-based MMP activity measured as absolute final fluorescence values, representing the total collagen degraded in each sample at reaction endpoint ($n = 4$–6 biological replicates). Data were mean ± SEM. Statistical significance was determined using a paired t-test, comparing non-targeting control and MMP knockdown from the same mouse. Statistical significance was defined as $^{*}p < 0.05$ and $^{**}p < 0.01$. Exact p values were: MMP2, $p = 0.0017$; MMP9, $p = 0.0059$; MMP19, $p = 0.0413$. (**C**) Shown are the chemical structures of TRPML1 compounds used in this study, provided by Casma Therapeutics. Source data are available online for this figure.

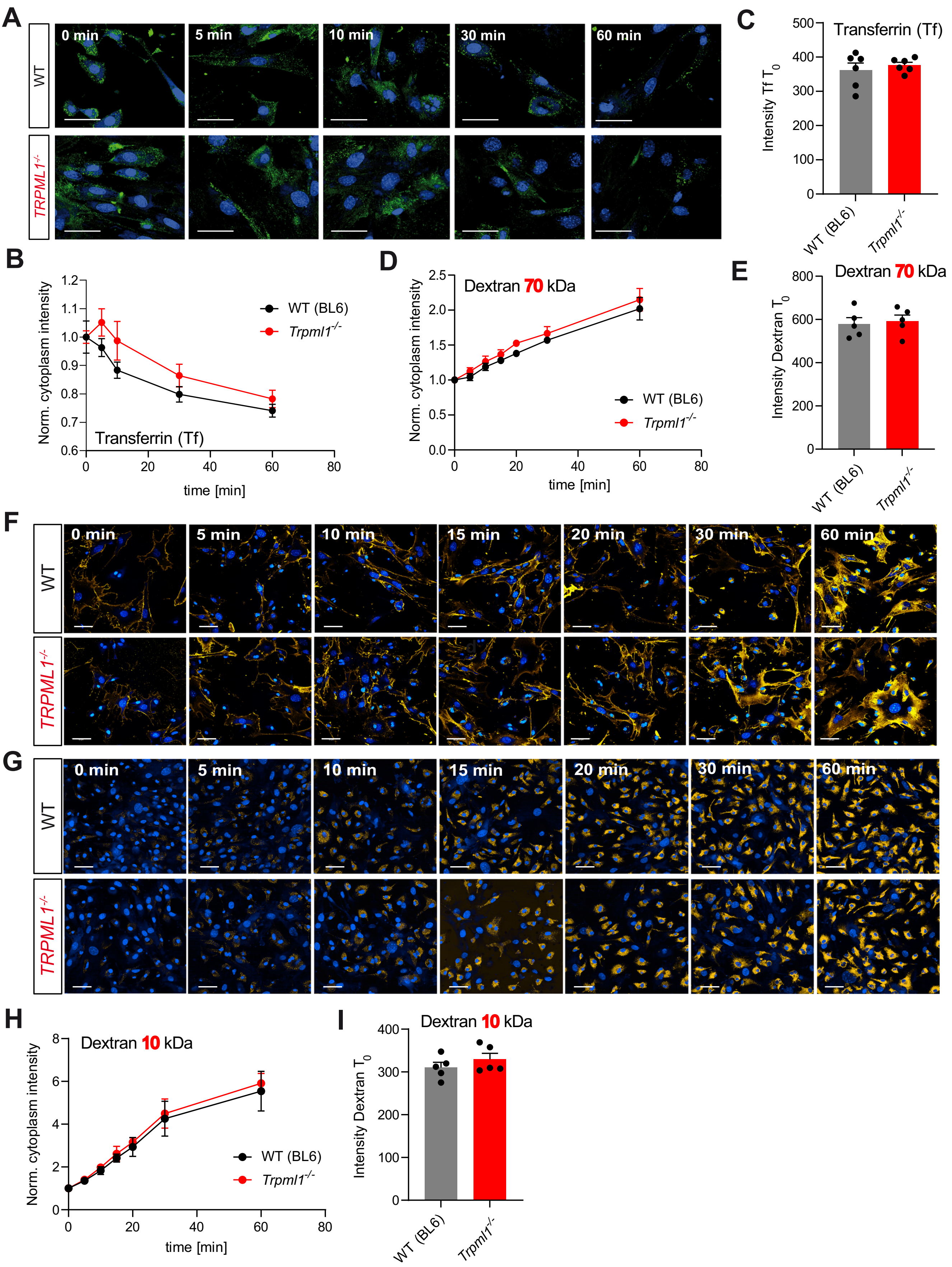
A
0 min
5 min
10 min
30 min
60 min
WT
TRPML1-/-
B
Norm. cytoplasm intensity
Transferrin (Tf)
time [min]
WT (BL6)
Trpml1-/-
C
Transferrin (Tf)
Intensity Tf T0
WT (BL6)
Trpml1-/-
D
Dextran 70 kDa
Norm. cytoplasm intensity
time [min]
WT (BL6)
Trpml1-/-
E
Dextran 70 kDa
Intensity Dextran T0
WT (BL6)
Trpml1-/-
F
0 min
5 min
10 min
15 min
20 min
30 min
60 min
WT
TRPML1-/-
G
0 min
5 min
10 min
15 min
20 min
30 min
60 min
WT
TRPML1-/-
H
Dextran 10 kDa
Norm. cytoplasm intensity
time [min]
WT (BL6)
Trpml1-/-
I
Dextran 10 kDa
Intensity Dextran T0
WT (BL6)
Trpml1-/-

 © The Author(s)

Figure EV4. Transferrin and dextran trafficking, and endocytosis in WT and *Trpml1*$^{-/-}$ pmLF and macrophages.

(**A–C**) Transferrin (Tf) trafficking assay results. Images (scale bar 50 µm) (**A**) and graphs (**B**, **C**) demonstrate Tf-recycling kinetics in *Trpml1*$^{-/-}$ and WT pmLF, showing the decrease of Tf mean fluorescence after the 20 min pulse (measures Tf accumulation). Shown are normalized mean values ± SEM from six mice per group performed in technical triplicates. Two-way ANOVA followed by Šídák's post hoc test was applied in (**B**). (**C**) Tf mean fluorescence in pmLF (WT versus Trpml1$^{-/-}$) after 20 min pulse with Tf Alexa Fluor 488 (0 min timepoint, measures Tf uptake). Data were shown as mean ± SEM from six mice per group performed in technical triplicates. Student's *t*-test, unpaired, two-tailed was applied. (**D**) Quantification of dextran 70 kDa uptake showing rates of endocytosis in *Trpml1*$^{-/-}$ compared to WT pmLF at various time points. Data were shown as mean ± SEM from five mice per group performed in technical triplicates. (**E**) Dextran 70 kDa Alexa Fluor 568 mean fluorescence in pmLF (WT versus *Trpml1*$^{-/-}$) at timepoint 0 min. ($n = 4$ biological replicates). Shown are mean values ± SEM. (**F–G**) Representative Opera Phenix confocal images obtained from endocytosis experiments using dextran 70 and 10 kDa probes, respectively. Images show pmLF (WT versus *Trpml1*$^{-/-}$) that have been treated with fluorescently labeled dextran for different time intervals. Scale bar 50 µm. (**H**) Quantification of dextran 10 kDa uptake showing rates of endocytosis in *Trpml1*$^{-/-}$ compared to WT pmLF at time points from 0 to 60 min. Two-way ANOVA followed by Šídák's post hoc test was applied. (**I**) Dextran 10 kDa mean fluorescence in pmLF (WT versus *Trpml1*$^{-/-}$) (0 min timepoint). Data were shown as mean ± SEM from five mice per group performed in technical triplicates. Student's *t*-test, unpaired, two-tailed was applied. Source data are provided as a Source Data file. Source data are available online for this figure.

© The Author(s)

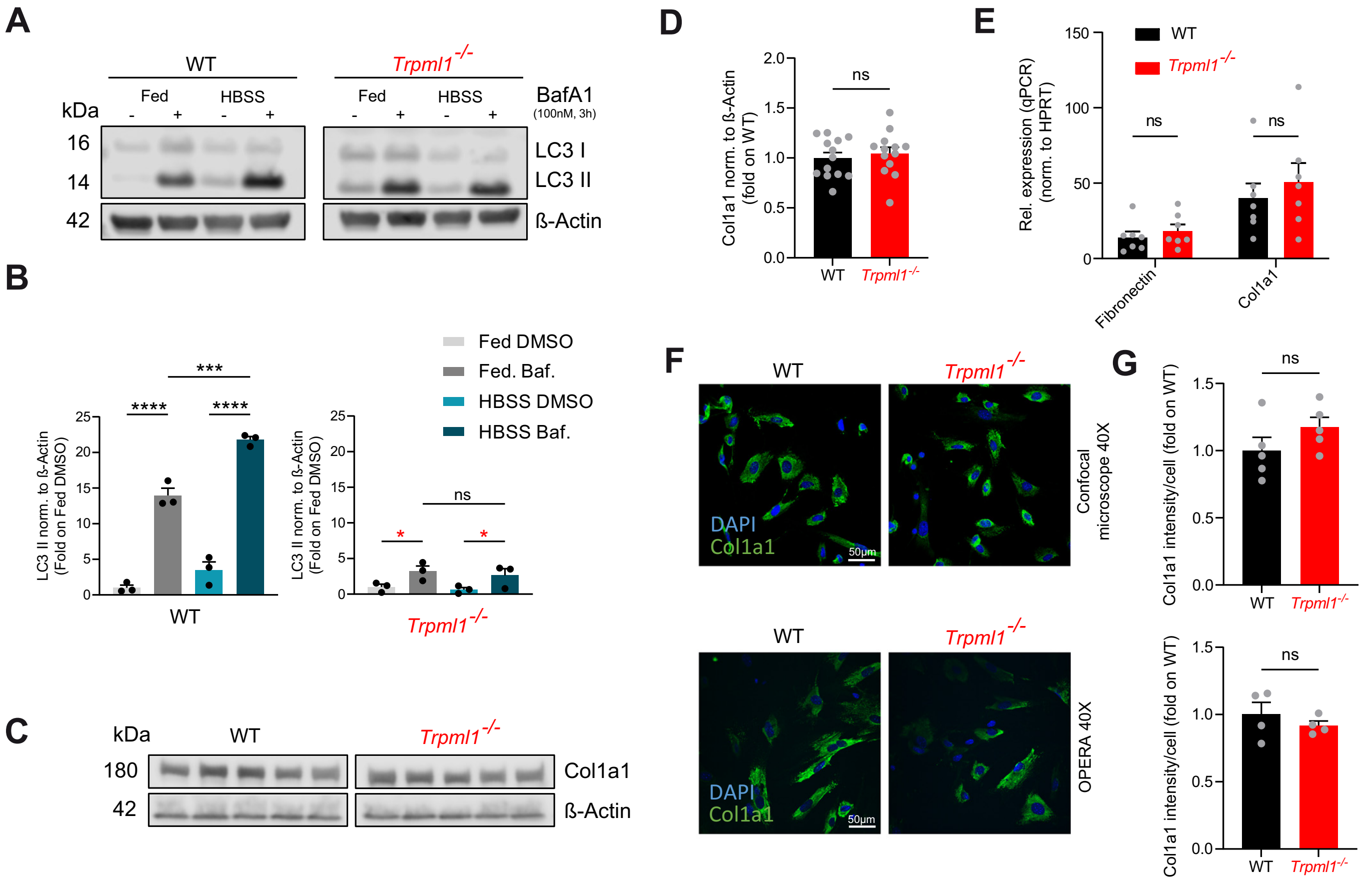

Figure EV5. Autophagy and intracellular collagen.

(**A**, **B**) Western Blot analysis of endogenous LC3 (LC3I-II), treated with DMSO or bafilomycin A1 (100 nM) for 3 h, under fed (complete media) or starvation (HBSS) conditions in pmLF isolated from WT and *Trpml1*$^{-/-}$ mice. Graphs show quantification of LC3-II band intensity, normalized to β-actin level. Each single dot corresponds to cells isolated from one mouse. Data were shown as mean ± SEM. *$p < 0.05$, ***$p < 0.001$, ****$p < 0.0001$; Two-way ANOVA followed by Tukey's post hoc test. Exact p values were: LC3-II in WT, comparison of Fed DMSO to Fed. Baf., $p < 0.0001$; Comparison of Fed. Baf. To HBSS Baf., $p = 0.0002$; Comparison of HBSS DMSO to HBSS Baf., $p < 0.0001$; LC3-II in KO, comparison of Fed DMSO to Fed. Baf., $p = 0.0109$; Comparison of HBSS DMSO to HBSS Baf., $p = 0.0171$. (**C**) qRT-PCR data showing mRNA expression levels of fibronectin and Col1a1 (WT and *Trpml1*$^{-/-}$). Data were mean ± SEM. Two-way ANOVA followed by Bonferroni post hoc test. (**D**, **E**) Western blot analysis of Col1a1 in pmLF isolated from WT and *Trpml1*$^{-/-}$ mice. Graphs show quantification of Col1a1 bands normalized to ß-actin. Each single dot corresponds to cells isolated from one mouse. Data were mean ± SEM. Student's *t*-test, unpaired, two-tailed. (**F**, **G**) Confocal microscope and Opera Phenix images, and quantification of pmLF stained with Col1a1 (green) and DAPI (blue). Graphs show Col1a1 intensity per cell normalized to WT cells. Each single dot corresponds to cells isolated from one mouse. Data are mean ± SEM. Student's *t*-test, unpaired, two-tailed. Source data are provided as a Source Data file. Source data are available online for this figure.

 © The Author(s)
